# Evolutionary conservation analysis reveals a primordial function of STING as a regulator of lipid metabolism via FADS2

**DOI:** 10.1038/s41467-026-76833-5

**Published:** 2026-08-14

**Authors:** Soumyabrata Guha, Joanna Re, Stéphane Grégoire, Morgane Chemarin, Adeline Augereau, Yasmine Messaoud-Nacer, Pierre Le Hars, Hanane Chamma, Victoria Bergas, Collin McNairy, Jenny Nguyen, Jennifer Barrat, Jean-Paul Pais de Barros, Arielle Woznica, Niyazi Acar, Isabelle K. Vila, Pierre Boudinot, Nadine Laguette

**Affiliations:** 1 Institut de Génétique Moléculaire de Montpellier (IGMM), Université de Montpellier, CNRS UMR5535, Montpellier, France; 2 Eye & Nutrition Research Group, Université Bourgogne Europe, CHU Bourgogne, Public Health Department, Institut Agro, CNRS, INRAE, UMR CSGA, Dijon, France; 3 Université Bourgogne Europe, INSERM, BioSanD US 58, Plateforme Diviomics, Dijon, France; 4 The University of Texas at Austin, Department of Molecular Biosciences, Austin, TX USA; 5 Université de Strasbourg, CNRS UPR9022, Institut de Biologie Moléculaire et Cellulaire, Strasbourg, France; 6 Université Paris-Saclay, INRAE, UVSQ, VIM, Jouy-en-Josas, France

**Keywords:** Inflammation, Molecular evolution, Pattern recognition receptors

## Abstract

The stimulator of interferon genes (STING) is a pivotal regulator of type I interferon (IFN) responses. Although the IFN system is confined to vertebrates, STING is present across metazoans and in some unicellular eukaryotes, suggesting involvement in distinct functions prior to vertebrate divergence. Here we explore the conservation of STING-mediated regulation of polyunsaturated fatty acid (PUFA) metabolism. We find that STING homologs from vertebrates, invertebrates, and unicellular eukaryotes interact with fatty acid desaturase 2 (FADS2), the rate-limiting enzyme in PUFA metabolism, and influence subsequent functional outputs. Specifically, we show that STING homologs differentially shape cell susceptibility to infection by DNA and RNA viruses independently of the activation of IFN responses, suggesting that STING-mediated metabolic pathway regulation may participate in primitive host defense mechanisms. Thus, we identify STING-mediated metabolic regulation as an evolutionarily conserved feature and a primordial function of STING.

## Abstract (web summary)

STING is a well-known activator of type I interferon (IFN-I) responses. However, it is debated whether STING has an ancestral function independent of interferon responses. Here, by combining evolutionary comparative analysis and lipidomics, the authors show that STING homologs regulate polyunsaturated fatty acid (PUFA) metabolism via FADS2 and that this primordial STING-mediated metabolic control regulates responses to viral infections independently of antiviral IFN-I signaling.

## Introduction

The endoplasmic reticulum (ER)-resident stimulator of interferon genes (STING) adaptor protein is a central regulator of innate immune responses, initially identified as responsible for the induction of type I interferons (IFN) in response to cytosolic double-stranded deoxyribonucleic acid (dsDNA) in mammals^1,2^. This function of STING is initiated following the detection of exogenous and endogenous cytosolic dsDNA by the cyclic guanosine monophosphate (GMP)-adenosine monophosphate (AMP) (cGAMP) synthase (cGAS) pattern recognition receptor^3,4^. Upon activation, cGAS catalyzes the production of the 2'3’-cGAMP cyclic dinucleotide (CDN), which subsequently binds to STING^5^, inducing its oligomerization^6^, translocation from the ER to the Golgi apparatus^7^, and assembly of the STING signalosome^5,8^. The latter canonically comprises the TANK binding kinase 1 (TBK1) that catalyzes the activating phosphorylation of STING^8^, as well as of transcription factors, such as interferon regulatory factors (IRF)^5,9,10^, and culminates in the production of inflammatory cytokines and chemokines alongside type I IFNs^1^. Other well-documented immune-related functions of STING are all triggered by agonist-mediated STING activation and comprise the activation of the nuclear factor kappa B (NF-κB) signaling cascade^11^, and of non-canonical autophagy^12,13^. Thus, STING activation is essential to shaping innate immune responses to dsDNA.

Analysis of the cGAS and STING encoding genes and of their immune-related functions in the choanoflagellate *Monosiga brevicollis* revealed that the eukaryotic cGAS-STING signaling pathway arose in unicellular eukaryotes over 600 million years ago, before the emergence of ancestral metazoans^14^. STING homologs have been discovered in most metazoan phyla (with some exceptions such as nematodes), unicellular choanoflagellates, and even bacteria^15^, but neither its structure nor its canonical function, are strictly conserved across the tree of life. Indeed, to trigger pro-inflammatory signaling, the prototypical STING protein relies on three main domains^16^: four N-terminal transmembrane domains (TMD) ensure the anchoring of the protein to membranes, followed by a central CDN binding domain (CBD), and a C-terminal tail (CTT) that is essential for TBK1 and transcription factors activation^8,17^. While the tertiary structure and overall architecture of the CBD and TMD are well-conserved, the CTT presents significant differences across species. For instance, invertebrate STING lacks a CTT^16^, with few possible exceptions such as the oyster *Crassostrea gigas* (mollusk) and shrimp *Litopenaeus vannamei* (crustacean)^18^, while the CTT of *Danio rerio* (zebrafish) STING is longer than that of mammals (e.g., *Mus musculus* or *Homo sapiens*). In addition, amphibian STING (both Anura and Urodela) exhibit a vestigial CTT, suggestive of secondary loss^16,19,20^. In contrast, despite low amino acid sequence similarity, the CBD of *Crassostrea gigas* (mollusk), *Drosophila melanogaster* (insect), and *Monosiga brevicollis* (choanoflagellate) can all bind CDNs, even though the specific type of CDN differs among species^14,15,21^. Interestingly, while most STING homologs present a TMD, that of *Crassostrea gigas* instead presents a Toll/IL1 receptor (TIR) domain^15^ and a short CTT-like domain of very low sequence similarity with vertebrate CTT. In line with the poor conservation of the CTT, the bona fide IFN system originated much later in evolution than the CDN-STING interaction, is restricted to jawed vertebrates, and is absent in agnathans and basal chordates^22^. In contrast, NF-κB and 2’3’-cGAMP-induced non-canonical autophagy pathways are highly conserved in basal metazoans such as *Nematostella vectensis* (cnidarian)^12,23^. Overall, the prevailing hypothesis is thus that STING fulfilled ancestral immune-related functions, via autophagy and NF-κB induction, prior to emergence of the IFN system.

STING-mediated lipid metabolism regulation is the main characterized homeostatic function of STING, that, as opposed to immune related functions, is disrupted by STING stimulation^24,25^. Nonetheless, its evolutionary conservation has not been assessed yet. In *Mus musculus*, STING controls metabolic homeostasis by inhibiting the enzymatic activity of the fatty acid desaturase 2 (FADS2)^24^. FADS2 is a Δ6-desaturase that catalyzes the rate-limiting step in the omega-6 and omega-3 polyunsaturated fatty acid (PUFA) biosynthesis pathway^26^. The action of FADS2 leads to the generation of long chain PUFAs (LC-PUFA) that serve as ligands for metabolic transcription factors^27,28^, or are further processed into signaling lipids called oxylipins^29–31^. *Mus musculus* STING ablation consequently leads to accumulation of FADS2 products, including LC-PUFAs and oxylipins, promoting metabolic rewiring in vivo^24^. The ability of STING to regulate lipid homeostasis is also documented in *Homo sapiens* and in *Drosophila melanogaster*^32^. Interestingly, STING haplotypes have been reported in human populations that are deficient for IFN response induction, but competent for lipid metabolism regulation and FADS2 interaction^24,33^, suggesting conservation of this homeostatic function. This role of STING in the regulation of lipid metabolism is profoundly disturbed in conditions of STING activation^24^, where STING is displaced from the ER where it regulates FADS2, to the Golgi apparatus and subsequently degraded^11^.

Since STING predates the evolution of IFN-based immunity^14,15^, is present in basal metazoans^23^, has been shown to have a critical role in lipid metabolism^24,32–34^, and has lost its ability to induce IFN-response secondarily in several instances^19,33^ (suggesting that this function might be dispensable under certain circumstances), we hypothesize that the role of STING in metabolic regulation may be its primordial function.

To test this hypothesis, we analyze the conservation of the FADS2-STING interaction and of the associated functional output. This reveals that not only is the STING-FADS2 interaction conserved in various holozoan lineages, but also differentially regulates FADS2 activity and broader lipid metabolism. This function of STING ultimately shapes cellular response and susceptibility to infection by DNA and RNA viruses independently of IFN responses.

## Results

### Rationale for the choice of STING homologs

**Fig. 1:**
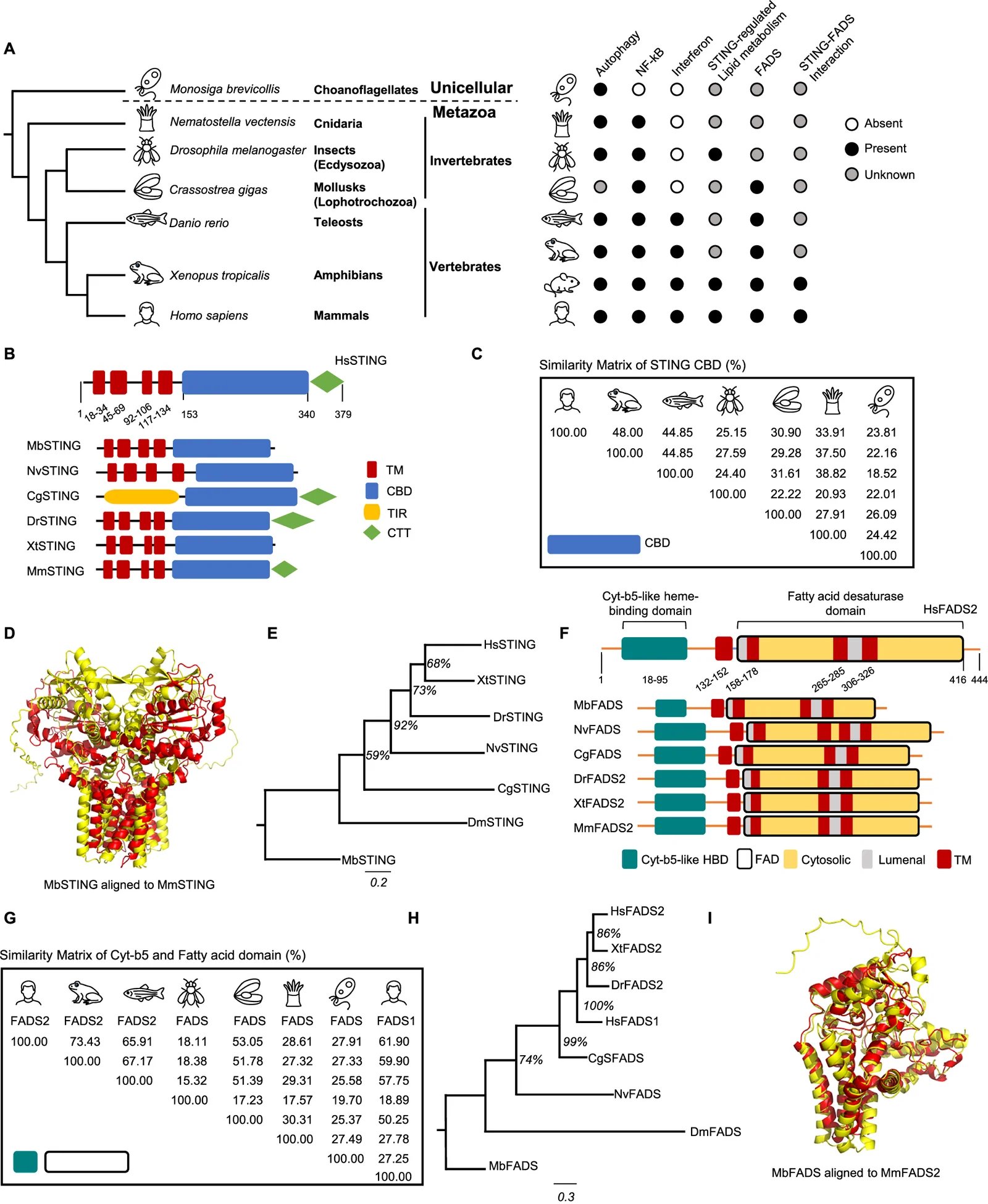
Comparative analyses of STING and FADS homologs. **A** Schematic evolutionary tree of representative species showing phylogenetic relationships (branch lengths are not to scale) with table showing conservation of STING effectors/functions, i.e. STING-dependent autophagy, NF-κB activation, interferon activation, lipid metabolism regulation, and interaction with FADS2. **B** Structure of STING domains in different lineages. The key to domains representation is shown under the panel. CTT C-terminal tail, STING stimulator of interferon genes, TIR Toll and IL-1 receptor, CDN cyclic dinucleotide, CBD cyclic dinucleotide binding domain. **C** Matrix of sequence similarity of STING CBD domains (from Clustal omega at ebi.ac.uk). **D** Sequence-independent structure-based alignment of MbSTING (red) and MmSTING (yellow) rendered using Pymol. **E** Evolutionary tree (unrooted) of STING homologs inferred by Maximum Likelihood method and JTT matrix-based model. The tree with the highest log likelihood (−6633.58) is shown. The percentage of trees in which the associated taxa clustered together is shown next to the branches. Evolutionary analyses were conducted in MEGA X. **F** FADS2 and FADS-like proteins domain structure in the representative species. The key to domains representation is shown under the panel. FAD fatty acid desaturase, HBD heme binding domain, TM transmembrane domain. **G** Matrix of sequence identity of the region comprising CytB5 and FAD domains (from Clustal omega at ebi.ac.uk). **H** Evolutionary tree inferred by Maximum Likelihood method and JTT matrix-based model. The tree with the highest log likelihood (−6652.47) is shown. The percentage of trees in which the associated taxa clustered together is shown next to the branches. Evolutionary analyses were conducted in MEGA X. **I** Structure-based alignment of MbFADS (red) and MmFADS2 (yellow) done using Pymol.

Based on the presence of key domains and on the ability to activate downstream effectors, human STING homologs in eukaryotes can be divided into 3 broad classes. First, the STING homologs found in the close unicellular relatives of metazoans such as choanoflagellates, which can induce autophagy upon activation^14^, and for which we chose *Monosiga brevicollis* (*M. brevicollis*) as a representative (Fig. 1A). Second, the STING homologs found in basal metazoan phyla, including species such as the sea anemone *Nematostella vectensis* (*N. vectensis*), or in protostomians including arthropods like *Drosophila melanogaster* (*D. melanogaster*) and mollusks like *Crassostrea gigas* (*C. gigas*). These STING proteins reportedly induce NF-κB signaling in response to activation^23,35–37^ (Fig. 1A). Third, STING from (jawed) vertebrates, such as *Danio rerio* (*D. rerio*), *Mus musculus* (*M. musculus*), or *Homo sapiens* (*H. sapiens*), which can induce type I IFN responses^1,16,19,38^ (Fig. 1A). A notable exception within the vertebrate group is the STING from amphibians such as *Xenopus tropicalis* (*X. tropicalis*), which cannot induce an IFN-response due to absence of a functional CTT^19^.

When the domain organization of STING from these species is considered, it is notable that all sequences comprise four N-terminal TMDs, with the exception of STING from *D. melanogaster* (DmSTING) which comprises 2-3 predicted TMDs^16,39^ and STING from *C. gigas* (CgSTING) which is devoid of TMD but rather harbors a TIR domain^15^ (Fig. 1B), suggesting that this protein may not localize to the ER. Finally, the CTT is generally absent from STING proteins of invertebrates, and presents the highest variability among vertebrates (Fig. 1B). CgSTING however presents a short putative CTT, highly divergent from vertebrate CTT (Fig. 1B and Supplementary Fig 1).

Excluding the CTT, the STING C-terminal domain, which contains the CDN-binding region (CBD), exhibits low overall amino acid sequence conservation (Fig. 1C), despite the CDN binding motif/pocket itself being highly conserved (Supplementary Fig 1). Prediction of the three-dimensional conformation of STING homologs across holozoan evolution shows a similar global architecture (Supplementary Fig 2). However, structural alignment of the most evolutionary distant STING homologs, namely the choanoflagellate MbSTING and the mouse MmSTING, showed poor overlap of the CBD (Fig. 1D). Unrooted evolutionary tree of STING-domains does not mirror the tree of life, consistent with varying pace of domain acquisition, modification, or loss during metazoan evolution (Fig. 1E).

Based on the conservation of the STING C-terminal domains, we thus selected representative STING genes from each of the three aforementioned groups to assess the conservation of STING-FADS2 interaction, and the role of STING in lipid metabolism regulation. Specifically, we used MbSTING for unicellular eukaryotes; NvSTING for basal metazoans; and MmSTING, XtSTING, and DrSTING for vertebrates.

### Identification of FADS2 homologs

Prior work established that MmSTING and HsSTING interact with FADS2^24^. FADS2 bears a Δ6-desaturase activity, which is essential for the desaturation of PUFAs to LC-PUFAs. Δ6-desaturase activities, essential for lipid metabolism have ancient origins^40,41^. The general structure of the prototypical FADS2 comprises 3 domains (Fig. 1F): an N-terminal cytochrome b5-like heme/steroid binding domain, which acts as the electron donor during desaturation of PUFAs, facilitating the transfer of electrons to the catalytic site; four TMDs that ensure anchoring of FADS2 to the ER and proper positioning of the catalytic site; the C-terminal fatty acid desaturase domain comprising histidine-rich motifs which form the catalytic site for desaturase activity^42^.

In order to analyze the conservation of the STING-FADS interaction, we investigated the presence of FADS2 homologs in the chosen organisms (Fig. 1A and Fig. 1F-H). Putative orthologs of mouse FADS2 have been identified in vertebrates *H. sapiens*, *D. rerio*, *X. tropicalis*^41^, but not in *N. vectensis* or *M. brevicollis*. Through analyses of the genome of the two latter species, we identified FADS-like genes (Fig. 1F-G) corresponding to distant homologs of vertebrate FADS1 and FADS2 (Fig. 1G-H). However, the global domain structure is similar in all these proteins, and the cytochrome b5-like domain is very well conserved (Supplementary Fig. 3). We thus modeled the 3D structure of FADS2 and FADS-like Δ6-desaturase enzymes to assess the conservation of the protein architecture and ascertain that the identified homologs in *N. vectensis* and *M. brevicollis* are bona fide FADS candidates with putative similar functions. Overlay of the models showed that the overall architecture is conserved (Fig. 1I and Supplementary Fig. 4).

Taken together, these analyses identify FADS2-related proteins (FADS) in all representative species from the phyla in which we aimed to characterize the STING-FADS interaction.

### STING from multiple species interact with their conspecific Δ6-desaturase enzymes

**Fig. 2:**
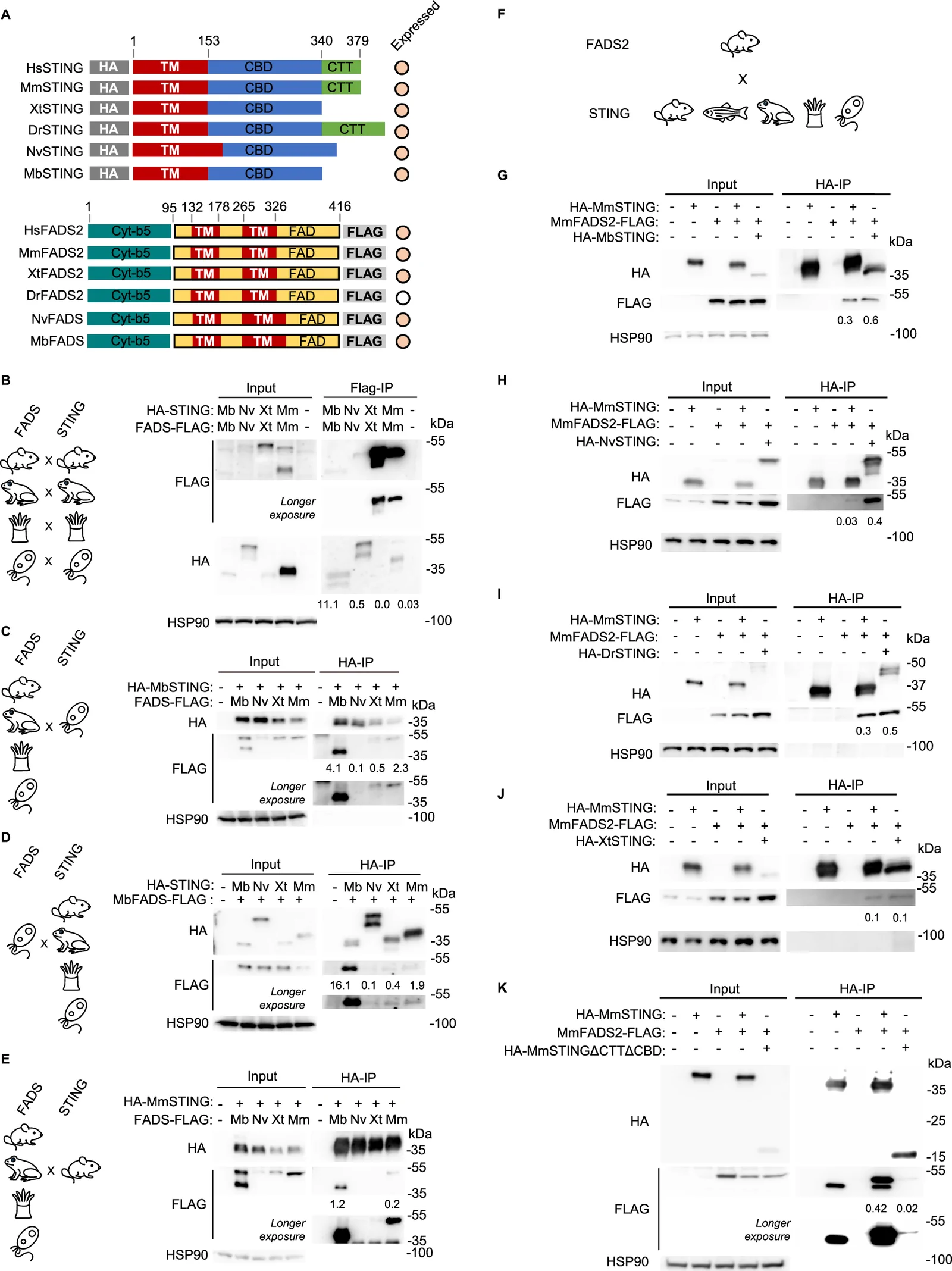
The interaction of STING with FADS is conserved. **A** Top panel: Schematics of HA-tagged STING constructs roughly aligned to HsSTING and their expression status in HEK 293T cells. Bottom panel: Schematics of FLAG-tagged FADS constructs roughly aligned to HsFADS2 and their expression status in HEK 293T cells indicated (orange: present, white: absent). **B** FLAG-immunoprecipitation was performed on whole-cell extracts (WCE) from HEK 293T cells transiently co-expressing HA-STING and FADS-FLAG of the indicated species or not (control). Inputs and eluates from the immunoprecipitation were analyzed by Western blot (WB). **C** As in **B**, except that HA-immunoprecipitation was performed on WCEs from HEK 293T cells transiently co-expressing HA-MbSTING with FADS-FLAG from the indicated species or not (control). **D** As in **C**, except that cells co-expressed MbFADS-FLAG with HA-Sting from the indicated species or not (control). **E** As in **C**, except that cells co-expressed HA-MmSTING with FADS-FLAG from the indicated species or not (control). **F** Scheme showing interaction studies of STING proteins from the indicated species with MmFADS2. **G** HA-immunoprecipitation was performed on WCEs from HEK 293T cells transiently co-expressing MmFADS2-FLAG, with HA-MbSTING, HA-MmSTING, or none (control). Inputs and eluates were analyzed by WB. **H** As in **G**, except that cells co-expressed MmFADS2-FLAG, with HA-NvSTING, HA-MmSTING, or none (control). **I** As in **G**, except that cells co-expressed MmFADS2-FLAG, HA-DrSTING, HA-MmSTING, or none (control). **J** As in **G**, except that cells co-expressed MmFADS2-FLAG, HA-XtSTING, HA-MmSTING, or none (control). **K** HA-immunoprecipitation was performed on WCEs from HEK 293T cells transiently co-expressing MmFADS2-FLAG or not with HA-MmSTING, HA-MmSTINGΔCTTΔCBD, or none (control). Inputs and eluates from the immunoprecipitation were analyzed by WB. WBs are representative of at least *n* = 3 independent experiments. Cells transfected with HA-STING or FADS2-FLAG only or none at all serve as negative controls. The ‘normalized binding index’ is indicated below the panel showing the co-immunoprecipitated protein.

To test if STING homologs can interact with their identified conspecific FADS-like Δ6-desaturase enzymes, codon-optimized STING and FADS2 or FADS-like sequences were hemagglutinin (HA)- and FLAG-tagged respectively (Fig. 2A), and inserted into retroviral vectors suitable for expression in mammalian cells. The FADS2 homologs cloned and tested for expression were from *M. brevicollis* (MbFADS), *N. vectensis* (NvFADS), *D. rerio* (DrFADS2), *X. tropicalis* (XtFADS2), and *M. musculus* (MmFADS2) (Fig. 2A).

We then tested the ability of MbSTING, NvSTING, and XtSTING to interact with conspecific FADS2 homologs. To this aim, HA-STING and FADS-FLAG constructs were co-expressed in HEK 293T cells prior to FLAG immunoprecipitation (IP), release of bound material by peptide elution, and analysis by WB. MmSTING and MmFADS2 were used as positive controls. Despite low expression levels, we found that MbFADS and NvFADS co-immunoprecipitated with MbSTING and NvSTING, respectively (Fig. 2B), with an enrichment higher than that of MmSTING with MmFADS2 (Fig. 2B). These observations indicated that an interaction between STING and a FADS-like protein arose prior to the emergence of metazoans, long before the duplication event that produced FADS1 and FADS2. In contrast, XtSTING did not co-immunoprecipitate with the expressed isoform of XtFADS2, suggesting that either the ability to interact with FADS2 was lost in *X. tropicalis* or that this ability is not harbored by this specific isoform.

We next assessed the ability of the STING and FADS2 homologs to interact with their putative counterparts from distant species. First, HA-STING from *M. brevicollis* was co-expressed with MbFADS, NvFADS, XtFADS2, or MmFADS2 prior to HA-IP and WB analyses. Co-immunoprecipitation of MbFADS, NvFADS, XtFADS2, and MmFADS2 was detected with MbSTING (Fig. 2C). We next co-expressed MmSTING, XtSTING, NvSTING, and MbSTING with MbFADS prior to HA-IP and WB analysis of FADS co-immunoprecipitation. We found that MbFADS co-immunoprecipitated with XtSTING and MmSTING and to a lesser extent with NvSTING (Fig. 2D). Next, when MmSTING was co-expressed with MbFADS, NvFADS, and XtFADS2, we found strong co-immunoprecipitation of MbFADS with MmSTING, while weak interaction of XtFADS2 was detected (Fig. 2E). Finally, we assessed whether STING from different species are capable of interacting with MmFADS2 (Fig. 2F). Co-expressing MbSTING, NvSTING, DrSTING, and XtSTING with MmFADS2 prior to HA-immunoprecipitation and WB analysis of FADS2 interaction showed that all tested homologs bear the capacity to interact with MmFADS2 (Fig. 2G-J).

Together, these data suggest that STING from multiple species bear the ability to interact with FADS-related proteins from distant species, although the strength of the detected interaction is variable. It is notable that the interaction of the FADS from a non-metazoan, *i.e*. MbFADS, was strongest with the mammalian MmSTING, in line with the idea that the ability to interact with FADS-like Δ6-desaturase enzymes is a conserved ancient function of STING, although the strength of the interactions did not correlate very well with evolutionary proximity.

### The CTT is dispensable and TMD sufficient for interaction of STING with FADS2

Our analyses show that STING from basal organisms lacking a CTT are able to interact with FADS2, suggesting that the CTT is likely dispensable for this interaction. Previous work^24^ has shown that the TMD of STING is essential for interaction with FADS2. To further investigate which domains are required for interaction between FADS2 and STING, we generated HA-tagged versions of STING homologs with unusual domain architecture, namely from the bacterium *Flavobacteriaceae sp*. (FsSTING, *cap13*) and mollusk *C. gigas* (CgSTING). In addition, we generated MmSTING and DrSTING mutants where the CTT is ablated (MmSTINGΔCTT and DrSTINGΔCTT, respectively), as well as a MmSTING mutant comprising the TMD alone (MmSTINGΔCTTΔCBD) (Supplementary Fig. 5A). The interaction of these homologs with MmFADS2 was subsequently tested in co-immunoprecipitation experiments.

The FsSTING (Supplementary Fig. 5B) and CgSTING (Supplementary Fig. 5C) were unable to interact with FADS2, confirming that ER anchoring is likely essential for interaction of STING with FADS2^24^. Indeed, FsSTING does have two TM domains, but owing to the absence of ER in bacteria, it can be speculated that the FsSTING localizes to other compartments, while CgSTING lacks TM domains altogether. In contrast, we found that ablation of the CTT led to enhanced interaction of MmSTINGΔCTT and DrSTINGΔCTT with MmFADS2, as compared to the WT counterparts (Supplementary Fig. 5D-E). Finally, we found that MmSTINGΔCTTΔCBD retained the ability to interact with MmFADS2 (Fig. 2K), and so did the STING homolog of *Drosophila melanogaster* (DmSTING) the CBD of which has very low sequence similarity with other STING homologs (Supplementary Fig. 1, Supplementary Fig. 5F).

Together these data (Supplementary Fig. 5G) show that the localization of STING at the ER membrane is essential for the interaction with FADS2, and that the TM region is both essential and sufficient for this interaction, while the CTT is dispensable. This further supports that the interaction of FADS2 and STING predated the emergence of the CTT in the STING of jawed vertebrates.

### STING homologs interact with *Mus musculus* FADS2 and are functional in murine cells

**Fig. 3:**
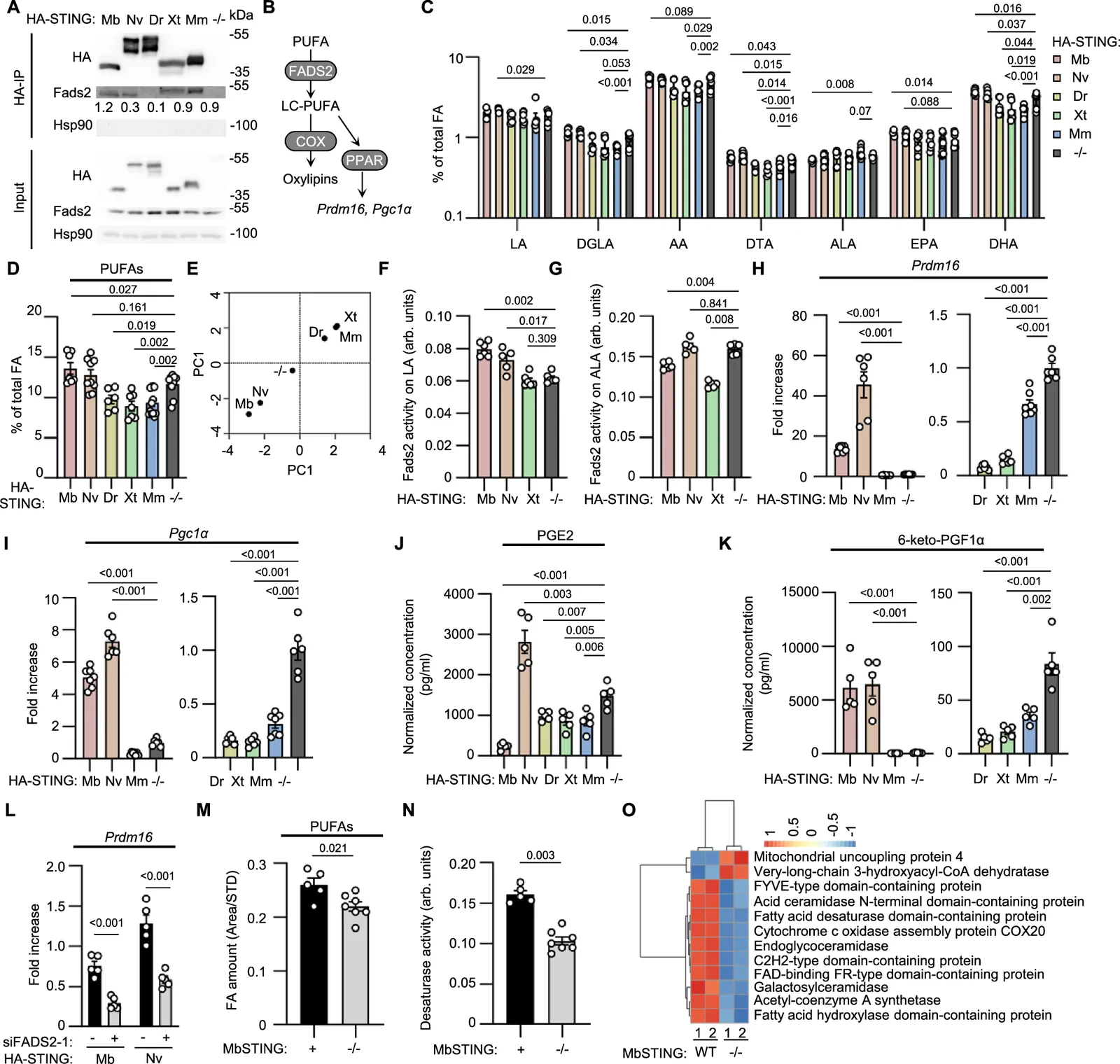
STING homologs differentially modulate PUFA pools. **A** HA-immunoprecipitation on whole-cell extracts (WCE) from MEF^*MbSTING*^, MEF^*NvSTING*^, MEF^*DrSTING*^, MEF^*XtSTING*^, MEF^*MmSting*^, and MEF^*Sting-/-*^. Inputs and eluates from the immunoprecipitation were analyzed by Western blot (WB). The ‘binding index’ is indicated. *n* = 3 independent experiments. **B** Scheme showing products downstream of FADS2 activity. COX: cyclooxygenases. **C** Mean relative quantities of the indicated PUFAs in the indicated cell lines, expressed as percentage of total fatty acids. *n* = 7(MEF^*MbSTING*^), *n* = 9(MEF^*NvSTING*^), *n* = 6(MEF^*DrSTING*^), *n* = 7(MEF^*XtSTING*^), *n* = 12(MEF^*MmSTING*^), *n* = 10(MEF^*Sting-/-*^) biological replicates. **D** Mean summation of FADS2 PUFA products measured in **C**, expressed as percentage of total fatty acids. Biological replicates are as in **C**. **E** PC score plot from principal component analysis performed on data from C. Biological replicates are as in **C**. **F** Mean ^13^C-GLA to ^13^C-LA ratio measured from the indicated cell lines, treated with ^13^C-LA. *n* = 6(MEF^*MbSTING*^, MEF^*XtSTING*^, MEF^*Sting-/-*^), *n* = 5(MEF^*NvSTING*^) biological replicates. *p*-values were determined by two-tailed Mann-Whitney test. **G** As in **F**, but showing ^13^C-stearidonic acid (SA) to ^13^C-ALA ratio in cells treated with ^13^C-ALA. *n* = 6(MEF^*MbSTING*^), *n* = 5(MEF^*NvSTING*^, MEF^*XtSTING*^, MEF^*Sting-/-*^) biological replicates. *p*-values were determined by two-tailed Mann-Whitney test. **H** *Prdm16* mRNA levels in the indicated cell lines, expressed as mean fold increase over control MEF^*Sting-/-*^. *n* = 7(MEF^*MbSTING*^, MEF^*MmSting*^), *n* = 6(MEF^*NvSTING*^, MEF^*DrSTING*^, MEF^*XtSTING*^, MEF^*Sting-/-*^) biological replicates. **I** As in **H**, but showing mean *Pgc1α* mRNA levels. Biological replicates are as in **H**. **J** Mean PGE2 concentrations in the cell lines as in **A**. *n* = 5 biological replicates**. K** As in **J**, except that 6-keto-PGF1α is measured. *n* = 5 biological replicates. **L** *Prdm16* mRNA levels in the cell lines as in **A**, following treatment with 10 nM siFADS2-1 or control siRNA, expressed as mean fold increase over mock transfected MEF^*NvSTING*^. *n* = 5 biological replicates. **M** Mean summation of PUFAs from WT *M. brevicollis* (*n* = 5) and *STING-/-* (*n* = 7). *n* biological replicates. **N** Mean ∆6-desaturase activity calculated from the data in **M**. Biological replicates as in **M**. **O** Heatmap of selected differentially expressed genes from *n* = 2 biological replicates from RNA-seq data (GSE174340). Error bars are ± standard error of the mean (SEM), *p*-values (shown above bars) were determined by two-tailed Student’s *t* test, and control group is *Sting-/-* except when otherwise stated.

In order to evaluate the capacity of the STING homologs from different taxonomic groups to regulate FADS2 activity, we next engineered mouse embryonic fibroblasts (MEF) knockout for *Sting* (MEF^*Sting-/-*^) stably expressing STING homologs from representative species *M. brevicollis*, *N. vectensis*, *D. rerio, X. tropicalis*, *M. musculus*: MEF^*MbSTING*^, MEF^*NvSTING*^, MEF^*DrSTING*^, MEF^*XtSTING*^, and MEF^*MmSting*^, respectively. Immunoprecipitation of HA-tagged STING homologs was conducted, coupled to analysis of endogenous FADS2 co-immunoprecipitation, showing that all tested homologs interacted with endogenous MmFADS2 (Fig. 3A). This not only supports that the interaction between murine FADS2 and STING homologs is well-conserved, but also suggests that the constructed heterologous cell lines are suitable for assessment of STING function.

We next tested the functionality of these heterologous murine cells expressing STING from distant species, by assessing their response to stimulation with 2’3’-cGAMP, followed by analysis of subcellular localization by immunofluorescence (Supplementary Fig. 6A-B), oligomerization by native electrophoresis (Supplementary Fig. 6C-D), and pathway activation by immunoblotting (Supplementary Fig. 6E-F). Immunofluorescence analyses showed that all the homologs are localized in the ER membrane in the absence of stimulation, as suggested by colocalization with calnexin (Supplementary Fig. 6A-B). Stimulation with 2’3’-cGAMP promoted their displacement from the ER, as suggested by decreased co-localization with calnexin (Supplementary Fig. 6A-B). Of note, the subcellular localization of endogenous MmFADS2 was also assessed, showing no change in localization following treatment with 2’3’-cGAMP (Supplementary Fig. 6A). When oligomerization in response to 2’3’-cGAMP was assessed, increase in tetramers and higher order oligomers were observed for NvSTING (Supplementary Fig. 6C), DrSTING, and MmSTING (Supplementary Fig. 6D), after 1–3 h of treatment with 2’3’-cGAMP. For XtSTING, dimer and tetramers were not clearly detectable, but an increase in oligomers was observed at 1–3 h post treatment (Supplementary Fig. 6D). In contrast, MbSTING showed an increase in tetramers 1–3 h post treatment, but no clear increase in higher order oligomers (Supplementary Fig. 6C). Finally, the induction of autophagy, the most ancient and well-conserved pathway downstream of STING activation^12,14^, was also detected showing that all homologs induced autophagy to levels similar to that observed in cells expressing MmSTING (Supplementary Fig. 6E-F).

Together these data show that the STING homologs expressed in MEF respond to stimulation with 2’3’-cGAMP, indicating that they are able to perform their conserved innate immunity-related functions, such as inflammatory response and autophagy induction.

### STING homologs regulate lipid metabolism differently across species

We next assessed whether STING homologs from distant species, which are capable of interacting with MmFADS2, also regulate its activity, focusing on MbSTING, NvSTING, DrSTING, and XtSTING. To this aim, we performed fatty acid profiling, analyzed transcriptional activation of metabolic genes downstream of LC-PUFA accumulation, and measured oxylipins in the heterologous cell systems that we generated (Fig. 3B).

We quantified the detectable PUFAs and LC-PUFAs by gas chromatography (GC) (Fig. 3C). This showed that levels of major omega-6 PUFAs *i.e*., dihomo-γ-linolenic acid (DGLA), arachidonic acid (AA), and docosatetraenoic acid (DTA) were significantly reduced in cells expressing vertebrate STING homologs (DrSTING, XtSTING, and MmSTING), while among the omega-3 PUFAs, only docosahexaenoic acid (DHA) showed a significant decrease (Fig. 3C). Levels of linoleic acid (LA) did not show significant differences. In contrast, invertebrate STING (MbSTING and NvSTING) expression led to increased levels of both omega-6 and omega-3 PUFAs (Fig. 3C).

The summation of the products of FADS2 detectable in our assay, reflecting its activity, showed that the expression of MmSTING decreased FADS2 activity as previously reported^24^ (Fig. 3D). Similarly, DrSTING and XtSTING also decreased the levels of FADS2 products (Fig. 3D). On the contrary, the levels of FADS2 products measured in cells expressing MbSTING and NvSTING not only increased as compared to cells expressing MmSTING, but also compared to *Sting-/-* cells (Fig. 3D). This distinct behavior of vertebrate and invertebrate STING homologs is illustrated by principal component analysis (PCA) with the relative quantities of PUFAs as variables (Fig. 3E). Cells expressing STING from representative species classified into two distinct clusters both markedly different from the *Sting-/-* cells: STING from the invertebrate or unicellular eukaryote species have an activating effect while vertebrate STING have an inhibiting effect on FADS2 activity (Fig. 3E). Regardless of this, the ratio of total omega-6 to omega-3 PUFAs remained constant for all tested homologs (Supplementary Fig. 7A). We confirmed that high FADS2 activity is indeed responsible for the increase in LC-PUFAs by directly accessing FADS2 activity on ^13^C-labeled LA and ALA by GC coupled to mass spectrometry (GC-MS). The ratio of FADS2 product to substrate indicated higher activity on ^13^C-labeled LA (Fig. 3F) but not ^13^C-labeled ALA (Fig. 3G) in MbSTING- and NvSTING-expressing cells compared to *Sting-/-* and the vertebrate STING control (XtSTING). The ratio of total LC-PUFAs generated downstream of FADS2 activity to FADS2 substrate, also gives a similar indication of FADS2 activity (Supplementary Fig. 7B-C). The increase in LC-PUFAs observed in case of the aforementioned STING homologs, is thus driven mainly by omega-6 PUFAs.

PUFAs strongly activate peroxisome proliferator-activated receptors (PPAR) transcription factors involved in regulation of glucose and lipid metabolism^27^, upregulating the expression of thermogenic regulators like PPARγ coactivator 1-alpha (*Pgc1α*) and PR domain-containing 16 (*Prdm16*)^43^. Our previous work^24^ has shown that one of the effects of STING ablation in mice is the upregulation of these genes due to accumulation of LC-PUFAs resulting from increased FADS2 activity. Therefore, we used their messenger ribonucleic acid (mRNA) levels in the heterologous cell lines as a read-out for MmFADS2 inhibition by the respective heterospecific STING (Fig. 3B). We found that all vertebrate STING homologs efficiently downregulated *Prdm16* (Fig. 3H) and *Pgc1α* expression (Fig. 3I). Surprisingly, mRNA levels of both *Prdm16* and *Pgc1α* were higher in MEF^*MbSTING*^ and MEF^*NvSTING*^ than in MEF^*Sting-/-*^ (Fig. 3H-I). Finally, we measured oxylipins that are metabolites downstream of AA generated by FADS2 activity (Fig. 3B), namely prostaglandin E2 (PGE2) and 6-keto-PGF1, which is a stable isoform of PGI2. We found that the expression of vertebrate STING led to a decrease of PGE2 (Fig. 3J) and 6-keto-PGF1 (Fig. 3K). In contrast, both oxylipins were increased in MEF^*NvSTING*^, while MEF^*MbSTING*^ displayed increased 6-keto-PGF1 and decreased PGE2 levels (Fig. 3J-K). Since increase of FADS2 activity by STING is unheard of, we verified that the increased activation of Prdm16 activity is dependent on FADS2 by using small interfering RNA (siRNA) targeting *Fads2* (Supplementary Fig. 7D). We found that silencing FADS2 led to decreased expression of *Prdm16* in MEF^*MbSting*^ and MEF^*NvSting*^ (Fig. 3L and Supplementary Fig. 7E). Altogether, these data indicate that while vertebrate STING homologs are able to decrease the levels of FADS2 products, homologs from invertebrate or unicellular eukaryote species promote an increase of FADS2-derived metabolites.

To validate our findings regarding invertebrate STING homologs in native systems, we performed lipid profiling and meta-analyses of available RNA sequencing datasets for corresponding *Sting-/-* organisms. Lipidomic analyses of wild-type (WT) and *Sting-/- M. brevicollis* showed a decrease in PUFAs (Fig. 3M), especially omega-6 PUFAs (Supplementary Fig. 7F) upon *Sting* ablation. Omega-3 LC-PUFAs were not detected in *M. brevicollis* indicating that ALA cannot be processed in this organism (Supplementary Fig. 7G). Indirect assessment of Δ6-desaturase activity from the gamma-linolenic acid (GLA) to LA ratio showed significant reduction in Δ6-desaturase activity in absence of STING (Fig. 3N), confirming our results from the heterologous system. Differential gene expression analysis of available RNA sequencing datasets^14^ showed downregulation of diverse lipid metabolism-associated genes in STING-deficient *M. brevicollis* (Fig. 3O), highlighting the importance of the mechanism we report in heterologous systems. Lipidomic analyses of WT control and *Sting-/- D. melanogaster* heads showed global lipid metabolic dysregulation manifested by increased saturated fats in the absence of STING (Supplementary Fig. 7H), likely due to an increase in ACC/FASN activity (Supplementary Fig. 7I), with which DmSTING is known to interact^32^.

Our interaction analyses showed that the CTT domain is dispensable while the TMD is necessary and sufficient for interaction of STING with FADS2. We assessed if the absence of CTT and CBD would alter FADS2 activity. To this aim, MEF^*Sting-/-*^ engineered to stably express HA-tagged MmSTING (MEF^*MmSting*^), DrSTING (MEF^*DrSTING*^), MmSTINGΔCTT (MEF^*MmSting∆CTT*^), MmSTINGΔCTTΔCBD (MEF^*MmSting∆CTT∆CBD*^), and DrSTINGΔCTT (MEF^*DrSTING∆CTT*^), were subjected to fatty acid profiling. We verified that the MmSTINGΔCTTΔCBD localizes in the ER membrane before proceeding to functional assays (Supplementary Fig. 8A). The level of the omega-6 PUFA DGLA was significantly reduced upon ablation of the CTT and CBD. The omega-3 PUFA EPA also showed reduction in the ΔCTT mutants, and ALA and DHA for the ΔCTTΔCBD mutant (Supplementary Fig. 8B). Summation of FADS2 products showed that ablation of the CTT or of the CBD causes a slight decrease in LC-PUFA products of FADS2, although the difference is not significant (Supplementary Fig. 8C). PCA (Supplementary Fig. 8D) and calculation of the omega-6 to omega-3 PUFA ratio (Supplementary Fig. 8E) showed no major difference between the WT and mutant STING pairs. Finally, gene expression analyses showed a significant downregulation of *Prdm16* and *Pgc1α* when STINGΔCTT is expressed as compared to WT STING (Supplementary Fig. 8F). No significant change in mRNA levels was observed upon ablation of the CBD of murine STING (Supplementary Fig. 8F). This suggests that the CTT is not just dispensable for the interaction with FADS2, but probably impedes the inhibitory effect of STING to some extent. The CBD on the other hand does not seem to have any effect on FADS2 activity.

In order to rule out the possibility of dose-dependent response due to varying expression levels of the STING homologs, we summarized the relative quantification of protein levels by WB and IF and compared them to the functional output of various orthogonal assays performed (Supplementary Table 1).

### STING activation disrupts lipid metabolism differently across species and mediates anti-viral function in the absence of interferons

Previous work^24^ indicated that STING activation by agonists disrupts the inhibition of FADS2 activity in murine cells, notably through the displacement of STING from the ER compartment. We questioned whether a similar release of STING-mediated inhibition of FADS2 activity can be witnessed for STING homologs after stimulation.

**Fig. 4:**
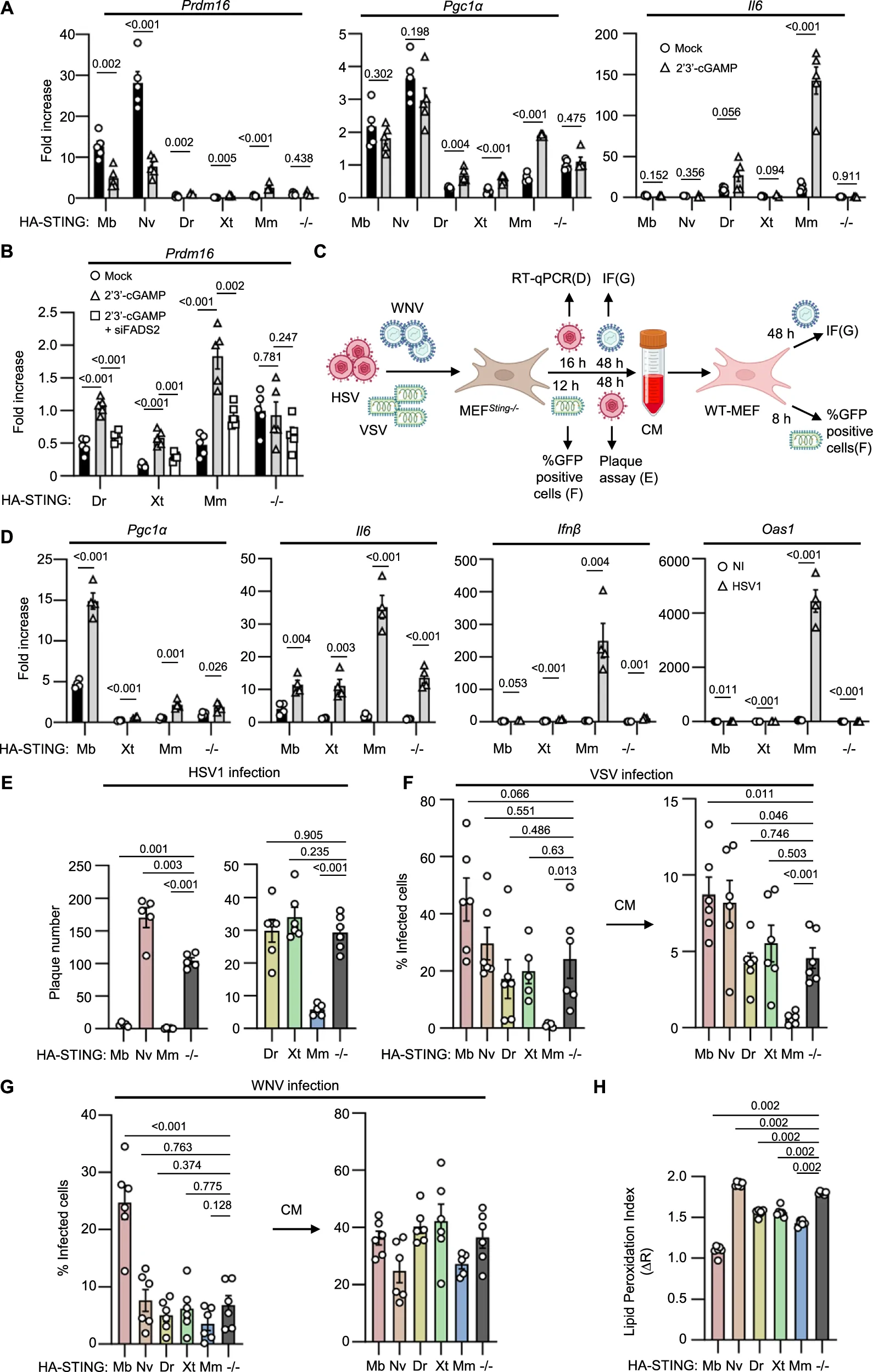
STING activation disrupts lipid metabolism differently across species and mediates anti-viral function in the absence of interferons. **A** *Pgc1α*, *Prdm16*, and *Il6* mRNA levels in MEF^*MbSTING*^, MEF^*NvSTING*^, MEF^*DrSTING*^, MEF^*XtSTING*^, MEF^*MmSting*^, and MEF^*Sting-/-*^, stimulated or not with 10 µM 2’3’-cGAMP, expressed as mean fold increase over unstimulated MEF^*Sting-/-*^. *n* = 5 biological replicates. **B** *Prdm16* mRNA levels in MEF^*DrSTING*^, MEF^*XtSTING*^, MEF^*MmSting*^, and MEF^*Sting-/*^, following treatment with 10 nM siFADS2-1 or control siRNA, followed by stimulation or not with 10 µM 2’3’-cGAMP, expressed as mean fold increase over mock transfected MEF^*Sting-/-*^. *n* = 5 biological replicates. **C** Simplified scheme for experiments **D**–G. Created in BioRender. Laguette, N. (2026) https://BioRender.com/7hp3yyg. **D** MEF^*MbSTING*^, MEF^*XtSTING*^, MEF^*MmSTING*^, and MEF^*Sting-/-*^ were infected with Herpes simplex virus 1 (HSV-1) prior to gene expression analysis and expressed as mean fold increase over non-infected (NI) ﻿MEF^*Sting-/-*^. *n* = 4 biological replicates. **E** MEF^*MbSTING*^, MEF^*NvSTING*^, MEF^*DrSTING*^, MEF^*XtSTING*^, MEF^*MmSting*^, and MEF^*Sting-/-*^ were infected with HSV-1 prior to viral plaque number analysis. Left panel: *n* = 5 biological replicates; Right panel: *n* = 6(MEF^*DrSTING*^, MEF^*XtSTING*^, MEF^*Sting-/-*^), *n* = 5(MEF^*MmSTING*^) biological replicates. **F** MEF^*MbSTING*^, MEF^*NvSTING*^, MEF^*DrSTING*^, MEF^*XtSTING*^, MEF^*MmSting*^, and MEF^*Sting-/-*^ were infected with vesicular stomatitis virus (VSV)-GFP (left) prior to collection of conditioned media (CM) and re-infection of WT-MEF (right). The mean percentage of infected cells are presented. *n* = 6(MEF^*MbSTING*^, MEF^*NvSTING*^, MEF^*DrSTING*^, MEF^*Sting-/-*^), *n* = 5(MEF^*XtSTING*^, MEF^*MmSting*^) biological replicates. **G** As in **F**, except that cells were infected with the West Nile virus (WNV). *n* = 6 biological replicates. **H** The mean lipid peroxidation index (∆R) of the cell lines as in **A**. *n* = 6 biological replicates. *p*-values were determined by two-tailed Mann-Whitney test. Error bars are ± standard error of the mean (SEM), *p*-values (shown above bars) were determined by two-tailed Student’s *t* test, and control group is *Sting-/-* except when otherwise stated.

We assessed the changes in STING homolog-mediated FADS2 activity modulation upon stimulation with dsDNA which stimulates production of 2’3’-cGAMP by cGAS. Measurement of PGE2 in this context showed no change upon dsDNA stimulation in MEF^*MbSting*^ and MEF^*NvSting*^, while an increase was observed for cells expressing vertebrate STING homologs (Supplementary Fig. 8G). Next, cells were challenged with 2’3’-cGAMP followed by analysis of the expression of *Prdm16* and *Pgc1α* as a surrogate for FADS2 activation. *Il6* expression was also monitored to assess the pro-inflammatory status of cells. We found that 2’3’-cGAMP induced *Il6* expression in cells expressing MEF^*DrSTING*^ and MEF^*MmSting*^ (Fig. 4A), consistent with their ability to trigger NF-kB activity (Supplementary Fig. 6F). In the cells expressing STING from vertebrate classes, 2’3’-cGAMP stimulation led to a significant increase in the expression of *Prdm16* and *Pgc1α*, suggesting alleviation of their inhibition of FADS2 activity (Fig. 4A). In contrast, no significant change to *Pgc1α* levels was observed for MEF^*MbSting*^ and MEF^*NvSting*^, while a reduction of *Prdm16* was observed for both homologs (Fig. 4A). We next verified whether the increased expression of metabolic response genes was dependent on FADS2 by using siRNAs to knockdown FADS2 prior to stimulation by 2’3’-cGAMP. We found that the increase in *Prdm16* levels was reverted to non-stimulated levels upon knockdown (Fig. 4B).

We next questioned whether infection by a dsDNA genome-harboring virus, such as the human herpes simplex virus 1 (HSV-1), known to promote cGAS-dependent 2’3’-cGAMP production, can induce similar alterations of FADS2 activity in the presence of STING homologs (Fig. 4C). To this aim, we used MEF^*MbSting*^, as representative of non-vertebrate models, and MEF^*XtSTING*^ and MEF^*MmSting*^ as representative of vertebrates. This choice of STING homologs also facilitated the isolation of IFN-independent processes. Gene expression analysis showed that MbSTING expression led to an increase of *Pgc1α*, *Il6*, and of the *Oas1* IFN response gene, and a tendency for increase of *Ifnβ* at 16 h post infection (Fig. 4D). Similarly, an increase of *Pgc1α*, *Il6*, *Ifnβ*, and *Oas1* was observed for XtSTING expressing cells (Fig. 4D). But for both homologs, the observed increase in inflammation-related gene expression (*Il6*, *Ifnβ*, and *Oasl1*) was not significantly different from what is observed in STING-deficient cells and below the response induced in MEF^*MbSting*^ (Fig. 4D). This suggests that activation of the tested STING homologs by endogenously produced 2’3’-cGAMP can induce a weak, but reproducible increase of metabolic gene activation alongside the prototypical STING associated inflammatory and type I IFN response.

Finally, we interrogated whether the STING homologs tested in this study may be able to promote the establishment of an antiviral status, despite low level of type I IFN response induction, solely through their ability to modulate lipid metabolism. To this aim, we first infected MEF^*MbSting*^ and MEF^*NvSting*^ with HSV-1 and measured viral replication through plaque assays (Fig. 4C). We found that, expression of MbSTING, but not of NvSTING, restricted viral replication (Fig. 4E). Cells expressing vertebrate STING homologs were also infected with HSV-1 prior to measurement of viral plaques. We found that both DrSTING and XtSTING failed to restrict HSV-1 plaque formation in contrast to MmSTING (Fig. 4E). Thus, the ability of DrSTING and XtSTING to inhibit FADS2 activity does not correlate with an ability to restrict HSV-1 infection. These data further support that the ability to regulate lipid metabolism does not correlate with restriction of DNA virus replication. We next assessed the ability of the negative-sense ssRNA vesicular stomatitis virus (VSV) and the positive-sense ssRNA West Nile virus (WNV) to infect and replicate in these heterologous cell lines. Cells were infected with VSV-GFP or with WNV prior to quantification of GFP or dsRNA levels respectively by flow cytometry or immunofluorescence to assess relative infection levels (Fig. 4C). WT-MEF cells were treated with conditioned media from infected cells followed by quantification of GFP or dsRNA to further assess the viral load produced by the different cell lines (Fig. 4C). Expression of DrSTING and XtSTING did not prevent infection by VSV and WNV effectively, as compared to MmSTING (Fig. 4F-G). Interestingly, MEF^*MbSting*^ showed significantly higher susceptibility to infection by both of these viruses compared to all vertebrate STING homologs, as well as to MEF^*Sting-/-*^ cells (Fig. 4F-G). Moreover, expression of MbSTING in cells also allowed higher viral replication by VSV as inferred from infection levels in cells treated with conditioned media (Fig. 4F). This higher infectibility by ssRNA viruses of MEF^*MbSting*^ cannot be explained by loss of IFN-inducing function of STING alone. Altogether, these data show that MbSTING fosters an antiviral state against dsDNA viruses that hinders infection by HSV-1 but not against ssRNA viruses, where the opposite effect is seen.

This indicates that the ability of STING to regulate lipid metabolism may bear a primordial anti-viral function against certain dsDNA viruses possibly through lipid-derived mediator regulation. Thus, we investigated the possibility of high lipid peroxidation being involved in this process. To mimic high reactive oxygen species (ROS) levels seen upon viral infection, we treated the cell lines with the peroxidation inducer cumene hydroperoxide (CHP), measuring the extent of membrane lipid peroxidation using BODIPY 581/591 before and after treatment. Predictably, the lipid peroxidation index (∆R), which indicates the change in membrane lipid peroxidation level in response to high ROS concentration, was largely influenced by the total PUFA content of the cell lines (Fig. 4H), except in case of MbSTING which had the lowest ∆R.

Altogether, our data show that the ability of STING to regulate lipid metabolism is highly conserved and certain distant homologs of the protein appear to bear an anti-viral function through the regulation of lipid-derived mediators in the absence of anti-viral gene expression. Even though the ∆R followed similar distribution as the total PUFA content across the cell lines, MbSTING showed low ∆R despite having high PUFA content. We infer that lower concentration of membrane lipid oxidation products (LOP) may provide increased protection against infection by certain dsDNA viruses like HSV-1 while at the same time facilitating infection by ssRNA viruses.

### Human STING haplotypes regulate FADS2 activity

**Fig. 5:**
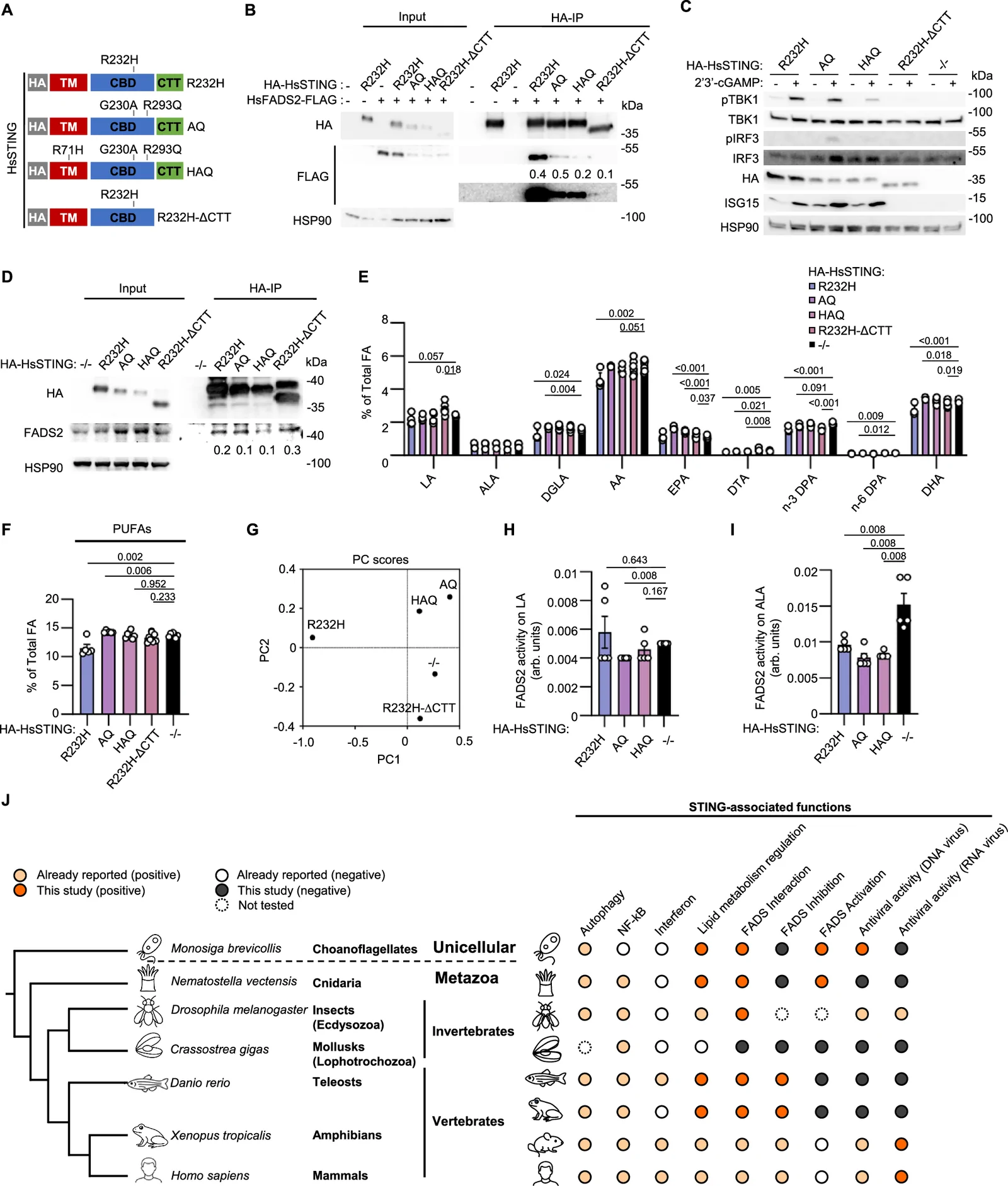
Human STING haplotypes regulate PUFA pools. **A** Schematics of HA-tagged constructs of HsSTING haplotypes and mutants. **B** HA-immunoprecipitation performed on whole-cell extracts (WCE) from HEK 293 T cells transiently co-expressing HsFADS2-FLAG with one of the indicated HA-tagged HsSTING haplotypes/mutants or none (control). Inputs and eluates from the immunoprecipitation were analyzed by Western blot (WB). **C** WCEs from THP-1^*HsSTING-R232H*^, THP-1^*HsSTING-AQ*^, THP-1^*HsSTING-HAQ*^, THP-1^*HsSTING-R232H-∆CTT*^, and THP-1^*STING-/-*^, stimulated or not with 10 µM 2’3’-cGAMP, were analyzed by WB. **D** HA-immunoprecipitation was performed on WCEs from THP-1^*STING-/-*^, THP-1^*HsSTING-R232H*^, THP-1^*HsSTING-AQ*^, THP-1^*HsSTING-HAQ*^, and THP-1^*HsSTING-R232H-∆CTT*^. Inputs and eluates from the immunoprecipitation were analyzed by Western blot (WB). **E** Mean relative quantities of the indicated PUFAs in the cell lines as in C, expressed as percentage of total fatty acids. *n* = 5(THP-1^*HsSTING-R232H*^), *n* = 6(THP-1^*HsSTING-AQ*^), *n* = 8(THP-1^*HsSTING-HAQ*^, THP-1^*HsSTING-R232H-∆CTT*^), *n* = 7(THP-1^*STING-/-*^,) biological replicates. **F** Mean summation of FADS2 PUFA products measured in **E**, expressed as percentage of total fatty acids. Biological replicates are as in **E**. **G** PC score plot from principal component analysis performed using the data from **E**. Biological replicates are as in **E**. **H** Mean ^13^C-GLA to ^13^C-LA ratio measured from THP-1^*HsSTING-R232H*^, THP-1^*HsSTING-AQ*^, THP-1^*HsSTING-HAQ*^, THP-1^*HsSTING-R232H-∆CTT*^, and THP-1^*STING-/-*^ after treatment with ^13^C-LA. *n* = 5 biological replicates. *p*-values were determined by two-tailed Mann-Whitney test. **I** As in **H**, but showing mean ^13^C-stearidonic acid (SA) to ^13^C-ALA ratio in cells treated with ^13^C-ALA. *n* = 5 biological replicates. *p*-values were determined by two-tailed Mann-Whitney test. **J** Scheme summarizing the data obtained in the study. STING-related functions are indicated with their status at the beginning of the study (‘Already reported’) and data we report (‘This study’). ‘positive’ indicates a process regulated by STING, ‘negative’ indicates a process not regulated by STING. WBs are representative of at least 3 independent experiments. The ‘normalized binding index’ (for immunoprecipitation experiments in transient expression cell lines i.e. **B**) or the ‘binding index’ (for immunoprecipitation experiments in stable expression cell lines i.e. **D**) is indicated below the panel showing the co-immunoprecipitated protein. Error bars are ± standard error of the mean (SEM), *p*-values (shown above bars) were determined by two-tailed Student’s *t* test, and control group is *STING-/-* except when otherwise stated.

To investigate whether different human STING haplotypes bind FADS2, we generated codon-optimized HsFADS2-FLAG sequence and HA-HsSTING sequences for STING-R232H, STING-AQ (G230A, R293Q), and STING-HAQ (R71H, G230A, R293Q) haplotypes, along with a STING-R232H-∆CTT mutant (∆342-379) (Fig. 5A). The AQ and HAQ variants were generated in an R232 background. These sequences were co-expressed into HEK 293T cells, prior to HA-immunoprecipitation. The bound material released by peptide elution was then analyzed by WB (Fig. 5B). Interestingly, we observed that co-expression of FADS2 with HA-HsSTING-R232H led to a stabilization of the total FADS2 protein levels in the input material (Fig. 5B). We calculated the ratio of FADS2 to STING in the eluate, followed by normalization to FADS2 levels in the input to account for variance in the level of FADS2 and STING haplotype/mutant expression. Although all the tested haplotypes/mutant of HsSTING bound HsFADS2, the HAQ haplotype showed comparatively weaker interaction (Fig. 5B). We also confirmed that like in other vertebrates, the CTT of HsSTING is dispensable for FADS2 binding (Fig. 5B).

Next, we checked if the interaction takes place in THP-1 human myeloid cells. THP-1^*STING-/-*^ cells were engineered to stably express the *Hs*STING haplotypes, allowing the generation of THP-1^*HsSTING-R232H*^, THP-1^*HsSTING-AQ*^, THP-1^*HsSTING-HAQ*^, and THP-1^*HsSTING-R232H-∆CTT*^ cell lines. We first tested the functionality of these alleles in THP-1 cells by performing 2’3’-cGAMP transfection, followed by assessment of the phosphorylation of TBK1 and IRF3 and levels of the ISG15 IFN response gene. We found that all haplotypes efficiently allowed activation of TBK1 (Fig. 5C). HsSTING-R232H and HsSTING-HAQ presented a reduced ability to induce IRF3 phosphorylation as compared to HsSTING-AQ, although all 3 haplotypes could induce ISG15 upregulation (Fig. 5C). HsSTING-R232H-∆CTT on the other hand lost its ability to induce activation of downstream effectors (Fig. 5C).

Next, we used these cell lines to perform HA-IP and found that all haplotypes interacted with FADS2, with a weaker interaction for the HAQ haplotype (Fig. 5D), similar to what was observed in HEK 293T cells (Fig. 5B). This differential binding of FADS2 by AQ and HAQ haplotypes possibly arises from the single point mutation R71H, which is known to increase the polar solvent exposure of STING^33,44^. Interestingly, this residue is part of the highly critical TM2–TM3 linker region of STING, which interacts with fatty acyl–CoA^33^, cholesterol^45^ and phospholipids^46–48^. Finally, we also confirm that the CTT is dispensable for interaction with FADS2 (Fig. 5D).

We next performed fatty acid profiling and quantification of mRNA levels of genes downstream of perturbation in fatty acid levels, in order to investigate how the human STING haplotypes regulate fatty acid metabolism. Detectable PUFAs were quantified by GC. Levels of major omega-6 PUFAs LA, DGLA, AA, n-6 DPA, and DTA, were all significantly reduced in R232H compared to THP-1^*STING-/-*^, while only n-3 DPA and DHA were found to be significantly reduced among omega-3 PUFAs (Fig. 5E). The other haplotypes and the R232H-∆CTT mutant, did not show much change in PUFA levels. (Fig. 5E). The summation of PUFA levels downstream of FADS2 activity, reflecting its enzymatic activity, showed that only cells expressing the R232H haplotype had significantly lower LC-PUFAs, while that of AQ was marginally higher than THP-1^*STING-/-*^ (Fig. 5F). Other than the AQ, where a minute decrease was visible, none of the haplotypes were able to significantly reduce the omega-6 to omega-3 PUFAs ratio, while the R232H-∆CTT mutant slightly increased the ratio compared to THP-1^*STING-/-*^ (Supplementary Fig 9A). PCA also illustrated that the R232H presented a distinct behavior, as compared to other haplotypes (Fig. 4G). The effect might be in part due to the higher expression level of the R232H haplotype compared to others. Although higher intrinsic stability of the variant has not been reported, it might arise from the stronger interaction with FADS2. By directly accessing FADS2 activity in the HsSTING haplotype expressing cell lines, we concluded that lower FADS2 activity as compared to THP-1^*STING-/-*^ is indeed responsible for the decrease in LC-PUFAs. The FADS2 product to substrate ratio indicated significantly lower activity on ^13^C-ALA (Fig. 5I), but not on ^13^C- LA (Fig. 5H), indicating that the effect is driven mainly by omega-3 PUFAs. These data show that not only do all human STING haplotypes possess the capacity to regulate PUFA metabolism, but also highlight that the CTT might play a role in this process.

To investigate the possible role of this function in anti-viral response, we assessed the lipid peroxidation potential of the cell lines. We calculated their ∆R in a similar manner as for the MEF cell lines in the previous section. The ∆R was consistent among the different haplotypes and mutants, being only marginally higher in case of HAQ and R232H-∆CTT as compared to *STING-/-* (Supplementary Fig. 9B). Thus, it is unlikely to play a major role in anti-viral defense.

Altogether our results point towards the conservation of the ability of STING to regulate PUFA metabolism throughout evolution, which therefore represents a primordial function of this protein (Fig. 5J).

## Discussion

In this study, we conducted a thorough analysis of the evolutionary conservation of the interaction of STING with FADS-like proteins, showing that the ability of STING to interact with FADS-like enzymes and to regulate lipid metabolism arose early in evolution, before the emergence of metazoans. Since prokaryotes also possess STING homologs^15^ along with multiple FADS-like desaturase enzymes^49–51^, the interaction between the two proteins might also exist in prokaryotic cell membrane in an archaic form. Consistent with this ancient role, the CTT domain of STING, which is restricted to vertebrates, is dispensable for the interaction with FADS2 and the regulation of FADS2 activity. While lipid metabolism is virtually present in all living cells, activities of FADS-like enzymes had not yet been reported in all species. We here identify FADS2 homologs in unicellular eukaryotes that contain domains similar to those present in vertebrate FADS2, and possess the ability to interact with STING. While the direct activity of these enzymes should be measured to assert their role in PUFA processing, our data from *M. brevicollis* support previous observations that although some FADS-domain containing enzymes in basal metazoans and unicellular eukaryotes can use PUFAs as substrates^52–54^, others have diverged to perform different functions.

The putative FADS2 homolog we identified in *D. melanogaster* i.e. cytochrome b5-related protein (Uniprot: P19967) was shown to interact with DmSTING in larval fat bodies^32^, although such proteins are highly divergent in arthropods, suggesting that they have evolved to perform unrelated functions in this group. Accordingly, the cytochrome b5-related protein of *D. melanogaster* displays low similarity to mammalian FADS2 and has been suggested to localize in the mitochondria and perform divergent enzymatic activities^55^. DmSTING possibly modulates those other metabolic activities which may crosstalk with lipid metabolism. The *D. melanogaster* ∆9-desaturase enzyme Desat1 (murine SCD1 ortholog) involved in monounsaturated fatty acids (MUFA) synthesis, another distant homolog of murine FADS2, also interacts with DmSTING^32^. This is of particular interest because drosophila does not possess the enzymes responsible for downstream PUFA processing and thus it highlights the apical role of STING-mediated regulation of lipid synthesis.

Duplication events are believed to have given rise to FADS1 and FADS2 in early vertebrates^41^. These desaturases present distinct desaturase activities, with FADS1 bearing Δ5 activity while FADS2 bearing Δ6 activity, and both enzymes cooperating to generate LC-PUFAs^51^. Invertebrates rather mostly rely on multifunctional desaturases that can perform both Δ5 and Δ6 activities^41,56^. Interestingly, zebrafish has secondarily lost its FADS1 and relies on a multifunctional FADS2 enzyme^57^. These differences in PUFA management between vertebrates and invertebrates may account for differential outputs of STING-dependent regulation of FADS-like enzymes. Indeed, we found that STING homologs regulate lipids differently across species from different lineages. In vertebrates, STING efficiently inhibits the desaturation of PUFAs into LC-PUFAs, likely reflecting decreased FADS2 activity. In contrast, we found that STING from unicellular eukaryotes and basal metazoans promote an enhancement of FADS2 products, and subsequently, higher activity of tested metabolic transcription factors. This suggests that these STING homologs regulate FADS2 activity differently, or that they may recruit other metabolic enzymes, ultimately modifying metabolic outputs. In this line, recent proteomic studies revealed a diverse array of enzymes involved in lipid metabolism^58^ in the proximitome of STING. The interaction of STING with these other lipid metabolic enzymes and its evolution remains to be investigated.

Our study highlights an ancient role of STING as a lipid metabolism regulator, prior to the emergence of the IFN system. This raises questions regarding how and why this protein involved in metabolism has been co-opted by the IFN-based innate immune system, and whether this dual function of STING conferred an evolutionary advantage. Importantly, lipids generated by FADS2 activity can be processed into signaling lipids, also called oxylipins, that bear signaling properties and can regulate inflammatory processes^59,60^. Therefore, by coordinating PUFA processing concertedly with type I IFN responses, as well as other innate immune pathways, activated STING may allow for better fine-tuning of signaling output. In this line, the prevalence of STING haplotypes deficient for type I IFN signaling in some human populations^33^, without reported immune dysfunction^61^, suggests that the lipid metabolism-regulating function of STING is sufficient for homeostasis. Deeper assessment of the molecular mechanisms through which oxylipins resulting from the concerted action of FADS2 and other enzymes may affect viral infections is therefore warranted to understand whether the function of STING in lipid metabolism regulation may also serve an antiviral function. Additionally, studies on Hepatitis C virus (HCV) have suggested that disruption of STING-mediated regulation of PUFA metabolism may also contribute to antiviral defense by inhibition of the viral replication machinery of positive-stranded ssRNA viruses that rely on ER-derived membranes. This is achieved by LOP-mediated disruption of membranes, a process that is highly FADS2-dependent and parallels ferroptosis^62,63^. However, we did not observe any such anti-viral effect on the ssRNA viruses tested in our heterologous system, namely VSV and WNV, while the MbSTING offered increases resistance against HSV-1. We suspect that low lipid peroxidation potential in these cells might provide a protective effect against dsDNA virus infection as high oxidative stress and lipid peroxidation is beneficial for HSV-1 replication^64^. The same phenomenon is likely involved in making cells more susceptible to ssRNA virus infection as LOP-mediated disruption of membranes restricts ssRNA virus^63^, an effect that is diminished in MbSTING expressing cells due to low peroxidation levels.

STING is a major therapeutic target, for which a plethora of agonists has been developed with the aim of boosting inflammatory responses in immunosuppressed contexts such as cancer^65–67^. Our study sheds light onto the way in which STING and FADS2 interact, the conservation of those interactions, as well as the potential metabolic consequences of interfering with STING activity. This work therefore opens perspectives for the development of therapeutics that would allow dissociating STING-associated inflammatory pathways from metabolic side-effects, or to the contrary contend with STING-dependent lipid metabolism without causing type I IFN responses activation. This is of particular importance when considering the previously established link between STING and ferroptosis^68–70^, which has been described to rely on FADS2 activity and lipid metabolites^63,71,72^. Investigating this interconnection may provide a better understanding of STING-dependent cell death processes in health and diseases.

## Methods

### Cells and cell cultures

HEK 293T (ATCC, CRL-3216) and MEF^*Sting-/-*^ (gift from Soren R. Paludan, Aarhus University, Aarhus) were maintained in DMEM (Gibco, 11960-044) supplemented with 10% Fetal Bovine Serum (FBS; Sigma, F7524-500ML), 100 µg/ml Penicillin-Streptomycin (Pen-Strep; Gibco, 10378016), and 1% L-Glutamine (Gibco, 25030081). THP1-Dual KO-STING (Invivogen, thpd-kostg) were maintained in RPMI 1640 w/ stable L-Glutamine (Clearline, 509088) supplemented with 10% FBS, 1% Penicillin-Streptomycin, and 100 µg/ml Normocin (Invivogen, ant-nr-05) at 5 × 10^5^ cells/ml. All cells were maintained at a temperature of 37 °C under 5% CO_2_. MEF^*Sting-/-*^ expressing N-terminal HA-tagged STING homologs/mutants (MbSTING, NvSTING, DrSTING, DrSTING-ΔCTT, XtSTING, MmSTING, and MmSTING-ΔCTT) and THP1-Dual KO-STING expressing N-terminal HA-tagged HsSTING haplotypes/mutants (HsSTING-R232H, HsSTING-AQ, HsSTING-HAQ, and HsSTING-R232H-ΔCTT) were generated by transduction of MEF^*Sting-/-*^ or THP1-Dual KO-STING with retroviral particles packaging the corresponding pOZ-HA-STING or the pOZ-STING-HA constructs generated for each STING homolog/mutant/haplotype, followed by selection with 2 µg/ml puromycin.

### Plasmid constructs

The pOZ-N-HA-Puro and pOZ-HA-C-Puro expression vectors used for expression of STING homologs and pOZ-FLAG-C-Il2 expression vector used for expression of FADS2 and FADS-like enzymes were obtained from Bijan Sobhian, IGH, Montpellier. The codon optimized sequences corresponding to STING homologs (FsSTING, MbSTING, NvSTING, CgSTING, DrSTING, XtSTING, and MmSTING), the FADS2 homologs (MbFADS, NvFADS, DrFADS2, XtFADS2, and MmFADS2) were purchased from Eurofins Genomics as synthetic genes. The DrSTING-ΔCTT, and MmSTING-ΔCTT were generated in-house by deleting the C-terminal tail of the DrSTING and MmSTING using PCR cloning. The sequence for HsSTING-R232H was also obtained from Bijan Sobhian, IGH, Montpellier and the AQ, HAQ, and ΔCTT of HsSTING were generated in-house from this sequence using PCR cloning and site-directed mutagenesis. The STING and FADS homolog sequences are listed in Supplementary Table 2.

### RNA extraction and quantitative real-time PCR

Total RNA was extracted using TRIzol reagent (Invitrogen, 15596026) from cell pellets. RNA was quantified with a NanoDrop spectrophotometer and 1 µg RNA was reverse transcribed using OneScript RT and gDNAOut Mix (Ozyme, OZYA012-100). Gene expression analysis of specific genes was performed by using through real-time PCR using ONEGreen FAST qPCR Premix (Ozyme, OZYA008-1000) and appropriate primers in a Biorad CFX Opus 384. Reactions were performed in duplicates and relative quantification of target cDNA was done using the 2^-ΔΔCT^ method by normalizing to heat shock protein 90 (*Hsp90*) cDNA. Graphs were plotted and statistical analysis was done using Graphpad Prism. The primer sequences (IDT) used for RT-qPCR analyses are listed in Supplementary Table 3.

### Cell seeding, transfection, and treatments

For immunoprecipitation (IP) experiments with transiently overexpressed proteins, 4 × 10^6^ HEK 293T cells were seeded per 150 mm culture dish 24 hrs before transfection. For each dish, 12.5 µg of pOZ construct expressing each of the target protein was transfected using CaCl_2_. The transfection medium was replaced with fresh medium after 8 hrs and incubated for 48 hrs before harvest by scraping. For IP experiments with stably expressed proteins, 2 × 10^6^ MEF^*Sting-/-*^ cells expressing the indicated STING homolog was plated per 150 mm culture dish and incubated for 24 h prior to harvest by scraping.

2 × 10^5^ MEF cells were seeded per well of 6-well plates 24 h before 2’3’-cGAMP treatment. Cells were treated with 4 μl Lipofectamine 2000 reagent (Invitrogen, 11668019) with or without 10 µM 2’3’-cGAMP (Invivogen, tlrl-nacga23-1) in 1 ml total volume of Opti-MEM Reduced Serum Medium (Gibco, 51985) per well. For gene expression analyses by RT-qPCR, cells were harvested 9 h following 2’3’-cGAMP transfection. For protein detection and quantification by western blot, cells were harvested 6 hrs following 2’3’-cGAMP transfection. In western blot analyses where treatment with chloroquine was necessary to enhance LC3B-lipidation, cells were pre-treated or not with 20 µM chloroquine (Sigma, C6628) for 3 h before 2’3’-cGAMP transfection in the presence or absence of 20 µM chloroquine. Analysis of STING oligomerization with native gel was done following 1 h or 3 h of treatment with 2’3’-cGAMP. For immunofluorescence experiments, cells were seeded in poly-D-lysine coated coverslips followed by transfection of 2’3’-cGAMP for 3 hrs.

For transfection with FADS2 siRNA, 10 nM siRNA duplex (IDT) complexed with INTERFERin transfection reagent (Polyplus, 101000028) in Opti-MEM Reduced Serum Medium was used according to the manufacturer’s instructions to reverse transfect 0.7 × 10^6^ MEF^*Sting-/-*^ cells expressing the indicated STING homolog plated per well of 6-well plate in 2 ml of antibiotic-free complete DMEM and incubated for 24 h. A second round of transfection with siRNA was performed following the same procedure and gene and protein expression analysis was performed 48 hrs later. The siRNA sequences used are listed in Supplementary Table 3.

Eighty-nucleotide long dsDNA probes (as described in ref. ^73^) used for the stimulation of STING were purchased as synthetic ssDNA from IDT. Sense and anti-sense ssDNA probes were annealed in a reaction combining equal quantities of the complementary ssDNA probes in an annealing buffer (60 mM NaCl, 10 mM Tris-HCl, and 200 μM EDTA), using a temperature gradient from 95 °C to 4 °C. The integrity of the dsDNA probes were then assessed using 10% acrylamide gel electrophoresis in Tris-Borate-EDTA buffer and visualized using ethidium bromide or SYBR safe under UV light. 2 × 10^5^ MEF cells were seeded per well of 6-well plates 24 hrs before transfection. 2 µg of dsDNA probes were transfected per well using 4 ul of JetPrime transfection reagent (Polyplus, 101000001), in 2 ml of complete DMEM. For ELISA, cells were harvested 24 hrs after transfection. The sequence of the dsDNA probe is given in Supplementary Table 3.

For plaque quantification assay after HSV-KOS64 infection or assessment of lipid peroxidation, 5 × 10^4^ MEF cells were seeded per well of 24-well plates 24 h prior to infection or treatment. For VSV and WNV infection experiments, 1 × 10^4^ MEF cells were plated per well of 96-well plate.

For treatment of cells with ^13^C_18_ labelled PUFAs, ^13^C_18_-LA (MCE, HY-N0729S2) and ^13^C_18_-ALA (MCE, HY-N0728S3) were complexed with 1% FA-free BSA (4 mole FA/mole BSA) (Sigma-Aldrich, A8806) in Opti-MEM. MEF and THP-1 cell lines were treated with either ^13^C_18_-LA or ^13^C_18_-ALA at 50 μM in Opti-MEM (containing 25 mM HEPES buffer) for 16 h at 37 °C.

### Western blot analyses

Protein samples for denaturing western blots were prepared by lysing the cell pellet in 5 times the packed cell volume of TENTG-150 buffer (20 mM Tris-HCl pH 7.4, 0.5 mM EDTA, 150 mM NaCl, 10 mM KCl, 0.5% Triton X-100, 1.5 mM MgCl₂, and 10% glycerol, supplemented with 10 mM β-mercaptoethanol, 0.5 mM PMSF, and 1× phosphatase inhibitor) for 30 min at 4 °C in a rotary mixer. Lysate was then clarified by centrifugation at 14,000 × g for 30 min at 4 °C, and the resulting supernatant was stored at −80 °C. 20 µg of solubilized protein was mixed with Laemmli buffer and heated at 95 °C for 5 mins before separation by SDS-PAGE on a 10%, 12%, or 10-20% Tris-glycine gel (Invitrogen Novex, XP00105BOX/ XP00125BOX/ XP10202BOX) and transferred to a nitrocellulose membrane (Biorad, 1704159) using the Biorad Trans-Blot Turbo semi-dry transfer system.

Protein samples for non-denaturing (native) western blots were prepared by lysing the cell pellet in native lysis buffer (25 mM NaCl, 20 mM HEPES pH 7.0, 10% glycerol, 1% DDM, supplemented with 1x protease inhibitor cocktail)^74^ following the same procedure as mentioned above. Samples were prepared by adding the lysate to NativePAGE sample buffer (Life Technologies, BN2008) and NativePAGE 5% G-250 Sample Additive (Life Technologies, BN2008). Samples were then run on a NativePAGE 3–12% Bis-Tris gel (Invitrogen, BN1001BOX) using the NativePAGE Running Buffer Kit (Invitrogen, BN2007) and transferred to a PVDF membrane (Biorad, 1704156) using the Biorad Trans-Blot Turbo semi-dry transfer system.

The western blot membranes were blocked with 5% milk in phosphate buffered saline (PBS) with 0.1% Tween (PBST) for 30 mins at RT and probed with the indicated primary antibodies (Supplementary Table 4). Membranes were then incubated in horseradish peroxidase (HRP)-conjugated secondary antibodies (Supplementary Table 4) diluted in 5% milk. Immunoreactive proteins were visualized by chemiluminescent detection using SuperSignal West Pico Plus (Thermo Scientific, 34577) or Femto (Thermo Scientific, 34094) reagents.

### Immunofluorescence

Cells attached to coverslips were fixed with cold methanol (Carlo Erba, 525101) for 5 mins at −20 °C and blocked with 5% bovine serum albumin (BSA; Sigma, A2153-100G) in PBST for 30 mins at RT. Coverslips were incubated with the indicated primary antibodies (Supplementary Table 4) diluted in the blocking solution at 37 °C for 45 min. Next, coverslips were incubated at 37 °C for 45 min with the corresponding secondary antibodies (Supplementary Table 4). Antibodies were diluted in the blocking solution. Cells were then stained with DAPI (Invitrogen, D1306) by incubating in 1 µg/ml DAPI diluted in PBS for 2 min at RT. Finally, coverslips were mounted in Vectashield antifade mounting medium (Vectorlabs, H-1900) on StarFrost slides (Knittel Glass). Images were acquired on the Zeiss confocal LSM880 Airyscan microscope using the Zeiss Zen software. Images analyzed using the FIJI image processing package. Protein colocalization analysis was performed with Cell Profiler. Graphs were plotted and statistical analysis was done using GraphPad Prism or R.

### Immunoprecipitation

Cells were lysed in 5 times the packed cell volume of TENTG-150 buffer and the input of the immunoprecipitation experiments were prepared as mentioned in the ‘Western blot analyses’ section. The HA/FLAG-tagged FADS/STING was immunoprecipitated using either agarose beads conjugated to anti-HA antibody (Santa Cruz Biotechnology, sc-7392 AC) or EZview Red anti-FLAG M2 beads (Sigma-Aldrich, F2426). Lysate containing approx. 4–7 mg of total protein was incubated with 10 µl packed bead volume of appropriate beads for 4 hrs at 4 °C in a rotary mixer. The immunoprecipitated proteins along with their binding partners were eluted from the beads using an elution buffer containing either 1 mg/ml HA-peptide (Roche, 11666975001) or 200 ng/µl 3xFLAG peptide (Sigma-Aldrich, F4799) in TENTG-150. The eluate was then boiled in Laemmli buffer and analyzed by western blot.

The ‘normalized binding index’ for immunoprecipitation experiments in transient expression cell lines was calculated by normalizing the ratio between band intensities of the immunoprecipitated prey and bait to that of the prey input. The ‘binding index’ for immunoprecipitation experiments in stable expression cell lines is the ratio between band intensities of the immunoprecipitated prey and bait.

### HSV-KOS64 infection

HSV-KOS64 molecular clone expressing a GFP was a gift from Soren Paludan, Aarhus University, Aarhus. MEF expressing the indicated STING homologs were infected with an MOI dilution series of HSV KOS-64-GFP in DMEM for 90 min and then the infection medium was replaced with DMEM containing 2% human serum (Sigma-Aldrich, H6914). Sixteen hours post-infection, the medium was changed to DMEM supplemented with 10% FBS and incubated for another 32 h. Cells were then fixed with 4% paraformaldehyde (PFA; Thermo Scientific, 28908) for 10 min at RT and stained with crystal violet (Sigma-Aldrich, C0775). The number of plaques per condition were then counted. Graphs were plotted and statistical analysis was done using Graphpad Prism.

### VSV infection

VSV-GFP was a gift from Dr. Sebastien Pfeffer (IBMC, France). VSV-GFP was amplified on BHK21 cells, aliquoted and frozen at −80 °C. VSV-GFP was diluted in serum-free DMEM to the desired MOI. MEF cells were infected with VSV-GFP for 1 h in DMEM, washed with PBS and media replaced with complete DMEM and incubated for 12 h. Cells were fixed using 4% PFA for 20 min at room temperature and nuclei stained with DAPI. The percentage of infected cells (i.e., GFP-positive cells) was determined using ImageXpress Pico (Molecular Devices). Conditioned media from infected cells was collected and WT-MEF were incubated in it for 8 hrs. The same procedure as above was used to determine the percentage of cells infected by the conditioned media.

### WNV infection

WNV was a gift from Dr. Karim Majzoub (IGMM, France). MEF cells were infected at the desired MOI of WNV in serum-free medium for 48 h. Cells were fixed using 4% PFA for 20 min at RT, permeabilized in 0.1% Triton X-100 for 5 min and blocked with 5% BSA solution for 30 min at RT. Cells were incubated with primary antibodies against dsRNA (Jena Bioscience, RNT-SCI-10010500) in PBS containing 0.5% BSA for 90 min at RT, followed by incubation with secondary antibodies Alexa Fluor 488-coupled goat anti-mouse IgG (Invitrogen, A11001) for 90 min at RT and nuclei staining with DAPI. The percentage of infected cells was determined using ImageXpress Pico. Conditioned media from infected cells was collected and MEF WT cells were incubated in it for 48 h. The same procedure as above was used to determine the percentage of cells infected by the conditioned media. Detailed information on antibodies is given in Supplementary Table 4.

### Prostaglandin ELISA

Intracellular PGE2 was extracted from the cells. Cells were pelleted and resuspended in 0.1 M potassium phosphate buffer (pH 7.4), then sonicated in the same buffer. The lysates were subsequently centrifuged at maximum speed for 30 min at 4 °C. The total concentration of PGE2 was determined in the supernatant using the Prostaglandin E2 ELISA kit (Cayman, 514010) and the 6-keto-PGF 1α EISA kit (Enzo Life Sciences, ADI-900-004) following the manufacturer’s instructions and the concentrations were normalized to the total protein. Graphs were plotted and statistical analysis was done using Graphpad Prism.

### Identification and analysis of STING and FADS sequences

The identification of a complete array of STING and FADS homologs across species was conducted using several strategies in parallel. Sequences previously reported were used to conduct searches using TBLASTN or BLASTP against datasets available in NCBI or Ensembl. Automatic annotations of the different genomes were also searched. Protein sequences for all genes analyzed here were collected from GenBank or Ensembl. All accession numbers are provided in the Plasmid constructs subsection. Sequence analysis and protein structure analysis was determined using SMART (http://smart.embl.de/). Additional analysis was performed using InterProScan (EBI) when relevant. Protein sequence alignments and similarity computing were conducted using Clustal Omega (EBI Web Services). Synteny analysis was performed using Genomicus (https://www.genomicus.bio.ens.psl.eu/genomicus-109.01/cgi-bin/search.pl), or NCBI or Ensembl, through extraction of relevant gene location from genome data viewers.

### Lipidomics by GC

The fatty acid composition of cells was determined according to previously published procedures^75,76^. Total lipids were extracted from cell pellets by using the Folch’s procedure^77^. Transmethylation of fatty acids and fatty alcohols into fatty acid methyl esters (FAME) and dimethylacetals (DMA), respectively, was achieved by using boron trifluoride in methanol according to Morrison and Smith^78^. FAMEs and DMAs were then extracted with hexane. The composition of FAMEs and DMAs was determined by gas chromatography (GC) coupled to flame ionization detection on a GC Trace 1310 (Thermo Scientific) gas chromatograph using a CPSIL-88 column (100 × 0.25 mm i.d., film thickness 0.20 μm, Varian, PN# CP7489). Hydrogen at 200 kPa was used as carried gas. The oven temperature was set at 60 °C for 5 min, then increased to 165 °C at 15 °C/min, hold at 165 °C for 1 min, increased to 225 °C at 2 °C/min, and then hold at 225 °C for 17 min. The injector and the detector were maintained at 250 °C. Individual species of FAMEs and DMAs were identified by comparison to commercial and synthetic standards (Sigma-Aldrich, CRM47885).

Chromeleon 7 software (Thermo Scientific) was used to determine the relative quantities of FAMEs and DMAs. Graphs were plotted and statistical analysis (two-tailed Student’s *t* test) was done using Graphpad Prism. *n* = 7 (MEF^*MbSTING*^), *n* = 9 (MEF^*NvSTING*^), *n* = 6 (MEF^*DrSTING*^), *n* = 7(MEF^*XtSTING*^), *n* = 12(MEF^*MmSTING*^), and *n* = 10(MEF^*Sting-/-*^) biological replicates were analyzed for MEF cell lines. *n* = 5 (THP-1^*HsSTING-R232H*^), *n* = 6 (THP-1^*HsSTING-AQ*^), *n* = 8 (THP-1^*HsSTING-HAQ*^, THP-1^*HsSTING-R232H-∆CTT*^), and *n* = 7 (THP-1^*STING-/-*^) biological replicates were analyzed for THP-1. *n* = 5 (WT) and *n* = 7 (*STING-/-*) biological replicates were analyzed for *M. brevicollis*. *n* = 6 (control) and *n* = 6 (*STING-/-*) biological replicates were analyzed for *D. melanogaster*.

### Lipidomics by GC-MS

GC-MS was used for the targeted analyses of ^13^C_18_ labelled PUFAs. Deuterated LA (18:2 n-6 d4) from Cayman (79050-23-0) was used as the internal standard (IS). All other chemicals were of the highest commercially available grade and were obtained from Sigma-Aldrich. LC-MS/MS-grade solvents were purchased from Fisher Scientific. Cell pellets were suspended in 100 µL of PBS and mixed with 10 µL of C18:2 n-6 d4 (0.5 µg/µL) as the IS, 60 µL of potassium hydroxide (10 mol/L), and 1.2 mL of ethanol containing butylated hydroxytoluene (50 mg/L). After incubation at 56 °C for 45 min, total fatty acids were extracted by adding 1 mL of hydrochloric acid (1.2 M) and 3 mL of hexane. The organic phase was collected and evaporated to dryness under vacuum. Fatty acids were then derivatized at 37 °C for 30 min with 50 µL of pentafluorobenzyl bromide (10% in acetonitrile) and 50 µL of diisopropylethylamine (10% in acetonitrile). The resulting pentafluorobenzyl fatty acid esters (PFB-FA) were extracted with 1 mL of water and 2 mL of hexane. The organic phase was collected and evaporated to dryness under vacuum. The PFB-FAs were finally reconstituted in 100 µL of hexane, and 1 µL was injected onto an HP-5MS fused silica capillary column (30 m × 0.25 mm i.d., 0.25 µm film thickness; Agilent Technologies, PN# 19091S-433; SN# US 2597013H).

GC–MS analysis was performed using an Agilent 7890 A gas chromatograph equipped with a 7683 injector and coupled to a 5975 C Mass Selective Detector (Agilent Technologies). The GC operating conditions were as follows: helium was used as the carrier gas at a constant flow rate of 1.8 mL/min; the injector temperature was maintained at 250 °C in pulsed split mode (split ratio 10:1); the oven temperature was initially set at 140 °C, increased at 5 °C/min to 300 °C, and held for 7 min. The quadrupole and ion source temperatures were both maintained at 150 °C. The mass spectrometer was operated in negative chemical ionization (NCI) mode using methane as the reagent gas at a flow rate of 2 mL/min. Data were acquired in single ion monitoring (SIM) mode. Summary of retention times (RT) and mass/charge (m/z) ratios of each PFB-FA analyzed is given in Supplementary Table 5.

Data acquisition was performed using MSD ChemStation software (version E.02.02.1431, Agilent Technologies) and converted using MassHunter GC/MS Translator (version B.06.00.1116, Agilent Technologies). Data processing was subsequently carried out using MassHunter Quantitative Analysis software (version B.09.00, build 9.0.647.0; Agilent Technologies). Fatty acids were semi-quantified (µg per cell pellet) by calculating the response ratio of each analyte relative to the IS and multiplying this ratio by the amount of IS added (5 µg). *n* = 6 (MEF^*MbSTING*^, MEF^*XtSTING*^, MEF^*Sting-/-*^) and *n* = 5 (MEF^*NvSTING*^) biological replicates were analyzed for detection of omega-6 ^13^C-PUFAs. *n* = 6 (MEF^*MbSTING*^), *n* = 5 (MEF^*NvSTING*^, MEF^*XtSTING*^, MEF^*Sting-/-*^) biological replicates were analyzed for detection of omega-3 ^13^C-PUFAs. *n* = 5 biological replicates each of THP-1^*HsSTING-R232H*^, THP-1^*HsSTING-AQ*^, THP-1^*HsSTING-HAQ*^, THP-1^*HsSTING-R232H-∆CTT*^, and THP-1^*STING-/-*^ were analyzed for detection of omega-6 ^13^C-PUFAs. *n* = 5 biological replicates each of THP-1^*HsSTING-R232H*^, THP-1^*HsSTING-AQ*^, THP-1^*HsSTING-HAQ*^, THP-1^*HsSTING-R232H-∆CTT*^, and THP-1^*STING-/-*^ were analyzed for detection of omega-3 ^13^C-PUFAs.

### Model organisms

*Drosophila melanogaster* flies were reared on standard sucrose-cornmeal-yeast agar medium at 25 °C and a 12:12 h light:dark photoperiodic cycle. All utilized fly lines were free of Wolbachia. Fly stocks used were *dSTING*^*L76GsTer11*^ isogenized to the DrosDel *w*^*1118*^ background control^21,79^. Six replicates were prepared for each genotype (control and *STING-/-*). A 50:50 mix of male and female adult flies collected 3–5 days post eclosing was used in experiments. For sample collection, heads were sectioned from anesthetized adult flies using a scalpel and transferred to a 2 mL screw-cap tube (30 heads/tube). The tubes were then transferred to dry ice for rapid freezing and kept below −80 °C until downstream lipid analysis. Samples were then analyzed by GC as described in the previous sub-section.

*M. brevicollis* WT (ATCC, PRA-258) and *STING* knockout^14^ cell lines were co-cultured with feeding bacterium *P. shioyasakiensis* in a seawater-based media enriched with glycerol, yeast extract, peptone, and cereal grass. Cells were passaged daily in vented cultured flasks and incubated at 22 °C and 60% relative humidity. For treatment of choanoflagellate cells, early stationary phase cells were washed four times by centrifugation (2000 x g) in artificial seawater, and then resuspended to a concentration of 1 × 10^6^ cells/mL in growth media. For each biological replicate, 1.2 × 10^7^ cells were treated either with 100 µM 2’3’ cGAMP or water for 5 h. Cells were pelleted at 3000 x g and flash frozen for downstream lipid analysis. Samples were then analyzed by GC as described in the previous sub-section.

### Assessment of membrane lipid peroxidation

The Image-iT Lipid Peroxidation Kit (Invitrogen, C10445) was used for ratiometric assessment of lipid peroxidation by flow cytometry. MEF or THP-1 cells were treated with 100 μM cumene hydroperoxide (CHP) in Opti-MEM for 2 h at 37 °C for assessment of lipid peroxidation under high ROS concentration. Basal and CHP treated cells were stained with 10 µM Image-iT Lipid Peroxidation Sensor (BODIPY 581/591 C11) for 30 mins at 37 °C. Cells were washed and resuspended in ice cold FACS buffer (PBS, 10% FBS, 0.5 mM EDTA). Fluorescence intensities at: 581/591 nm (Texas Red filter set) for the reduced dye and 488/510 nm (traditional FITC filter set) for the oxidized dye were read using NovoCyte (Agilent Technologies) and the Lipid Peroxidation Index (∆R) was calculated as:

Lipid Peroxidation Index (∆R) = {Basal Texas Red intensity (Reduced)/Basal FITC intensity (Oxidized)} - {CHP treated Texas Red intensity (Reduced)/CHP treated FITC intensity (Oxidized)}

The gating strategy used for flow cytometry is given in Supplementary Fig 9C.

### RNA-seq analysis

Differential expression analysis was performed in R (v4.2.0). Differential expression was deemed significant with a log2 fold change greater than 1 and an adjusted *P*-value less than 0.05. Gene identifiers were converted to human-readable names using annotations from https://figshare.com/articles/dataset/Nematostella_vectensis_transcriptome_and_gene_models_v2_0/807696. KEGG pathway enrichment analysis was performed using clusterProfiler with KEGG Orthology (KO) annotations retrieved via KEGGREST.

A subset of 12 differentially expressed genes was selected based on their association with enriched KEGG pathways. The pheatmap package (https://github.com/raivokolde/pheatmap) was used to generate heatmap plots. Heatmaps are based on regularized log-transformed normalized counts, and *Z*-scores were scaled by row. Clustering was performed using Euclidean distance and the complete linkage method. Raw sequencing reads and normalized gene counts for the *M. brevicollis* dataset can be found at the NCBI Gene Expression Omnibus (GEO) under accession GSE174340^14^.

### Quantification and statistical analysis

The quantification of WB images was done using FIJI (v2.9.0). CellProfiler (v4.2.8) was used for quantification of immunofluorescence images. GraphPad Prism (v10.6.1) was used for data analyses, statistical tests, and graph plotting. ROUT method (Q = 1%) was used to identify and remove outliers. Statistical significance was calculated using either unpaired two-tailed Student’s *t* test or two-tailed Mann-Whitney test. *P* value < 0.05 was considered statistically significant. No corrections or adjustments were made for multiple t tests. Data are presented as mean ± standard error of the mean (SEM).

### Reporting summary

Further information on research design is available in the Nature Portfolio Reporting Summary linked to this article.

## Supplementary information

Transparent Peer Review file

Supplementary information

Reporting Summary

## Source data

Source data

## Data Availability

The GC lipidomics data generated in this study have been deposited in the Zenodo database under the accession code 21418191 (https://doi.org/10.5281/zenodo.21418190). The GC-MS lipidomics data generated in this study have been deposited in the Zenodo database under the accession code 21417924 (https://doi.org/10.5281/zenodo.21400879). The RNA-seq data used in this study are available in the Gene Expression Omnibus (GEO) database under accession code GSE174340. The protocol for native gel can be found on protocols.io [https://doi.org/10.17504/protocols.io.kxygxr9x4g8j/v1]. All data are included in the Supplementary Information or available from the authors, as are unique reagents used in this Article. The raw numbers for charts and graphs are available in the Source Data file whenever possible. Source data are provided with this paper.
