# Supplementary material for: Evolutionary conservation analysis reveals a primordial function of STING as a regulator of lipid metabolism via FADS2
Source: Nat Commun. 2026 Aug 14;17:9771. doi: 10.1038/s41467-026-76833-5 (PMC13572491; doi:10.1038/s41467-026-76833-5)
Supplement: Supplementary file 1 [file 41467_2026_76833_MOESM1_ESM.pdf]

nature portfolio

Peer Review File

Evolutionary conservation analysis reveals a primordial function of STING as a regulator of lipid metabolism via FADS2

Corresponding Author: Dr Nadine Laguette

This file contains all reviewer reports in order by version, followed by all author rebuttals in order by version.

Version 0:

Reviewer comments:

Reviewer #1

(Remarks to the Author)
Guha et al. show that STING has an evolutionarily conserved connection to lipid metabolism through physical association with FADS family desaturases, and they argue this metabolic role likely predates vertebrate interferon signaling. The cross-lineage interaction data, the domain logic pointing to the transmembrane region as sufficient for binding, and the observation that STING activation perturbs the metabolic readouts together make a provocative case that STING should be viewed as a homeostatic regulator that was later coopted into immune signaling rather than a purely immune adaptor.

Overall, this is an interesting paper with provocative findings and conclusions however the enthusiasm is dampened by some key weaknesses listed below:

The authors should consider strengthening the evolutionary claim by adding at least one layer of functional evidence in a native or near native context for the most distant homologs. Much of the core functional inference comes from heterologous expression in mammalian cells, which is a powerful normalization strategy but also risks importing mammalian cofactors, membrane composition, trafficking constraints, and stress responses that could create confound the results. Even a minimal demonstration that perturbing STING in a basal metazoan or unicellular eukaryote shifts endogenous fatty acid pools in the predicted direction would support the primordial function hypoteshsis.

The authors should consider tightening the mechanistic interpretation of “regulation of FADS activity” versus “association with a membrane protein that correlates with lipid changes.” In several places the work infers enzyme activity from steady state lipid pools and downstream transcriptional markers. Those readouts are meaningful but they are also one step removed from enzymology and can reflect changes in substrate availability, fatty acid uptake, elongation, turnover, or broad ER remodeling. A direct activity or flux oriented test, for example isotope tracing from precursor PUFAs into long chain products, or an in vitro or semi reconstituted assay that isolates desaturation from whole cell lipid dynamics, would clarify whether STING directly modulates desaturase catalysis or instead shifts pathway balance indirectly.

The authors should consider addressing more explicitly the striking directionality split where vertebrate STING suppresses FADS derived products while the invertebrate and unicellular homologs increase them in the same cellular background. This is one of the most interesting conceptual findings, but it also opens alternative explanations such as differential expression levels, altered stability of the tagged constructs, species specific folding and membrane insertion efficiency, or engagement of distinct compensatory pathways when foreign membrane proteins are overexpressed. A clearer normalization strategy, paired measurements of STING abundance and localization across lines, and tests that equalize expression or use multiple independent clones could help rule out artifact and sharpen the evolutionary narrative from “conserved regulation” to “conserved coupling with divergent sign.”

The authors should consider expanding the functional scope of the antiviral interpretation before concluding that lipid regulation does not correlate with antiviral activity. The current infection experiments are informative but narrow, and the biology they discuss later implies that lipid dependent restriction could be virus class specific, cell type specific, and sensitive to membrane remodeling requirements. A small panel that includes at least one positive strand RNA virus with known dependence on ER derived membranes, alongside an orthogonal readout of host lipid peroxidation or ferroptosis

linked susceptibility, would better match the conceptual claim that metabolic regulation and antiviral restriction are separable axes.

The authors should consider sharpening the translational implication by more concretely defining what would be targetable if one wanted to dissociate inflammatory signaling from metabolic effects. The paper identifies the transmembrane region as sufficient for FADS interaction and the C terminal tail as dispensable, but that still leaves open whether one can actually separate metabolic modulation from canonical activation, trafficking, and degradation. A focused mutational mapping of the membrane interface, especially around regions implicated in lipid binding, paired with functional assays that score metabolic outputs independently of downstream inflammatory signaling, would turn the therapeutic discussion from an interesting prospect into a testable framework.

Reviewer #2

(Remarks to the Author)
Although we think of STING as primarily involved in stimulating interferon genes, interferon is only found in vertebrates while STING is present across metazoans and in some unicellular eukaryotes. This paper works to pin down the original functions of STING.

The paper reprsents an incredibly impressive amount of work. Being perhaps slightly orthogonal to this specific field, there were some points around the organization and logical flow that confused me. The abstract really emphasizes exploration of "regulation of polyunsaturated fatty acids" and I was unclear on whether one could consider this as nested within processes associated with autophagy? Or independent, as suggested by the second to last sentence in the abstract? (but perhaps autophagy is not considered antiviral?) Overall, I wondered if some sort of schematic that laid out possible functions would be helpful at the start of the manuscript?

The logic that led to a focus on FADS2 also had me slightly confused, further detailed below; and while it did seem that a major thread was that functionality was tied to location (e.g., in the ER or not) the conceptual depth here slightly confused me - does one start with a mental model where all functions involved lipid metabolism (and including authophagy?) originated with STING on the ER (and signalling was not triggered by dsDNA? or ?), and then evolution lead to a reloction that allowed segregation of function? something like that? again, a schematic might help.

Figure 4I was very helpful - perhaps something like that could come early with signposting as each piece of evidence comes through indicating what thread it aligns with (and slightly more of a caption?)

Specific comments

L72 - NF-kB do rely on STING then? One can infer this from the next sentence but might be good to formally state it? I also generally wasn't sure what to make of this result through the paper - is it more of a side note? or an emergent feature of interaction with FADS2? eventually it seemed like the latter but e.g., the abstract points to "regulation of polyunsaturated fatty acid (PUFA) metabolism." which sounds (to the uninformed perhaps!) as if it could be anything.

L87 meaning that STING fails to regulate lipid metabolism in the context of a an interferon (dsDNA triggered?) response?

L91 I'm not sure how having 'lost its ability to induce IFN-responses secondarily' forms part of this chain of logic? Anything could lose function?

L109; L119 - I'm not sure what we should be concluding from these details? A focus on the CBD rather than CTT? Something else? How are the genes being considered 'representative' in L124? Does it flow from this? or the paragraphs above? How do we end up focussing on the FADS2 homologs?

L124: As a naive person in this area, I wasn't sure whether FADS2 is just extraordinarily well known and is a natural focus, or whether this somehow emerges from the sentences above - perhaps unpack a little?

That an interaction between STING and a FADS-like protein arose prior to the emergence of metazoans is concluded from co-immunoprecipitation. How conclusive is this? Does it require the ER anchoring invoked subsequently (L195)?

L214 - I don't think you mean 'comforts'

L247 - at first I thought that this piece of evidence, and the next paragraph essentially indicate that the two immune functions (autophagy vs. triggering interferon) are in conflict - and wondered if the heading of this section signpost this a bit more? however, L328 seems to indicate the opposite?

Finally, is there something to be learned from nematodes that do not have STING homologs? Seems like it precludes lots of activitiies.

Reviewer #3

(Remarks to the Author)
This manuscript by Guha et al investigates the evolutionary history of STING by examining its role in lipid metabolism across a broad phylogenetic range. Through comparative analyses, heterologous expression, and lipidomic profiling, the authors demonstrate that STING homologs from unicellular eukaryotes to vertebrates interact with fatty acid desaturase enzymes (FADS2) and modulate polyunsaturated fatty acid (PUFA) metabolism. Importantly, this function is uncoupled from type I interferon (IFN-I) signalling and appears to predate the emergence of STING's canonical immune role in vertebrates. While STING-mediated regulation of FADS2 has been reported previously, this study offers a conceptual advance by proposing that lipid metabolic control represents the ancestral function of STING, which was later co-opted for antiviral IFN-I signalling. However, in its current form, the data do not yet fully support this evolutionary conclusion.

Major comments:

1. The interaction between STING-FADS2 homologs is all derived from heterologous expression of constructs in HEK293T cells. Validation of STING-FADS2 interaction endogenously in at least one invertebrate model (e.g. *Drosophila melanogaster*), as this hasn't previously been demonstrated. Notably, DmSTING was shown to interact with DmACC and DmFASN but not DmFADS2 (PMID: 34467853). Such experiments are important to support the proposed evolutionary conservation of this interaction and to reduce concerns that the observed binding reflects overexpression artefacts in a non-native system.

2. Similarly, endogenous STING loss-of-functon(knockout or knockdown), ideally combined with rescue experiments, should be performed in an invertebrate model followed by targeted PUFA lipidomics. The study currently relies heavily on heterologous expression in mammalian cells, and the effects of different STING homologs on lipid profiles are not always consistent in direction or magnitude. Validation in a cognate invertebrate system would provide the most direct support for the claim that lipid metabolic regulation (via FADS2) represents a primordial function of STING.

3. The divergent effect of STING homologs on PUFAs makes it unclear whether this reflects direct changes in desaturase activity versus indirect effects on lipid metabolism. To provide a more direct evaluation of FADS2 activity and desaturation events, lipidomics experiments should be performed with 13C-labeled fatty acids (FAs) e.g. $^{13}C_{16}$-palmitate and $^{13}C_{18}$-stearate tracers (e.g. PMID: 33567271) in their MEF$^{Sting-/-}$ and THP-1$^{Sting-/-}$ lines at least but also ideally a relevant invertebrate model.

Minor corrections:

Figure 1F – the schematic is showing the domain structure of FADS2 and other FADS but are labelled STING. This needs to be corrected.

Version 1:

Reviewer comments:

Reviewer #1

(Remarks to the Author)
The authors have done a great job thoroughly addressing my comments and I congratulate them on this revised manuscript. I have no further comments.

Reviewer #2

(Remarks to the Author)
The revision has addressed all my comments.

Reviewer #3

(Remarks to the Author)
I have no more comments

Open Access This Peer Review File is licensed under a Creative Commons Attribution 4.0 International License, which permits use, sharing, adaptation, distribution and reproduction in any medium or format, as long as you give appropriate credit to the original author(s) and the source, provide a link to the Creative Commons license, and indicate if changes were made.
In cases where reviewers are anonymous, credit should be given to 'Anonymous Referee' and the source.
The images or other third party material in this Peer Review File are included in the article's Creative Commons license, unless indicated otherwise in a credit line to the material. If material is not included in the article's Creative Commons license and your intended use is not permitted by statutory regulation or exceeds the permitted use, you will need to obtain permission directly from the copyright holder.
To view a copy of this license, visit https://creativecommons.org/licenses/by/4.0/

Reviewer's Comments:

Reviewer #1 (Remarks to the Author)

Guha et al. show that STING has an evolutionarily conserved connection to lipid metabolism through physical association with FADS family desaturases, and they argue this metabolic role likely predates vertebrate interferon signaling. The cross-lineage interaction data, the domain logic pointing to the transmembrane region as sufficient for binding, and the observation that STING activation perturbs the metabolic readouts together make a provocative case that STING should be viewed as a homeostatic regulator that was later coopted into immune signaling rather than a purely immune adaptor.

Overall, this is an interesting paper with provocative findings and conclusions however the enthusiasm is dampened by some key weaknesses listed below:

The authors should consider strengthening the evolutionary claim by adding at least one layer of functional evidence in a native or near native context for the most distant homologs. Much of the core functional inference comes from heterologous expression in mammalian cells, which is a powerful normalization strategy but also risks importing mammalian cofactors, membrane composition, trafficking constraints, and stress responses that could create confound the results. Even a minimal demonstration that perturbing STING in a basal metazoan or unicellular eukaryote shifts endogenous fatty acid pools in the predicted direction would support the primordial function hypoteshsis.

To address this comment, we performed additional lipid profiling in organisms and meta-analyses of available RNAseq datasets. More precisely:

- We performed lipidomics analyses of WT and *Sting-/- M. brevicollis*, which showed a decrease in PUFAs (Figure 3K), especially omega-6 PUFAs (Extended Data Figure S4F) upon *Sting* ablation. Indirect assessment of delta-6-desaturase activity from the gamma-linolenic acid (GLA) to LA ratio showed significant reduction in delta-6-desaturase activity in the *Sting-/-*, confirming our results from the heterologous system (Figure 3L).
- We performed metanalyses of available RNAseq datasets (PMID: 34730512). Differential gene expression analysis showed downregulation of diverse lipid metabolism-associated genes in STING-deficient *M. brevicollis* (Figure 3M), supporting our data obtained in heterologous systems.

The authors should consider tightening the mechanistic interpretation of "regulation of FADS activity" versus "association with a membrane protein that correlates with lipid changes." In several places the work infers enzyme activity from steady state lipid pools and downstream transcriptional markers. Those readouts are meaningful but they are also one step removed from enzymology and can reflect changes in substrate availability, fatty acid uptake, elongation, turnover, or broad ER remodeling. A direct activity or flux oriented test, for example isotope tracing from precursor PUFAs into long chain products, or an in vitro or semi reconstituted assay that isolates desaturation from whole cell lipid dynamics, would clarify whether STING directly modulates desaturase catalysis or instead shifts pathway balance indirectly.

As warranted by the reviewer, we performed flux analyses on both the engineered MEF (Figure 3F-G, Extended Data Figure S4B-C) and ThP1 (Figure 5H-I) cells treated with ^{13}C-LA and ^{13}C-ALA. We assessed the GLA to LA ratio (Figure 3F, Figure 5H) as well as summation of Fads2 products (PUFAs) to LA ratio (Extended Data Figure S4B) and the stearidonic acid (SA) to ALA to ratio (Figure 3G, Figure 5I) as well as summation of Fads2 products to ALA ratio (Extended Data Figure S4C), for omega-6 and omega-3 respectively in order to directly assess Fads2 activity. This supported our previous observations while at the same time revealing the difference in Fads2 activity with respect to omega-6 and omega-3 substrates.

The authors should consider addressing more explicitly the striking directionality split where vertebrate STING suppresses FADS derived products while the invertebrate and unicellular homologs increase them in the same cellular background. This is one of the most interesting conceptual findings, but it also opens alternative explanations such as differential expression levels, altered stability of the tagged constructs, species specific folding and membrane insertion efficiency, or engagement of distinct compensatory pathways when foreign membrane proteins are overexpressed. A clearer normalization strategy, paired measurements of STING abundance and localization across lines, and tests that equalize expression or use multiple independent clones could help rule out artifact and sharpen the evolutionary narrative from "conserved regulation" to "conserved coupling with divergent sign."

To address this concern, we provide a matrix showing paired analyses of STING abundance, interaction strength with FADS2, and activation/inhibition (Extended Data Item S3). All experiments within the manuscript are performed on polyclonal populations of cells (no clonal selection was performed), ruling out potential clonal effects. These data combined with the new in vivo data provided (see point 1), highlight the conserved regulation by STING of lipid metabolism, and indeed support that the coupling of STING and lipid metabolism is conserved, although the output may be divergent. This is now mentioned in the discussion (line 473-494).

The authors should consider expanding the functional scope of the antiviral interpretation before concluding that lipid regulation does not correlate with antiviral activity. The current infection experiments are informative but narrow, and the biology they discuss later implies that lipid dependent restriction could be virus class specific, cell type specific, and sensitive to membrane remodeling requirements. A small panel that includes at least one positive strand RNA virus with known dependence on ER derived membranes, alongside an orthogonal readout of host lipid peroxidation or ferroptosis linked susceptibility, would better match the conceptual claim that metabolic regulation and antiviral restriction are separable axes.

To address this concern, we have performed experiments using the vesicular stomatitis virus (VSV, negative strand RNA virus) and West Nile Virus (WNV, positive strand RNA virus) coupled to measurements of infectivity and of gene expression (see Figure 4C for experimental scheme and F-G for results). We found that expression of DrSTING and XtSTING did not prevent infection by VSV and WNV effectively, as compared to MmSTING (Figure 4F-G), while expression of MbSTING led to significantly higher susceptibility to infection by both (Figure 4F-G). Moreover, expression of MbSTING in cells also allowed higher viral replication by VSV (Figure 4F). This higher infectibility of ssRNA viruses in cells expressing MbSTING cannot be explained by loss of IFN-inducing function of STING alone. These data are in contrast to what was observed with the HSV-1 DNA virus, that was restricted by MbSTING. Thus, together, these data show that MbSTING fosters an antiviral state against dsDNA viruses but not against ssRNA viruses.

Lipid peroxidation (LPO) may be influenced by multiple factors, including the basal lipid composition of cells. Therefore, to address the reviewer's demand, we used LPO measurement to assess the consequences of the expression of the STING homologs. To mimic high ROS levels seen upon viral infection, we treated the cell lines with the peroxidation inducer cumene hydroperoxide (CHP). We found that the lipid peroxidation index (ΔR), was largely influenced by the total PUFA content of the cell lines (Figure 4H), except in case of MbSTING which had the lowest ΔR. This is now mentioned in the discussion (line 514-524).

The authors should consider sharpening the translational implication by more concretely defining what would be targetable if one wanted to dissociate inflammatory signaling from metabolic effects. The paper identifies the transmembrane region as sufficient for FADS interaction and the C terminal tail as dispensable, but that still leaves open whether one can actually separate metabolic modulation from canonical activation, trafficking, and degradation. A focused mutational mapping of the membrane interface, especially around regions implicated in lipid binding, paired with functional assays that score metabolic outputs independently of downstream inflammatory signaling, would turn the therapeutic discussion from an interesting prospect into a testable framework.

Recent papers (PMID: 41639452) have highlighted the complexity of interactions between STING and lipids, highlighting its strong dependency of phosphoinositide and cholesterol for its stability, membrane anchoring, and trafficking. Our data show that the STING-FADS2 interaction likely takes place inside the membrane via the hydrophobic TMD. Mutational analyses of TMD are likely to destabilize STING causing aberrant localization and preclude reliable assessment of its impact on lipid metabolism. As a surrogate for such mutation analyses we now performed functional analyses of the STING delta CBD delta CTT mutant of STING. Cells stably expressing this mutant were analyzed (gene expression and lipidomics)

We found that expression of the TM-only STING, although at low levels, was sufficient to partially restore FADS2 inhibition (See Extended Data Figure S4K-O).

Reviewer #2 (Remarks to the Author)

Although we think of STING as primarily involved in stimulating interferon genes, interferon is only found in vertebrates while STING is present across metazoans and in some unicellular eukaryotes. This paper works to pin down the original functions of STING.

The paper reprsents an incredibly impressive amount of work. Being perhaps slightly orthogonal to this specific field, there were some points around the organization and logical flow that confused me. The abstract really emphasizes exploration of "regulation of polyunsaturated fatty acids" and I was unclear on whether one could consider this as nested within processes associated with autophagy? Or independent, as suggested by the second to last sentence in the abstract? (but perhaps autophagy is not considered antiviral?) Overall, I wondered if some sort of schematic that laid out possible functions would be helpful at the start of the manuscript?

In order to clarify the interconnection of ancient STING homologs with antiviral immunity, the abstract was modified (line 33-35). Reviews summarizing STING function are cited in the introduction to allow a better understanding of the interdependence of phenotypes. Regulation of lipid composition by STING is indeed a process that can regulate membrane-related STING functions such as autophagy. We have also modified the abstract to broaden the scope to lipid metabolism (beyond PUFA metabolism) (line 30-31).

The logic that led to a focus on FADS2 also had me slightly confused, further detailed below; and while it did seem that a major thread was that functionality was tied to location (e.g., in the ER or not) the conceptual depth here slightly confused me - does one start with a mental model where all functions involved lipid metabolism (and including authophagy?) originated with STING on the ER (and signalling was not triggered by dsDNA? or ?), and then evolution lead to a reloction that allowed segregation of function? something like that? again, a schematic might help.

The focus on FADS2 stems from our previous work that identified STING as a partner of FADS2 and STING-dependent regulation of FADS2 activity as central to lipid homeostasis in mouse (PMID: 34986331). This function is a homeostatic function of STING (as opposed to being induced by STING activation). We have now clarified in the introduction that all immune related functions reported as of today are associated with STING activation, while STING-dependent lipid regulation is disrupted by STING activation (line 83-84). Our data (and prior work) indicate that STING translocation upon stimulation is a phenomenon conserved across homologs. Such STING relocation, in the case of homologs that do not activate inflammatory pathways, would disrupt lipid production, a process that may also have implications for immune-related process. These matters and biological/evolutionary implications are discussed in more depth (lines 499-516).

Figure 4I was very helpful - perhaps something like that could come early with signposting as each piece of evidence comes through indicating what thread it aligns with (and slightly more of a caption?)

A similar scheme is provided in Figure 1A, to indicate already known functions. In response to the issue raised by the reviewer, we have now included a scheme summarizing the interaction data (Extended Data Figure 2G). Figure 3-5 all assess functional conservation that remain summarized in Figure 5J with more detailed captions. In addition, Extended Data Item S3 also provides a summary of the data, normalized across approaches and may help in the understanding the data.

Specific comments

L72 - NF-kB do rely on STING then? One can infer this from the next sentence but might be good to formally state it? I also generally wasn't sure what to make of this result through the paper - is it more of a side note? or an emergent feature of interaction with FADS2? eventually it seemed like the latter but e.g., the abstract points to "regulation of polyunsaturated fatty acid (PUFA) metabolism." which sounds (to the uninformed perhaps!) as if it could be anything.

STING-mediated NF-KB activation is one of its canonical immune-related functions also found in invertebrates. This is stated in lines 55 and 78-81. This is a process-associated with STING activation, and is not related to STING-dependent lipid regulation/PUFA regulation with is a homeostatic function that is disrupted by activation, as clarified above.

L87 meaning that STING fails to regulate lipid metabolism in the context of a an interferon (dsDNA triggered?) response?

Indeed, STING dose not inhibit FADS2 upon stimulation with agonists.

L91 I'm not sure how having 'lost its ability to induce IFN-responses secondarily' forms part of this chain of logic? Anything could lose function?

The fact that a function is present in ancient species, and lost in some branches of evolution while conserved in others suggests a loss of function (more likely than *de novo* acquisition in several phylogenetic lineages). That IFN regulation is lost suggests that this function is dispensable and supports the hypothesis that it does not represent its primordial function. This has now been clarified in the chain of logic (line 98-101).

L109; L119 - I'm not sure what we should be concluding from these details? A focus on the CBD rather than CTT? Something else? How are the genes being considered 'representative' in L124? Does it flow from this? or the paragraphs above? How do we end up focussing on the FADS2 homologs?

The choice of STING alleles for this study relies on the conservation of domains. To assess which region of STING may be involved in FADS2 regulation, we chose representatives based on the conserved domains. This is clarified in line 132. The FADS2 homologs used are those present in the organisms in which we wish to assess interaction with STING (*i.e.* the ones selected in lines 133-136). This is clarified at lines 146-147.

L124: As a naive person in this area, I wasn't sure whether FADS2 is just extraordinarily well known and is a natural focus, or whether this somehow emerges from the sentences above - perhaps unpack a little?

The focus on FADS2 stems from our previous work (PMID: 34986331) that identified STING as a partner of FADS2 and STING-dependent regulation of FADS2 activity as central to lipid homeostasis. This has been clarified in line 138.

That an interaction between STING and a FADS-like protein arose prior to the emergence of metazoans is concluded from co-immunoprecipitation. How conclusive is this? Does it require the ER anchoring invoked subsequently (L195)?

This conclusion is supported not just by co-immunoprecipitations, but also functional analyses that shows the conservation of the regulation of desaturase activity by STING homolog from *Monosiga*

brevicollis (Figure 3C-L). Now we have also included additional lipid profiling in organisms and meta-analyses of available RNAseq datasets on the *Monosiga brevicollis* and demonstrated that the function of STING on lipid metabolism regulation is conserved. More precisely:

- We performed lipidomics analyses of WT and *Sting-/- M. brevicollis*, which showed a decrease in PUFAs (Figure 3K), especially omega-6 PUFAs (Extended Data Figure S4F) upon *Sting* ablation. Indirect assessment of delta-6-desaturase activity from the gamma-linolenic acid (GLA) to LA ratio (Figure 3L) showed significant reduction in delta-6-desaturase activity in the *Sting-/-*, confirming our results from the heterologous system (Figure 3K).
- We performed metanalyses of available RNA-seq datasets (PMID: 34730512). Differential gene expression analysis showed downregulation of diverse lipid metabolism-associated genes in STING-deficient *M. brevicollis* (Figure 3M), supporting our data obtained in heterologous systems.

Similar data were obtained on Drosophila melanogaster (WT and *Sting-/-*) showing global lipid metabolic dysregulation (Extended Data Figure S4H-I).

L214 - I don't think you mean 'comforts'

This has been modified (line 225)

L247 - at first I thought that this piece of evidence, and the next paragraph essentially indicate that the two immune functions (autophagy vs. triggering interferon) are in conflict - and wondered if the heading of this section signpost this a bit more? however, L328 seems to indicate the opposite?

This has been rephrased: there is no conflict between these functions. STING-related autophagy is a highly conserved function across holozoans while IFN responses is confined to vertebrates (that also regulate autophagy through STING).

Finally, is there something to be learned from nematodes that do not have STING homologs? Seems like it precludes lots of activitiies.

Nematodes, especially parasitic nematodes, often exhibit extensive loss of lipid metabolizing genes or extremely low expression levels, while specializing in scavenging lipids from hosts. Essentially, there is a trade-off between metabolic autonomy and host-dependent efficiency in these systems. We have decided to consider such systems as exceptions and have not discussed them further.

Reviewer #3 (Remarks to the Author):

This manuscript by Guha et al investigates the evolutionary history of STING by examining its role in lipid metabolism across a broad phylogenetic range. Through comparative analyses, heterologous expression, and lipidomic profiling, the authors demonstrate that STING homologs from unicellular eukaryotes to vertebrates interact with fatty acid desaturase enzymes (FADS2) and modulate polyunsaturated fatty acid (PUFA) metabolism. Importantly, this function is uncoupled from type I interferon (IFN-I) signalling and appears to predate the emergence of STING's canonical immune role in vertebrates. While STING-mediated regulation of FADS2 has been reported previously, this study offers a conceptual advance by proposing that lipid metabolic control represents the ancestral function of STING, which was later co-opted for antiviral IFN-I signalling. However, in its current form, the data do not yet fully support this evolutionary conclusion.

Major comments:

1. The interaction between STING-FADS2 homologs is all derived from heterologous expression of constructs in HEK293T cells. Validation of STING-FADS2 interaction endogenously in at least one invertebrate model (e.g. *Drosophila melanogaster*), as this hasn't previously been demonstrated. Notably, DmSTING was shown to interact with DmACC and DmFASN but not DmFADS2 (PMID: 34467853). Such experiments are important to support the proposed evolutionary conservation of this interaction and to reduce concerns that the observed binding reflects overexpression artefacts in a non-native system.

In fact, the fads2 homolog in drosophila CYB5R_DROME (P19967) was shown to interact with dmSTING *in vivo* in the mentioned paper (PMID: 34467853). It is important to stress here that, among invertebrates, FADS (and to a lesser extent, STING) sequences of arthropods (comprising insects, crustaceans, arachnids, and myriapods) are particularly divergent, suggesting that the associated functions may have evolved in a distinct way in this group. As reported in figure 1G-H of our manuscript, the closest sequence to human FADS2 found in drosophila displays extremely low similarity to this protein [suggested to localize in the mitochondria and perform divergent enzymatic activities (PMID: 10899459)]. DmSTING possibly modulated those divergent metabolic activities. Other invertebrate fads2 homologs tested in our interaction studies also most likely do not have delta6-desturase activity (See line 477-486). Nonetheless, we now performed lipidomics analyses on WT and STING deficient drosophila heads. We found global lipid metabolic dysregulation manifested by increased saturated fats in the absence of STING (Extended Data Figure S4H), most likely due to an increase in ACC/FASN activity (Extended Data Figure S4I), with which DmSTING is known to interact (PMID: 34467853).

We also included a more extensive discussion on the case of arthropods (line 477-486).

2. Similarly, endogenous STING loss-of-function (knockout or knockdown), ideally combined with rescue experiments, should be performed in an invertebrate model followed by targeted PUFA lipidomics. The study currently relies heavily on heterologous expression in mammalian cells, and the effects of different STING homologs on lipid profiles are not always consistent in direction or magnitude. Validation in a cognate invertebrate system would provide the most direct support for the claim that lipid metabolic regulation (via FADS2) represents a primordial function of STING.

To address this comment, we performed additional lipid profiling in organisms and meta-analyses of available RNAseq datasets. More precisely:

- We performed lipidomics analyses of WT and *Sting-/- M. brevicollis*, which showed a decrease in PUFAs (Figure 3K), especially omega-6 PUFAs (Extended Data Figure S4F) upon *Sting* ablation. Indirect assessment of delta-6-desaturase activity from the gamma-linolenic acid (GLA) to LA ratio showed significant reduction in delta-6-desaturase activity in the *Sting-/-*, confirming our results from the heterologous system (Figure 3L).
- We performed metanalyses of available RNAseq datasets (PMID: 34730512). Differential gene expression analysis showed downregulation of diverse lipid metabolism-associated genes in STING-deficient *M. brevicollis* (Figure 3M), supporting our data obtained in heterologous systems.

As stated above, we also provide data on *Sting-/- D. melanogaster* and rescue.

3. The divergent effect of STING homologs on PUFAs makes it unclear whether this reflects direct changes in desaturase activity versus indirect effects on lipid metabolism. To provide a more direct evaluation of FADS2 activity and desaturation events, lipidomics experiments should be performed with 13C-labeled fatty acids (FAs) e.g. $^{13}C_{16}$-palmitate and $^{13}C_{18}$-stearate tracers (e.g. PMID: 33567271) in their MEF$^{Sting-/-}$ and THP-1$^{Sting-/-}$ lines at least but also ideally a relevant invertebrate model.

We have now performed flux analyses on both the engineered MEF (Figure 3F-G, Extended Data Figure S4B-C) and ThP1 (Figure 5H-I) cells treated with 13C-LA and 13C-ALA. We assessed the GLA to LA ratio (Figure 3F, Figure 5H) as well as summation of Fads2 products (PUFAs) to LA ratio (Extended Data Figure S4B) and the stearidonic acid (SA) to ALA to ratio (Figure 3G, Figure 5I) as well as summation of Fads2 products to ALA ratio (Extended Data Figure S4C), for omega-6 and omega-3 respectively in order to directly assess Fads2 activity. This supported our previous observations while at the same time revealing the difference in Fads2 activity with respect to omega-6 and omega-3 substrates.

Minor corrections:

Figure 1F – the schematic is showing the domain structure of FADS2 and other FADS but are labelled STING. This needs to be corrected.

We apologize for this mistake that will be corrected in the revised manuscript.
