# Supplementary material for: Evolutionary conservation analysis reveals a primordial function of STING as a regulator of lipid metabolism via FADS2
Source: Nat Commun. 2026 Aug 14;17:9771. doi: 10.1038/s41467-026-76833-5 (PMC13572491; doi:10.1038/s41467-026-76833-5)
Supplement: Supplementary file 2 [file 41467_2026_76833_MOESM2_ESM.pdf]

Evolutionary conservation analysis reveals a primordial function of STING as a regulator of lipid metabolism via FADS2

Soumyabrata Guha[1], Joanna Re[1], Stéphane Grégoire[2], Morgane Chemarin[1], Adeline Augereau[1], Yasmine Messaoud-Nacer[1], Pierre Le Hars[1], Hanane Chamma[1], Victoria Bergas[3], Collin McNairy[4], Jenny Nguyen[5], Jennifer Barrat[1], Jean-Paul Pais de Barros[3], Arielle Woznica[4], Niyazi Acar[2], Isabelle K Vila[1], Pierre Boudinot[6], and Nadine Laguette[1*]

[1] Institut de Génétique Moléculaire de Montpellier (IGMM), Université de Montpellier, CNRS UMR5535, Montpellier, France
[2] Eye & Nutrition Research Group, Université Bourgogne Europe, CHU Bourgogne, Public Health Department, Institut Agro, CNRS, INRAE, UMR CSGA, 21000 Dijon, France
[3]Université Bourgogne Europe, INSERM, BioSanD US 58, Plateforme Diviomics, Dijon, France
[4]The University of Texas at Austin, Department of Molecular Biosciences, Texas, USA
[5]Université de Strasbourg, CNRS UPR9022, Institut de Biologie Moléculaire et Cellulaire, Strasbourg
[6]Université Paris-Saclay, INRAE, UVSQ, VIM, Jouy-en-Josas, France

*Correspondence: nadine.laguette@cnrs.fr

Contains Supplementary Figure 1 – 9 and Supplementary Table 1 – 5.

```
HOMSA      -------MPHSSLHPSIPCPRGHGAQKAALVLLSACLVTLW-------GLGEPP-EHTLR 45
XENTR      -MASIRNTLATQNRQIIPERRGKRATKMACVLAIGSILFVW-------ILGKGK-YSGAQ 51
DANRE      -------MSVMGEDALVPRARSRLPVMCAAGLGFLTLAVAW-------LLDSDK-FSERA 45
DROME      -MAIASNVVEAGNAV--RAEK-------------GRKYFYF----R-KMIGDYIDTSIRI 39
CRAGI      -------MEKNGAHSFLSDTPVTSLTMSVPVLRHPHVYHAFISY------CADADT---- 43 TIR
NEMVE      ----MRRAEENNGFGTIPKRRNQHTPFYASIGMIVVIIVAFTSYHI-TSYGDDRNRAMRQ 55
MONBE      MMVNLSDLSH------LSQRGWAQVFVTLAAAAISIFYFSFSPFEVAAGICASIAAAGAV 54
                                                   :

HOMSA      --Y-LVLHLASLQLGLLLNGVCSLAEEL----RHIHSRYRGSYWRTVRA--CLGCP---- 92
XENTR      LIYRMAINFAISQGCCLVTCACELTEEI----KHLHTRYNGHYWRALKA--SFNLS---- 101
DANRE      GIIAFG-----LMLERFIYCICLLAEELL---FHSRQRYHGRMSEIFRA--CFRGS---- 91
DROME      ----VATVFLADLLLRLYRCVVEYGSNG------RYYLPEDRLWIILRRSCTYNNR---- 85
CRAGI      -------------------------------SHARTILDSVESRGFTC--CFAERDFLP 69
NEMVE      YSFTFSLAYLAFLVGELLRRCCLFAEEY----RHIETRYNGSLKKAIQT--TFSFG---- 105
MONBE      --------PLLFDAVHYLIAFCISAPDARPPLRTVWTKTRLQRWSGLSI----------- 95
                                                         .

HOMSA      --L--RRGALLLLSIYFYYS--LPNA--------------------VGPPFTW-MLAL-L 124
XENTR      --C--AAFVTAI-LCYVFYE--PKLM--------------------ASLPLTI-DITL-T 132
DANRE      --G--ILGMCAIFLMLMLGG--VSFS--------------------V---KQWSHFNL-M 121
DROME      ----SIYLIVGF-LLVAFFRISVT-------GNYRNVMPTTLFLFQMP------------ 121
CRAGI      GECTSDVVVDAI-HC---------------------SKNVILVISPASLQSEWSKFEMLM 107
NEMVE      --HNNVLFVASLLFFVVFVASNDPNGSSSVIQGNSTAEPHTEMRQTSGWQGLWGQFIISA 163
MONBE      ------L-TFIVLACGLYFASPSPMS----------------------ASNLFAVFALSL 126
                      .

HOMSA      -----------GLSQALNILLGLKG-------------------------LAPAEISAV 147
XENTR      -----------LLSWLFCWILGIQG-------------------------PTPATISEI 155
DANRE      -----------CAGYMLLNSLGVLG-------------------------PAPVEISEI 144
DROME      ----------------LYWIWSFTD-----------------------MDQSTLSYSHWI 142
CRAGI      AVDDSHQRNNVCLVPVLLGGVKVDDLPPPLRPLTCIELMDDFRNTDDIIQAISKPEDTWE 167
NEMVE      -----------LLTPLVVHLLGLRE-------------------------LSKVEESQL 186
MONBE      CNSV-----FSAMVAPRCGRLEVVD------------------------LATH-----P 151
                                 .                             :

HOMSA      CEKGNFNVAHGLAWSYYIGYLRLILPELQARI-----RTYNQHYNNLLRGAVSQRLYILL 202
XENTR      TEIKQLNVAHGLAWSYYVGYLQFVLPALKESI-----QKFNEENHNLLKFPETCRLHILI 210
DANRE      CEAKKMNVAHGLAWSFYIGYLKFLLPALEVNV-----REYSRRE-----RLSSPRLHILL 194
DROME      RDSHGLDYAAGMASNYFHGYLKLSLPERKDDGLKHRLAMYEDKNN---VTFGIKRLVILI 199
CRAGI      SLLPVGNLAHGFAWGYYYGYLKIILPDLDKTV-----RQWRRVNN--AEGRMSEKLFLFF 220
NEMVE      NEKENKNVADGLAWSYYFGYLKFVLPELEKQI-----EKTSKFRS---KEKFVKKMFILI 238
MONBE      NTVLFRDFGAASAMSYWHGYLQHIAGDRAALE-----ERFRDSD--VGGMRVPRKLYILV 204
                 : . . * .:: ***:                                :: ::.

HOMSA      PLDCGV----PDNLSMADPNIRFLDKLPQQTGDRAGIKDRVY-SNSIYELLE-NGQRAGT 256
XENTR      PLSCRL----YGDLKDVDENITFLKEIPPLYIDRAGIKGRVF-KNNVYRILD-EDGRPYN 264
DANRE      PLNARV----PSKPEEEDTNVVFHENLPDLKLDRAGVRKRSY-TNSVYKITH-N-NETFS 247
DROME      PDEMFVNGVLESHL------LDKAEPLETQFINRAGVY-RPF-KHDVYRMNKKVNGRTYY 251
CRAGI      PQSCRC----RDSIADESSLIKHRGHLPIITKDRAGIIERQY-KNTIYSVTD-DNGEDYF 274
NEMVE      PSNCFWDDKIPGSDYDPQNRITFEGNTEPLEKTRGGVFLRHY-KHSVYEIKD-GENEPWF 296
MONBE      PQDCEVNA----SDNKYEGLKATEHYISPKPITIGGVVDREMGKHTLYTPSESAETASVA 260
           * .                               .*:  *   .: :*   .

HOMSA      CVLEYATPLQTLFAMSQYSQAGFSR--EDRLEQAKLFCRTLEDILADAPESQN-----NC 309
XENTR      CIVEYATPLASLLKMTDIPSAAFSA--DDRLQQTKLFYRTLKDILENAHELQN-----TY 317
DANRE      CILEYATPLLTLYQMSQESSAGFGE--RERKQQVLLFYRTLSQILDNSLECRN-----RY 300
DROME      FAVEGATPMISFFDATYSNLSGTWQMQELKREIWIKFYKHLKELITTWPETRD-----LV 306
CRAGI      FAGEYIGVIHTMFEMEQNATTGLQT--REKYVQSMRFYLTLKRILDTDPECSK-----KC 327
NEMVE      CIMEYATPLLTLYDMSVAQPGELSR--EERDAQVVVFLRKLQDILEGDRACQG-----KY 349
MONBE      FAMELASPLNTMKNALKDAAEGLLE------LQSEAFYLTLRGILKQEGVLDQGNDEGMI 314
              *    : ::                         *   *  ::

HOMSA      RLIAYQEP---ADDSSFSLSQEVLRHLRQEEKEEVTVGSL-KTSAVPSTS---------- 355
XENTR      RLIVYEDFPETKDHSRHLLSQEILKHIRQQHSEEYSML---------------------- 355
DANRE      RLILLNDE---HTGDPHYLSRELFQNLKQQDGEIFMDPTN-EVHPVPEEGPVGNCNGALQ 356
DROME      ELIIYNSHDSKGNLV--DVGELLVAHMQNKTKT------------IDEISN--------- 343
CRAGI      KIVFYKDVNNSSDAMPKLICNEIKNQLRKESSDDTTVCMTPFNSPFPSISSPDFARCSLK 387
NEMVE      ELVTFSPDRDLADVMLRKLKDSELEIGG-------------------------------- 377
```

```
MONBE      KLVWGKNRSEVLKKMRAEDQYAMP----------------------------------- 338


HOMSA      ------TMSQEPELLISGMEKPLPLRTDFS---------------   379
XENTR      ---------------------------------------------   355
DANRE      ATFHEEPMSDEPTLMFSR---PQSLRSEPVETTDYFNPSSAMKQN   398
DROME      ---------------------------------------------   343
CRAGI      SPSSTNMVKSEPNIYREESGKTKSVERG-----------------   415
NEMVE      ---------------------------------------------   377
MONBE      ---------------------------------------------   338
```

Supplementary Figure 1. Alignment of STING homolog sequences from indicated species. Residues highlighted in yellow: transmembrane (TM) domain, in purple: Toll/interleukin-1 receptor (TIR) domain, in grey: cyclic dinucleotide (CDN)-binding, and in green: carboxyterminal tail (CTT). Sequences were aligned with the CLUSTAL O(v1.2.4) multiple sequence alignment tool.

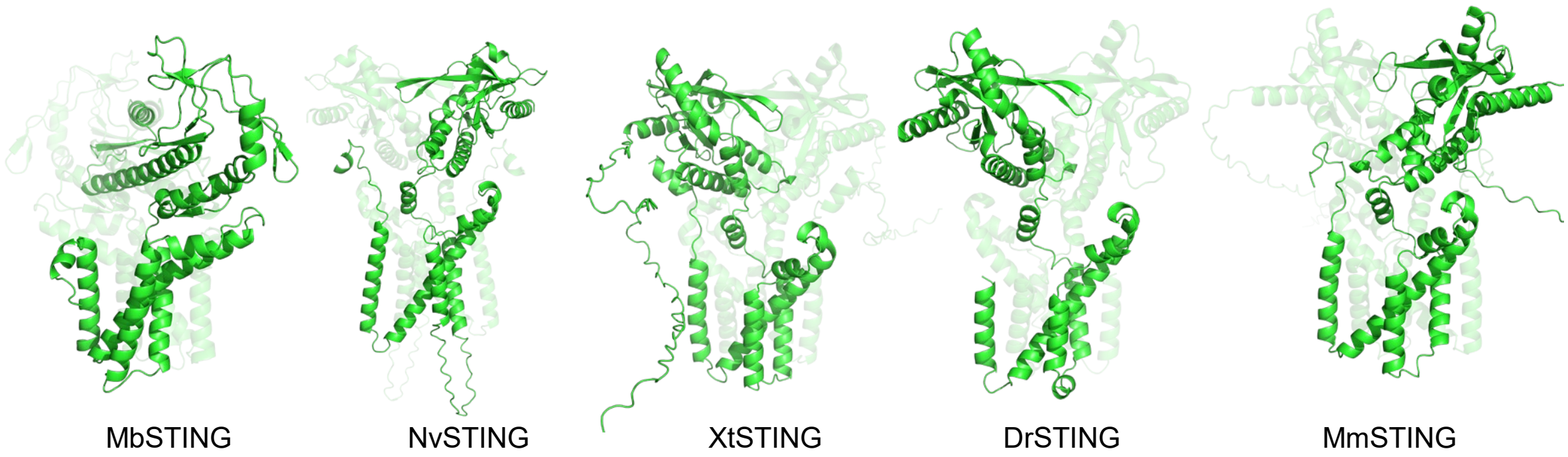

Supplementary Figure 2. Comparative analyses of STING homologs.
3D structures of dimers of STING homologs from the indicated species, as predicted by Alphafold2, were rendered using Pymol.

```
HOMSA           MGKGGNQ----GEGAAEREVS--VPTFSWEEIQKHNLRTDRWLVIDRKVYNITKWSIQHP 54
XENTR           MGMGGQS----GEGCSSGNCVKPEARYSWEEIQKHNLKTDKWLVIERKVYNITQWVKCHP 56
DANRE           MGGGGQQ----TDRITDT--NGRFSSYTWEEVQKHTKHGDQWVVVERKVYNVSQWVKRHP 54
DROME           MVIEEWKKSGIATKFPTYRNSALITTHSWQKGKRQDDGAEGLWRINDGIYDFTSFIDKHP 60
CRAGI           MKPGPSKEMG-KEVYSDVSMAKENKHYTVEEVKRHNERDDNWLMINGKVYDLSRWAKKHP 59
NEMVE           --------------MTVALQRRTPKVYTLDEVKEHCSKGDCWVVVEDSVYDLSKWIGHHP 46
MONBE           ----------------------------------------MKICIDQEWYDLTKWAKYHP 20
                                                            ::   *:.: :   **

HOMSA           GGQRVIGHYAGEDATDAFRAFHPDLEFVGKFLKPLLIGELAPEEPS----QDHGKNSKIT 110
XENTR           GGMRVIGHYAGEDATDAFHAFHPDKNFVRKFLKPLYVGELAENEPS----QDRDKNAQQV 112
DANRE           GGLRILGHYAGEDATEAFTAFHPNLQLVRKYLKPLLIGELEASEPS----QDRQKNAALV 110
DROME           GGPFWIRETKGTDITEAFEAHHLTTAPEKM-IAKYKVRDAAEPRIYTLTLEEGGFYKTLK 119
CRAGI           GGAKILGHYAGEDATDAWTAFHNDKGYVSKFMKALYIGDVEDWDQ---------NETEIK 110
NEMVE           GGELPILYMAGRECTDVFKAFHPAWVFTKK-LPAFKIGKLDDT-------RKEKKETSLS 98
MONBE           GGVRILERFDNQDATDHFYSLHSTDAIR-KF------KAMRPTETK----EDVPEVLPID 69
                **   :    . : *: : : *

HOMSA           EDFRALRKTAEDMNLFKTNHVFFLLLLAH-IIALESIAWFTVFYFGNGWIPTLITAFVLA 169
XENTR           EDFRALRKTAEDMGLFKSNPAFFIVYLFH-ILLIEFLAWCTLHYFGTGWIPTILTVLLLT 171
DANRE           EDFRALRERLEAEGCFKTQPLFFALHLGH-ILLLEAIAFMMVWYFGTGWINTLIVAVILA 169
DROME           ERVREQLKTIDKRPKKKSDLI-------HLGLVVSLYL---LG-IASAKYNSLLALVLAS 168
CRAGI           KDFRKLRKYVEESGLMKVNPLFYVLHLLH-ILALEVLAYLVVWHWEGSMAAFFVAAVLLA 169
NEMVE           EDFEKLRQEIIEGGGLQTNYWFYIRLASMLALLFASIIYCVVF--SNNVYIQVAAGILVA 156
MONBE           ASFRELRAKLMDDGWWDRDLL------TELGILIPIFAMCIIG-TALAWSHPVISVLLIS 122
                  ..            . :            : .       :          .   .: :

HOMSA           TSQAQAGWLQHDYGHLSVYRKPKWNHLVHKFVIGHLKGASANWWNHRHFQHHAKPNIFHK 229
XENTR           ISQAQAGWLQHDFGHLSVFKKSKWNHLIHKFIIGHLKGASANWWNHRHFQHHAKPNIFRK 231
DANRE           TAQSQAGWLQHDFGHLSVFKTSGMNHLVHKFVIGHLKGASAGWWNHRHFQHHAKPNIFKK 229
DROME           VALCWTVIVSHNYFHR----RDNWQ----MYAFNLGMMNFAAWRVSHALSHHIYPNSYF- 219
CRAGI           TAQAQAGWSQHDYGHMSVFPSHKMNHIFHHIVIGHIKAASSHWWNFRHFQHHAKPNVIKK 229
NEMVE           VFWQQMAFIGHDAGHHAIFHDEQWDDRLGLVVGNLLTGVSIGWWKKSHNAHHVVTNSVEL 216
MONBE           VGMQQAGWLGHDMTHA---RDSRYNDFWLRYVSGWLNGFDRNWWSNKHNTHHVLTNHVNH 179
                          *:  *         :        .        *       **   *

HOMSA           DPDVNMLHVFVLGEWQPIEY----GKK------KLKYLPYNHQHEYFFLIGPPLLIPMYF 279
XENTR           DPDVNMVNVFVLGDTQPVEF----GKK------RIKYLPYNHQHLYFFLIGPPLLIPIYF 281
DANRE           DPDVNMLNAFVVGNVQPVEY----GVK------KIKHLPYNHQHKYFFFIGPPLLIPVYF 279
DROME           DLELSMFEPLLCWVPNPHIK-----SKLMRYVSWV--TEPVAYALAFFIQMGTRIFYSLR 272
CRAGI           DPDVDIAFLFLIGDYIPKSW----GSK------KLGFMPYNFQHSYFFFLGPPLLLPIYF 279
NEMVE           DPDIQHLPVLAVTDKFFNSIKSIYHDRVMHFDGLAKFFVRYQHHLYFLIMGLAR-FNLYA 275
MONBE           DPDIHVQPILYLWA--PLKQ--------------MDHFLRKYQYIYFP-----LPYTLLF 218
                * ::     :                                   :*          :

HOMSA           QYQIIMTMIVHKNWVDLA----WAVSYYIRFFITYIPFYGILGALLFLN--FIRFLESH- 332
XENTR           TIQIMKTMITRKDWVDLA----WSVSYYARFFITFVPFYGVLGSLALLN--AVRFIESH- 334
DANRE           QFQIFHNMISHGMWVDLL----WCISYYVRYFLCYTQFYGVFWAIILFN--FVRFMESH- 332
DROME           HTN-------ILYWHDLLPL-----TIPIAIYLGTGGSLGIWICVRQWL--AMTSIASFS 318
CRAGI           HVENIFFVFKRRDYVDLL----MTVTFFYKFFWLYGPYYGGWGAFGLYM--FMRFLESH- 332
NEMVE           QSF--L-LVLSKERVKLRVMEFVTMVLFWTWYLTLCSYLPTWSTRFAFV--FLAHFLAG- 329
MONBE           ASWRLE----SLKWS-------IANRDYKMFFLAILPGYV-WLALLPFKVVLASILVSGF 266
                                               :                       : :

HOMSA           WFVWVTQMNHIVM------EIDQEAYRDWFSSQLTATCNVEQSF-FNDWFSGHLNFQIEH 385
XENTR           WFVWVTQMNHLPM------VIDHEKYKDWLETQLAATCNIEPSF-FNDWFSGHLNFQIEH 387
DANRE           WFVWVTQMSHIPM------NIDYEKNQDWLSMQLVATCNIEQSA-FNDWFSGHLNFQIEH 385
DROME           FCLIGLNAAHHDPEIYHEG-DANREDRDWGLFQVDTIIDRGDLKWSQFLVLTHFGDHVLH 377
CRAGI           WFTWVTQMSHLPM------RVEKEKEEDWFSLQLATTCNVDSSP-FNDWFTGHLNFQIEH 385
NEMVE           IIHIQITLSHFSMETYNGLPLDAFKENRFLLSQMDTTMDIECDP-NLDFFHGGLQFQFEH 388
MONBE           LVAIVVTMSHESE------ELLLEREPSYVTNQFLTTRDVQCLDWVTEYLFGGMQYQLEH 320
                         *                  :   *. :  :          .   :  :. *

HOMSA           HLFPTMPRHNLHKIAPLVKSLCAK-HGIEYQEKPLLRALLDIIRSLKKSGK--------L 436
XENTR           HLFPTMPRHNYWKIAPLVRSLCSK-YNVTYEEKCLYHGFRDVFRSLKKSGQ--------L 438
DANRE           HLFPTVPRHNYWRAAPRVRALCEK-YGVKYQEKTLYGAFADIIRSLEKSGE--------L 436
DROME           HLFPTLDHGLLPALYPVLYQTLDEFKGHLRECNHIEHMIGQHKQLLRIEPNPRAPGAGK- 436
CRAGI           HLFPTMPRHNLGKIAPLVKSLCKK-HNLPYRCSPLFTAFGDIVRMLKKSGE--------I 436
```

```
NEMVE       HLFPRVARQNLRSIQEKMKLLCKK-HGLPYRSKSFVDANIEVIQCLKDTAEKSKCFSPKI 447
MONBE       HLFPTMPRYKYRALVPIVRQWAKA-NGLVYKSDTLPVMLGEHVATLKKAGEMAHREDT-- 377
            **** : :         :         .   . . :     :     *.    :

HOMSA       WLDAYLHK 444
XENTR       WLDAYLHK 446
DANRE       WLDAYLNK 444
DROME       -------- 436
CRAGI       WYDAYYHG 444
NEMVE       WDSVHCIG 455
MONBE       -VDPYALS 384
```

Supplementary Figure 3. Alignment of FADS2 and FADS-like enzyme sequences from indicated species.
Residues in red: transmembrane (TM) domain. Residues highlighted in yellow: Cyt b5-like heme/steroid binding domain, and in green: fatty acid desaturase (FAD) domain. Sequences were aligned with CLUSTAL O(v1.2.4) multiple sequence alignment tool.

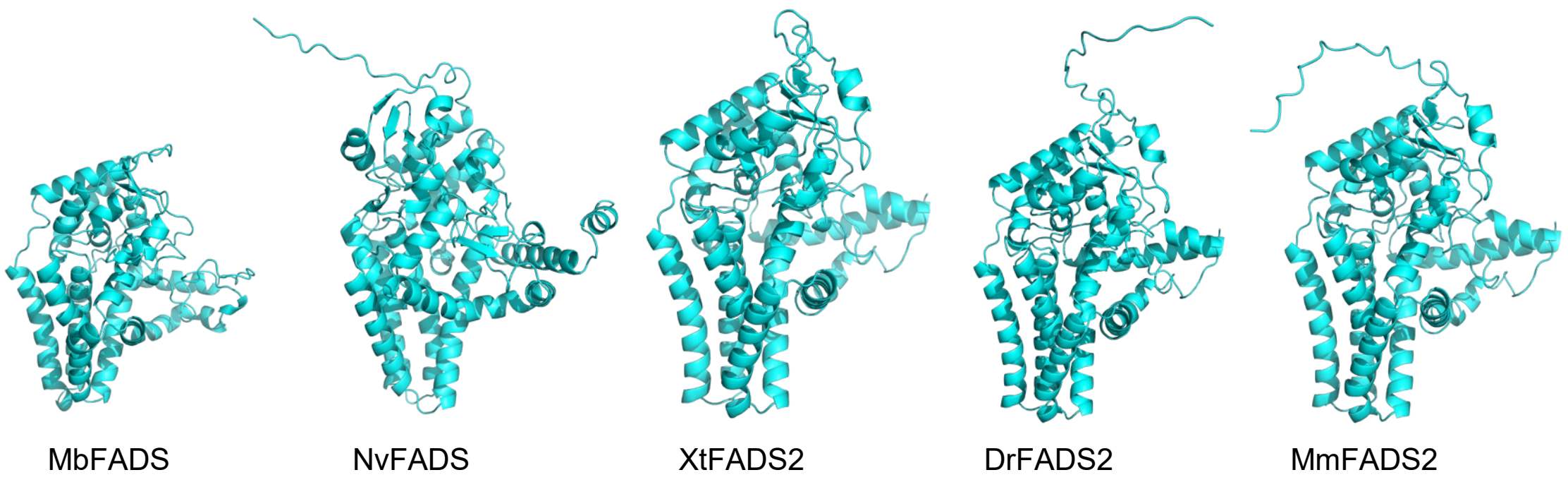

Supplementary Figure 4. Comparative analyses of FADS homologs.
3D Structures of FADS2/FADS-like enzymes from the indicated species, as predicted by Alphafold2, were rendered using Pymol.

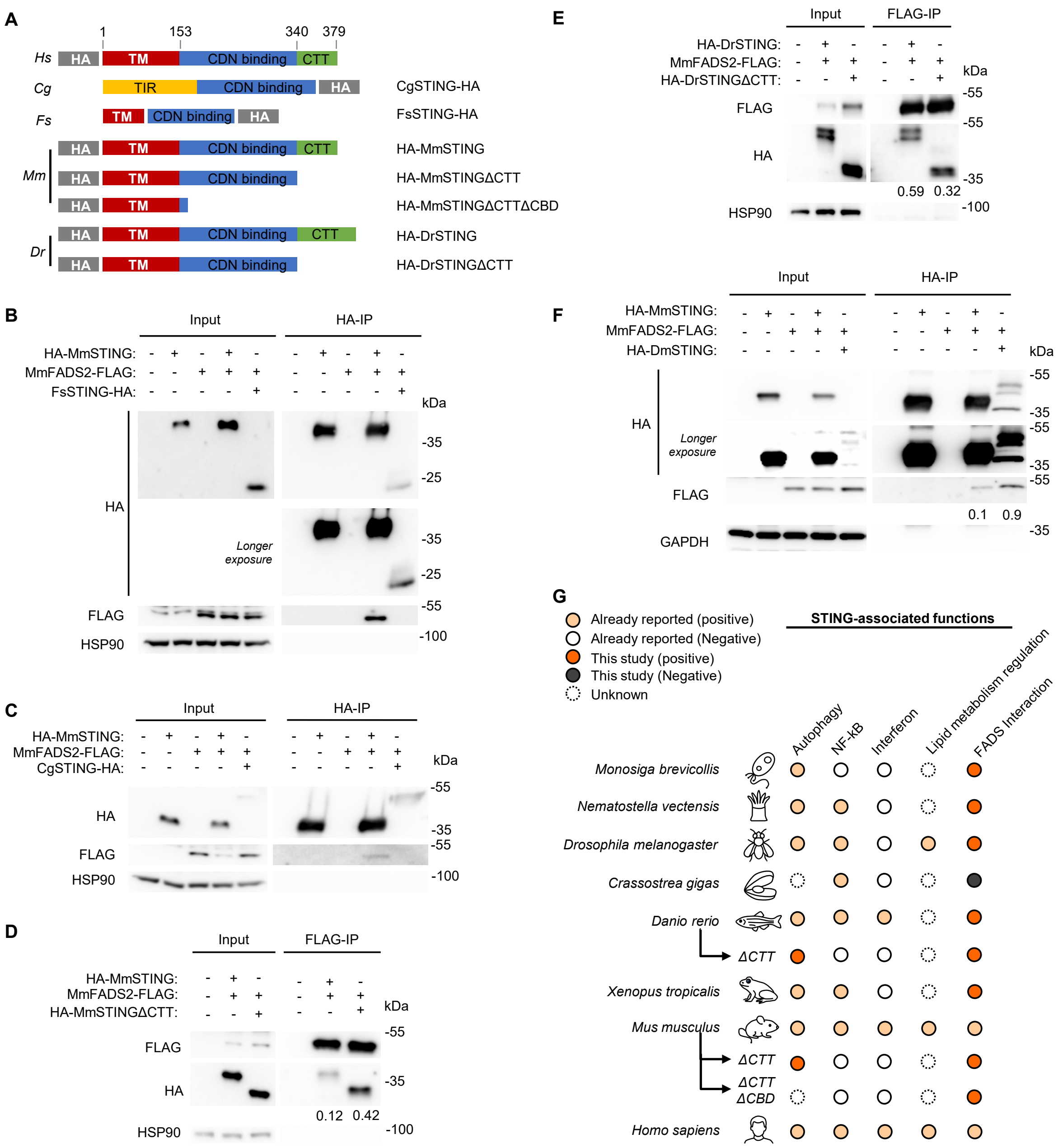

Supplementary Figure 5. The CTT is dispensable for the interaction of STING with FADS2.
(A) Schematics of HA-tagged STING homologs with rare domains, STINGΔCTT, or STINGΔCTTΔCBD mutants, roughly aligned to HsSTING.
(B) HA-immunoprecipitation was performed on whole-cell extracts (WCEs) from HEK 293T cells transiently co-expressing MmFADS2-FLAG with FsSTING-HA, HA-MmSTING, or none (control). Inputs and eluates from the immunoprecipitation were analyzed by Western blot (WB).
(C) As in B, except that cells co-expressed MmFADS2-FLAG with CgSTING-HA, HA-MmSTING, or none (control).
(D) FLAG-immunoprecipitation was performed on WCEs from HEK 293T cells transiently co-expressing MmFADS2-FLAG with HA-MmSTINGΔCTT, HA-MmSTING, or none (control).
(E) As in D, except that cells co-expressed MmFADS2-FLAG with HA-DrSTINGΔCTT, HA-DrSTING, or none (control).
(F) As in B, except that cells co-expressed MmFADS2-FLAG with HA-DmSTING, HA-MmSTING, or none (control).

(G) Scheme summarizing the data obtained in the study. STING-related functions are indicated, and their status at before (already reported) and after (this study) our analyses. ‘positive’ indicates a process regulated by STING, ‘negative’ indicates a process not regulated by STING.

WBs are representative of at least n=3 independent experiments. In panels B and F, two exposures of the anti-HA blots are shown. The ‘normalized binding index’ is indicated below the panel showing the co-immunoprecipitated protein.

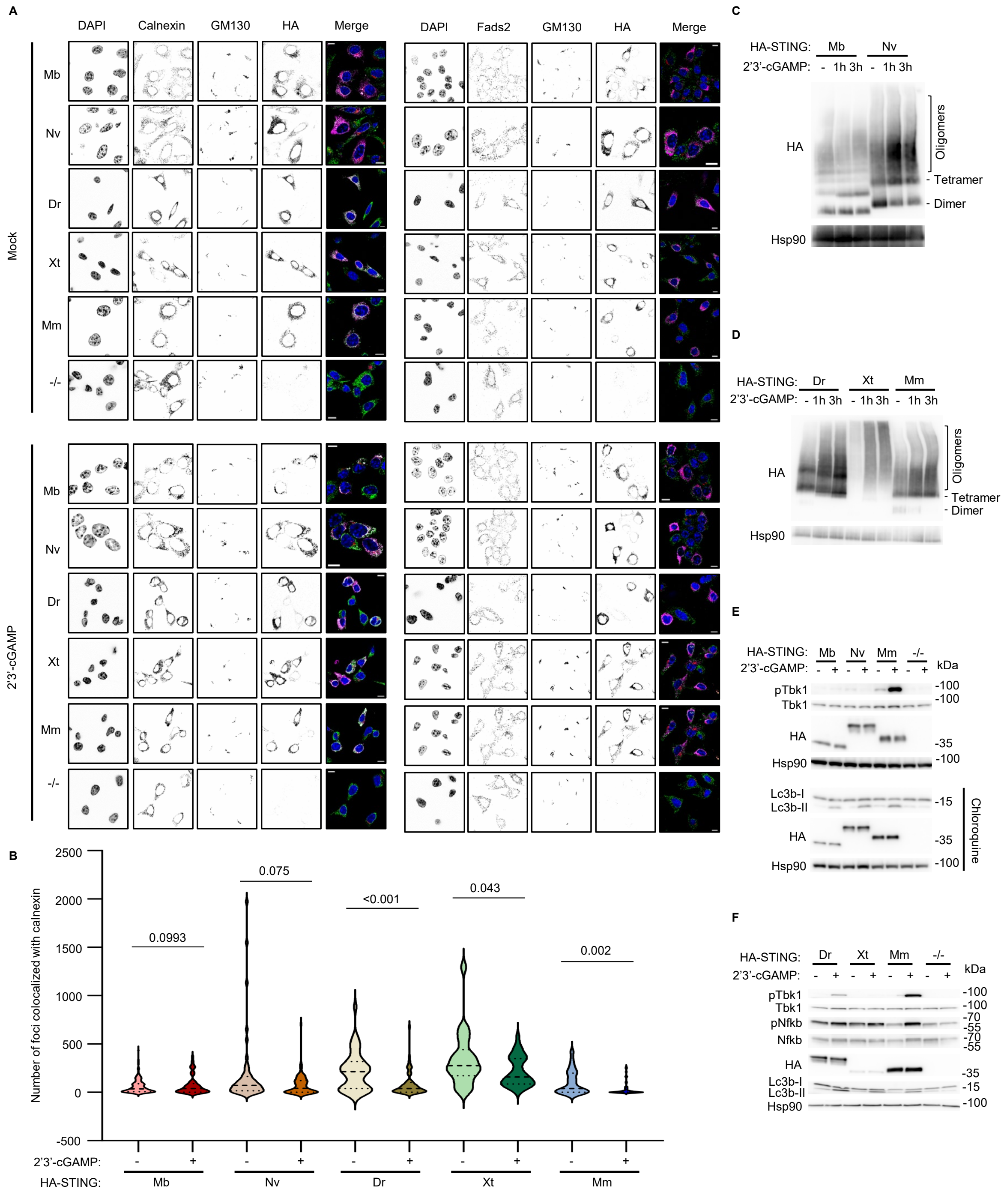

Supplementary Figure 6. The STING homologs are functional in MEF$^{Sting-/-}$ cells.

(A) Immunofluorescence analysis of MEF^{MbSTING}, MEF^{NvSTING}, MEF^{DrSTING}, MEF^{XtSTING}, MEF^{MmSting}, and MEF$^{Sting-/-}$, with or without stimulation with 10 µM 2'3'-cGAMP, using antibodies against the indicated proteins and DAPI nuclear staining. Scale bar (in white): 10 µm. Representative images (n=3 independent experiments).

(B) Bean-plot of the number of indicated HA-STING foci colocalized with calnexin with or without (control) 2'3'-cGAMP treatment. A minimum of 110 foci were counted per condition (from n=3 biological replicates). p-values were determined by two-tailed Mann-Whitney test.

(C) Oligomerization status of the indicated non-vertebrate STING homologs stably expressed in MEF$^{Sting-/-}$ treated or not (control) with 10 µM 2'3'-cGAMP for 1h or 3h, were analyzed by Western blot (WB) of Blue-Native PAGE from whole cell extracts (WCE).

(D) As in C, except that the indicated vertebrate STING homologs were analyzed.

(E) WB analysis of WCEs from $MEF^{MbSTING}$, $MEF^{NvSTING}$, $MEF^{MmSting}$, and $MEF^{Sting-/-}$, stimulated or not with 10 µM 2’3’-cGAMP.
(F) As in E, except that $MEF^{DrSTING}$, $MEF^{XtSTING}$, $MEF^{MmSting}$, and $MEF^{Sting-/-}$, were analyzed and 2’3’-cGAMP treatment was performed after pre-treatment with 20 µM chloroquine (bottom panel).

All WBs are representative of n=3 independent experiments. Control group is *Sting-/-* except otherwise stated.

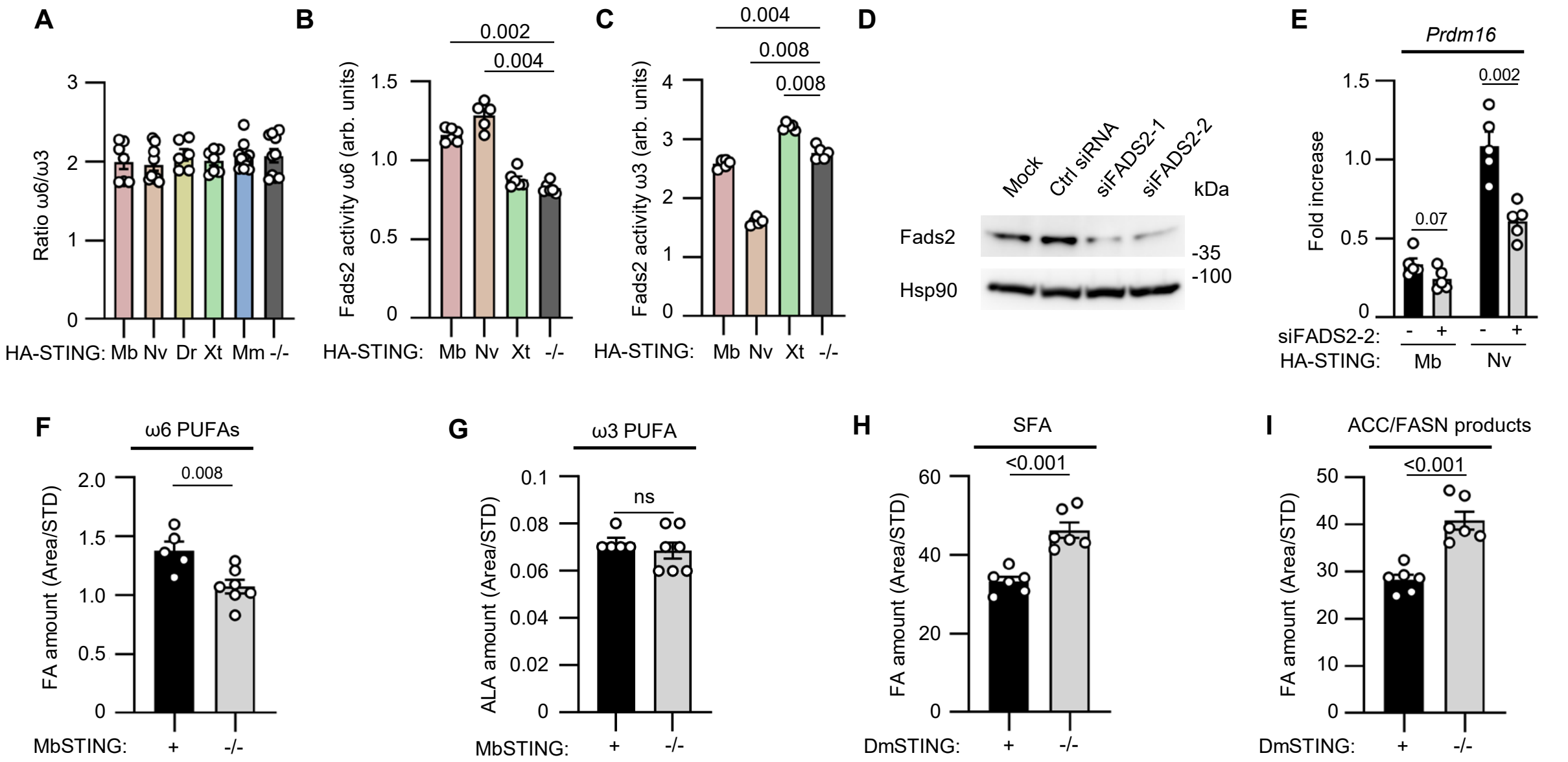

Supplementary Figure 7. STING homologs differentially modulate PUFA pools.

(A) Mean omega-6 to omega-3 PUFAs ratio measured from MEF^{MbSTING}, MEF^{NvSTING}, MEF^{DrSTING}, MEF^{XtSTING}, MEF^{MmSting}, and MEF$^{Sting-/-}$. n=7(MEF^{MbSTING}), n=9(MEF^{NvSTING}), n=6(MEF^{DrSTING}), n=7(MEF^{XtSTING}), n=12(MEF^{MmSting}), n=10(MEF$^{Sting-/-}$) biological replicates. p-values were determined by two-tailed Mann-Whitney test.

(B) Mean ^{13}C-labeled omega-6 LC-PUFAs to ^{13}C-LA ratio measured in MEF^{MbSTING}, MEF^{NvSTING}, MEF^{XtSTING}, and MEF$^{Sting-/-}$, treated with ^{13}C-LA. n=6(MEF^{MbSTING}, MEF^{XtSTING}, and MEF$^{Sting-/-}$), n=5(MEF^{NvSTING}) biological replicates. p-values were determined by two-tailed Mann-Whitney test.

(C) As in B, but showing mean ^{13}C-labeled omega-3 PUFAs to ^{13}C-ALA ratio after treatment with ^{13}C-ALA. n=6(MEF^{MbSTING}), n=5(MEF^{NvSTING}, MEF^{XtSTING}, and MEF$^{Sting-/-}$) biological replicates. p-values were determined by two-tailed Mann-Whitney test.

(D) WT-MEF cells were treated with 10 nM control siRNA or FADS2-targeting siRNAs (siFads2-1 and siFads2-2), prior to Western blot (WB) analysis. WB representative of n=2 independent experiments.

(E) *Prdm16* mRNA levels in MEF^{MbSTING} and MEF^{NvSTING}, following treatment with 10 nM siFADS2-2 or control siRNA, expressed as mean fold increase over mock transfected MEF^{NvSTING}. n=5 biological replicates.

(F) Mean omega-6 PUFAs summation from WT (n=5 biological replicates) and *STING-/-* (n=7 biological replicates) *M. brevicollis*.

(G) As in F, except that mean ALA levels in WT and *STING-/- M. brevicollis* are shown. Biological replicates are as in F.

(H) Mean saturated-FAs (SFA) summation from control and *STING-/- D. melanogaster* heads. n=6 biological replicates.

(I) As in H, except that mean acetyl-CoA carboxylase (ACC) and fatty acid synthase (FASN) enzyme products summation are shown. Biological replicates are as in H.

Error bas are ± standard error of the mean (SEM), p-values (shown above bars) were determined by two-tailed Student's t test, and control group is *Sting-/-* except otherwise stated.

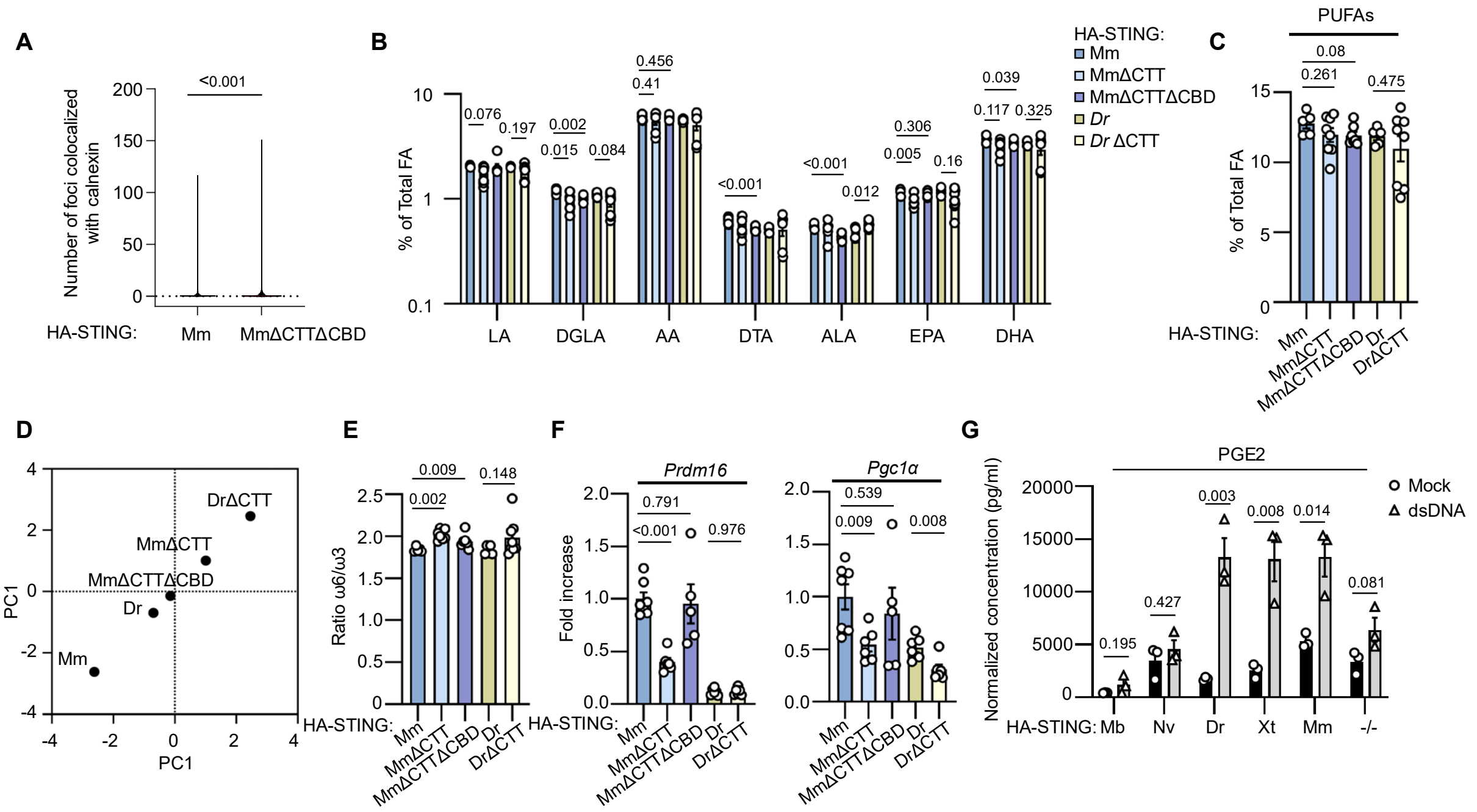

Supplementary Figure 8. Vertebrate STING mutants differentially modulate PUFA pools.

(A) Bean-plot showing number of HA-tagged MmSTING and MmSTINGΔCTTΔCBD foci colocalized with calnexin from immunofluorescence analysis of MEF^{MmSting} and MEF$^{MmSting\Delta CTT\Delta CBD}$. At least 1326 foci were counted per condition. n=3 biological replicates.

(B) Mean relative quantities of the indicated PUFAs in MEF^{MmSting}, MEF^{DrSTING}, MEF$^{MmSting\Delta CTT}$, MEF$^{MmSting\Delta CTT\Delta CBD}$, and MEF$^{DrSTING\Delta CTT}$, expressed as percentage of total fatty acids. n=5(MEF^{MmSting}, MEF^{DrSTING}), n=8(MEF$^{MmSting\Delta CTT}$, MEF$^{DrSTING\Delta CTT}$), n=7(MEF$^{MmSting\Delta CTT\Delta CBD}$) biological replicates.

(C) Mean summation of FADS2 PUFA products measured in B, expressed as percentage of total fatty acids. Biological replicates are as in B.

(D) PC score plot from principal component analysis performed on data from B. Biological replicates are as in B.

(E) Mean omega-6 to omega-3 PUFAs ratio measured from the data in B. Biological replicates are as in B. p-values were determined by two-tailed Mann-Whitney test.

(F) *Prdm16* (left) and *Pgc1α* (right) mRNA levels in the indicated cell lines are expressed as mean fold increase over MEF^{MmSting}. n=7(MEF^{MmSting}), n=6(MEF$^{MmSting\Delta CTT}$, MEF^{DrSTING}, MEF$^{DrSTING\Delta CTT}$), n=5(MEF$^{MmSting\Delta CTT\Delta CBD}$) biological replicates.

(G) Mean PGE2 levels in MEF^{MbSTING}, MEF^{NvSTING}, MEF^{DrSTING}, MEF^{XtSTING}, MEF^{MmSting}, and MEF$^{Sting-/-}$ (control), following treatment with 1 µg/ml dsDNA or not. n=3 biological replicates.

Error bas are ± standard error of the mean (SEM), MEF^{MmSting} is the positive control, p-values (shown above bars) were determined by two-tailed Student's t test, except when otherwise stated.

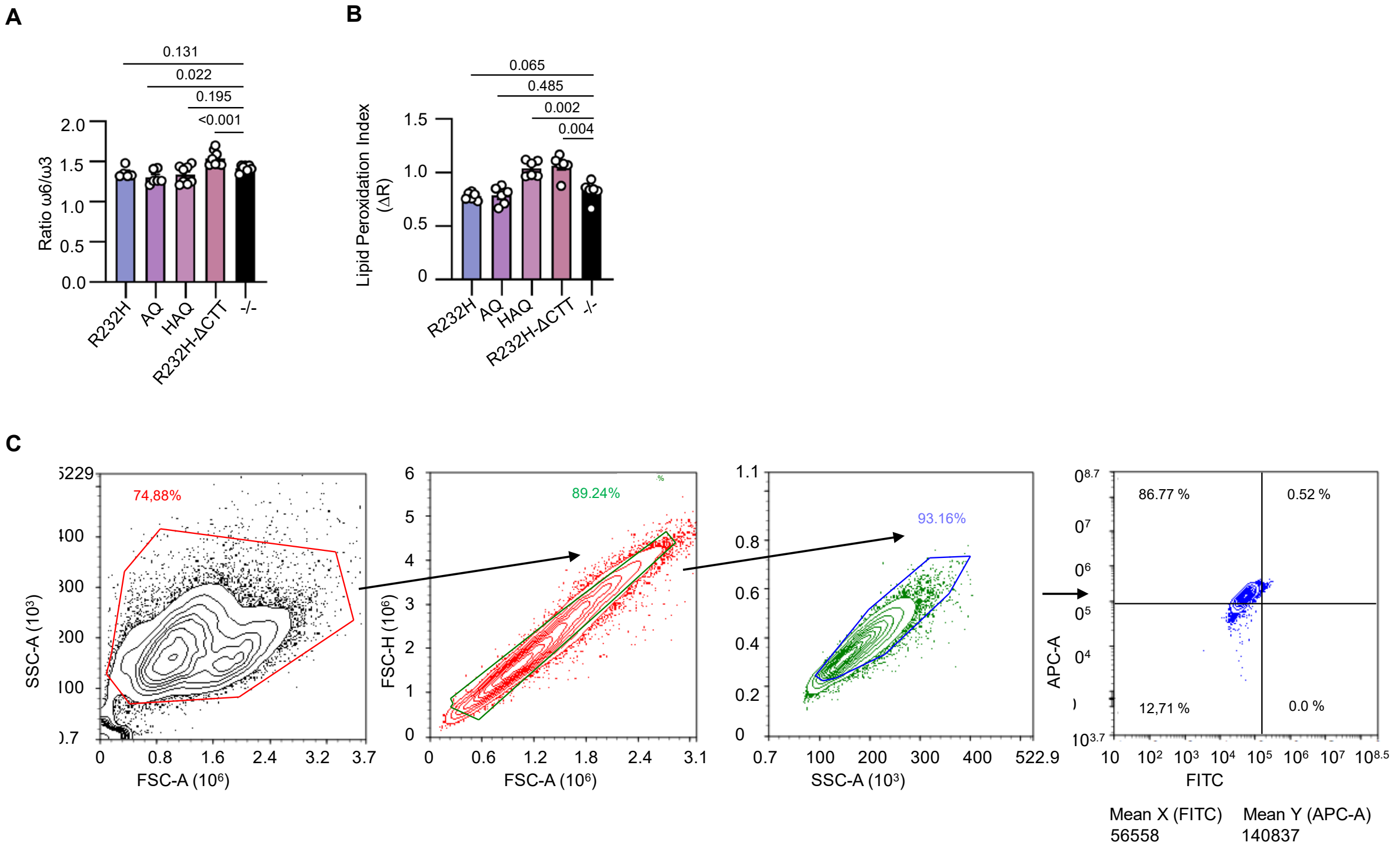

Supplementary Figure 9. Human STING haplotypes play a role in PUFA metabolism

(A) Mean total omega-6 to omega-3 PUFAs ratio is expressed as a percentage of total fatty acids in THP-1$^{\textit{HsSTING-R232H}}$ (n=5), THP-1$^{\textit{HsSTING-AQ}}$ (n=6), THP-1$^{\textit{HsSTING-HAQ}}$ (n=8), THP-1$^{\textit{HsSTING-R232H-}\Delta\textit{CTT}}$ (n=8), and THP-1$^{\textit{STING-/-}}$ (n=7). n are independent biological replicates.

(B) Mean lipid peroxidation index (ΔR) of the cell lines indicated in A. n=6 biological replicates.

(C) Representative gating strategy of MEF (presented in Figure 4H) and THP-1 cells (presented in Supplementary Figure 9B). Mean FITC and APC-A values were used to calculate the lipid peroxidation index. Created with NovoExpress Software.

Error bas are ± standard error of the mean (SEM), p-values (shown above bars) were determined by two-tailed Mann-Whitney test, and control group is *STING-/-* except otherwise stated.

Supplementary Table 1. Comparison matrix for MEF Sting-/- overexpressing HA-STING. Values are ranked in descending order: dark green, light green, white, light blue, dark blue.

		STING expression index		STING-FADS2 binding	Functional output				
		WB mean	IF mean	binding index	prdm16	pgc1a	PUFA	PGE2	6-keto-PGF1α
1	Mb	38.02	17.87	1.23	12.93	4.59	13.59	238.62	6147.30
2	Nv	70.70	69.92	0.32	36.30	6.99	12.78	2816.69	6444.57
3	Dr	82.24	44.66	0.10	0.05	0.17	9.75	982.02	14.59
4	Xt	29.85	31.43	0.89	0.17	0.16	8.94	858.31	20.52
5	Mm	90.44	54.57	0.89	0.57	0.38	9.34	852.90	35.60

Supplementary Table 2. STING and FADS2 homolog sequences.

Name	Uniprot accession code	Sequence
FsSTING	P0DUD7	MEKIYTWLKTNSYIVHHVSTSLNIISFIIVLIWIFESTIKEKLNII<br>FTVNLEAIVVFISILIVGLNQLLQKLLIEAEYSPAFALAVGYFK<br>NFIFPAITQIKENGEVNPKICIYKPKHFDELTSTNIDMIKAELT<br>NKKYNLSEINLSLKGARARDILTLNKKSKIHSYFDFPNTLLSL<br>YSYVDFKIASSNNNSSELKKKKFVELLIEQFYLKLNELIQEN<br>NLTNNITFCDKNLQGL
MbSTING	A9UUE7	MMVNLSDLSHLSQRGWAQVFVTLAAAAISIFYFSFSPFEVA<br>AGICASIAAAGAVPLLFDAVHYLIAFCISAPDARPPLRTVWT<br>KTRLQRWSGLSILTFIVLACGLYFASPSPMSASNLFAVFAL<br>SLCNSVFSAMVAPRCGRLEVVDLATHPNTVLFRDFGAASA<br>MSYWHGYLQHIAGDRAALEERFRDSDVGGMRVPRKLYIL<br>VPQDCEVNASDNKYEGLKATEHYISPKPITIGGVVDREMG<br>KHTLYTPSESAETASVAFAMELASPLNTMKNALKDAAEGLL<br>ELQSEAFYLTLRGILKQEGVLDQGNDEGMIKLVWGKNRSE<br>VLKKMRAEDQYAMP
NvSTING	A7SLZ2	MRRAEENNGFGTIPKRRNQHTPFYASIGMIVVIIVAFTSYHI<br>TSYGDDRNRAMRQYSFTFSLAYLAFLVGELLRRCCLFAEE<br>YRHIETRYNGSLKKAIQTTFSFGHNNVLFVASLLFFVVFVAS<br>NDPNGSSSVIQGNSTAEPHTEMRQTSGWQGLWGQFIISAL<br>LTPLVVHLLGLRELSKVEESQLNEKENKNVADGLAWSYYF<br>GYLKFVLPELEKQIEKTSKFRSKEKFVKKMFILIPSNCFWDD<br>KIPGSDYDPQNRITFEGNTEPLEKTRGGVFLRHYKHSVYEI<br>KDGENEPWFCIMEYATPLLTLYDMSVAQPGELSREERDA<br>QVVVFLRKLQDILEGDRACQGKYELVTFSPDRDLADVMLR<br>KLKDSELEIGG
CgSTING	P0DUE1	MEKNGAHSFLSDTPVTSLTMSVPVLRHPHVYHAFISYCAD<br>ADTSHARTILDSVESRGFTCCFAERDFLPGECTSDVVVDAI<br>HCSKNVILVISPASLQSEWSKFEMLMAVDDSHQRNNVCLV<br>PVLLGGVKVDDLPPPLRPLTCIELMDDFRNTDDIIQAISKPE<br>DTWESLLPVGNLAHGFAWGYYYGYLKIILPDLDKTVRQWR<br>RVNNAEGRMSEKLFLFFPQSCRCRDSIADESSLIKHRGHL<br>PIITKDRAGIIERQYKNTIYSVTDDNGEDYFFAGEYIGVIHTM<br>FEMEQNATTGLQTREKYVQSMRFYLTLKRILDTDPECSKK<br>CKIVFYKDVNNSSDAMPKLICNEIKNQLRKESSDDTTVCMT<br>PFNSPFPSISSPDFARCSLKSPSSTNMVKSEPNIYREESGK<br>TKSVERG
DmSTING	A0A0B4LFY9	MAIASNVVEAGNAVRAEKGRKYFYFRKMIGDYIDTSIRIVAT<br>VFLADLLLRLYRCVVEYGSNGRYYLPED<br>RLWIILRRSCTYNNRSIYLIVGFLLVAFFRISVTGNYRNVMP<br>TTLFLFQMPLYWIWSFTDMDQSTLSYSH<br>WIRDSHGLDYAAGMASNYFHGYLKLSLPERKDDGLKHRLA<br>MYEDKNNVTFGIKRLVILIPDEMFVNGVLESHLLDKAEPLET<br>QFINRAGVYRPFKHDVYRMNKKVNGRTYYFAVEGATPMIS<br>FFDATYSNLSGTWQMQELKREIWIKFYKHLKELITTWPETR<br>DLVELIIYNSHDSKGNLVDVGELLVAHMQNKTKTIDEISN
DrSTING	E7F4N7	MSVMGEDALVPRARSRLPVMCAAGLGFLTLAVAWLLDSD<br>KFSERAGIIAFGLMLERFIYCICLLAEELLFHSRQRYHGRMS<br>EIFRACFRGSGILGMCAIFLMLMLGGVSFSVKQWSHFNLM<br>CAGYMLLNSLGVLGPAPVEISEICEAKKMNVAHGLAWSFYI<br>GYLKFLLPALEVNVREYSRRERLSSPRLHILLPLNARVPSK<br>PEEEDTNVVFHENLPDLKLDRAGVRKRSYTNSVYKITHNN<br>ETFSCILEYATPLLTLYQMSQESSAGFGERERKQQVLLFYR<br>TLSQILDNSLECRNRYRLILLNDEHTGDPHYLSRELFQNLK<br>QQDGEIFMDPTNEVHPVPEEGPVGNCNGALQATFHEEPM<br>SDEPTLMFSRPQSLRSEPVETTDYFNPSSAMKQN

XtSTING	A0A8J1J6F3	MASIRNTLATQNRQIIPERRGKRATKMACVLAIGSILFVWILGKGKYSGAQLIYRMAINFAISQGCCLVTCACELTEEIKHLHTRYNGHYWRALKASFNLSCAAFVTAILCYVFYEPKLMASLPLTIDITLTLLSWLFCWILGIQGPTPATISEITEIKQLNVAHGLAWSYYVGYLQFVLPALKESIQKFNEENHNLLKFPETCRLHILIPLSCRLYGDLKDVDENITFLKEIPPLYIDRAGIKGRVFKNNVYRILDEDGRPYNCIVEYATPLASLLKMTDIPSAAFSADDRLQQTKLFYRTLKDILENAHELQNTYRLIVYEDFPETKDHSRHLLSQEILKHIRQQHSEEYSML
MmSTING	Q3TBT3	MPYSNLHPAIPRPRGHRSKYVALIFLVASLMILWVAKDPPNHTLKYLALHLASHELGLLLKNLCCLAEELCHVQSRYQGSYWKAVRACLGCPIHCMAMILLSSYFYFLQNTADIYLSWMFGLLVLYKSLSMLLGLQSLTPAEVSAVCEEKKLNVAHGLAWSYYIGYLRLILPGLQARIRMFNQLHNNMLSGAGSRRLYILFPLDCGVPDNLSVVDPNIRFRDMLPQQNIDRAGIKNRVYSNSVYEILENGQPAGVCILEYATPLQTLFAMSQDAKAGFSREDRLEQAKLFCRTLEEILEDVPESRNNCRLIVYQEPTDGNSFSLSQEVLRHIRQEEKEEVTMNAPMTSVAPPPSVLSQEPRLLISGMDQPLPLRTDLI
MbFADS	A9UU07	MKICIDQEWYDLTKWAKYHPGGVRILERFDNQDATDHFYSLHSTDAIRKFKAMRPTETKEDVPEVLPIDASFRELRAKLMDDGWWDRDLLTELGILIPIFAMCIIGTALAWSHPVISVLLISVGMQQAGWLGHDMTHARDSRYNDFWLRYVSGWLNGFDRNWWSNKHNTHHVLTNHVNHDPDIHVQPILYLWAPLKQMDHFLRKYQYIYFPLPYTLLFASWRLESLKWSIANRDYKMFFLAILPGYVWLALLPFKVVLASILVSGFLVAIVVTMSHESEELLLEREPSYVTNQFLTTRDVQCLDWVTEYLFGGMQYQLEHHLFPTMPRYKYRALVPIVRQWAKANGLVYKSDTLPVMLGEHVATLKKAGEMAHREDTVDPYALS
NvFADS	A7RJB6	MTVALQRRTPKVYTLDEVKEHCSKGDCWVVVEDSVYDLSKWIGHHPGGELPILYMAGRECTDVFKAFHPAWVFTKKLPAFKIGKLDDTRKEKKETSLSEDFEKLRQEIIEGGGLQTNYWFYIRLASMLALLFASIIYCVVFSNNVYIQVAAGILVAVFWQQMAFIGHDAGHHAIFHDEQWDDRLGLVVGNLLTGVSIGWWKKSHNAHHVVTNSVELDPDIQHLPVLAVTDKFFNSIKSIYHDRVMHFDGLAKFFVRYQHHLYFLIMGLARFNLYAQSFLLVLSKERVKLRVMEFVTMVLFWTWYLTLCSYLPTWSTRFAFVFLAHFLAGIIHIQITLSHFSMETYNGLPLDAFKENRFLLSQMDTTMDIECDPNLDFFHGGLQFQFEHHLFPRVARQNLRSIQEKMKLLCKKHGLPYRSKSFVDANIEVIQCLKDTAEKSKCFSPKIWDSVHCIG
DrFADS2	Q9DEX7	MGGGGQQTDRITDTNGRFSSYTWEEVQKHTKHGDQWVVVERKVYNVSQWVKRHPGGLRILGHYAGEDATEAFTAFHPNLQLVRKYLKPLLIGELEASEPSQDRQKNAALVEDFRALRERLEAEGCFKTQPLFFALHLGHILLLEAIAFMMVWYFGTGWINTLIVAVILATAQSQAGWLQHDFGHLSVFKTSGMNHLVHKFVIGHLKGASAGWWNHRHFQHHAKPNIFKKDPDVNMLNAFVVGNVQPVEYGVKKIKHLPYNHQHKYFFFIGPPLLIPVYFQFQIFHNMISHGMWVDLLWCISYYVRYFLCYTQFYGVFWAIILFNFVRFMESHWFVWVTQMSHIPMNIDYEKNQDWLSMQLVATCNIEQSAFNDWFSGHLNFQIEHHLFPTVPRHNYWRAAPRVRALCEKYGVKYQEKTLYGAFADIIRSLEKSGELWLDAYLNK
XtFADS2	A0A6I8QWG3	MGMGGQSGEGCSSGNCVKPEARYSWEEIQKHNLKTDKWLVIERKVYNITQWVKCHPGGMRVIGHYAGEDATDAFHAFHPDKNFVRKFLKPLYVGELAENEPSQDRDKNAQQVEDFRALRKTAEDMGLFKSNPAFFIVYLFHILLIEFLAWCTLHYFGTGWIPTILTVLLLTISQAQAGWLQHDFGHLSVFKKSKWNHLIHKFIIGHLKGASANWWNHRHFQHHAKPNIFRKDPDVNMVNVFVLGDTQPVEFGKKRIKYLPYNHQHLYFFLIGPPLLIPIYFTIQI

		MKTMITRKDWVDLAWSVSYYARFFITFVPFYGVLGSLALLN<br>AVRFIESHWFVWVTQMNHLPMVIDHEKYKDWLETQLAATC<br>NIEPSFFNDWFSGHLNFQIEHHLFPTMPRHNYWKIAPLVRS<br>LCSKYNVTYEEKCLYHGFRDVFRSLKKSGQLWLDAYLHK
MmFADS2	Q9Z0R9	MGKGGNQGEGSTERQAPMPTFRWEEIQKHNLRTDRWLVI<br>DRKVYNVTKWSQRHPGGHRVIGHYSGEDATDAFRAFHLD<br>LDFVGKFLKPLLIGELAPEEPSLDRGKSSQITEDFRALKKTA<br>EDMNLFKTNHLFFFLLLSHIIVMESLAWFILSYFGTGWIPTL<br>VTAFVLATSQAQAGWLQHDYGHLSVYKKSIWNHVVHKFVI<br>GHLKGASANWWNHRHFQHHAKPNIFHKDPDIKSLHVFVLG<br>EWQPLEYGKKKLKYLPYNHQHEYFFLIGPPLLIPMYFQYQII<br>MTMISRRDWVDLAWAISYYMRFFYTYIPFYGILGALVFLNFI<br>RFLESHWFVWVTQMNHLVMEIDLDHYRDWFSSQLAATCN<br>VEQSFFNDWFSGHLNFQIEHHLFPTMPRHNLHKIAPLVKSL<br>CAKHGIEYQEKPLLRALIDIVSSLKKSGELWLDAYLHK

Supplementary Table 3. Oligonucleotide sequences.

**Name**	**Sense sequence**	**Anti-sense sequence**
Hsp90	GTCCGCCGTGTGTTCATCAT	GCACTTCTTGACGATGTTCTTGC
Il6	GACTTCCATCCAGTTGCCTTCT	TCCTCTCCGGACTTGTGAAGTA
Oas1	TGCATCAGGAGGTGGAGTTTG	ATAGATTCTGGGATCAGGCTTGC
Ifnβ	GAATGGGAGGCTTGAATACTGCCT	TAGCAAAGATGTTCTGGAGCATCTC
Prdm16	CAGCACGGTGAAGCCATTC	GCGTCGATCCGCTTGTG
Pgc1α	AAAGGATGCGCTCTCGTTCA	GGAATATGGTGATCGGGAACA
Fads2	ACTGGCTGGATTCCTACCCT	AGTCATGTTGCAGCCATCCA
siFads2 1	CCACCAGCAUGAAUACUUCUU	GAAGUAUUCAUGCUGGUGGUU
siFads2 2	AACCAACCACCUGUUCUUCUU	GAAGAACAGGUGGUUGGUUUU
siRNA Control	AUACGUGCAUCCAAGAGUCUU	GACUCUUGGAUGCACGUAUUU
dsDNA probe	ACATCTAGTACATGTCTAGTCAGTAT<br>CTAGTGATTATCTAGACATACATGAT<br>CTATGACATATATAGTGGATAAGTGT<br>GG	CCACACTTATCCACTATATATGTCAT<br>AGATCATGTATGTCTAGATAATCACT<br>AGATACTGACTAGACATGTACTAGA<br>TGT

Supplementary Table 4. List of Antibodies.

**Antibody against**	**Dilution**	**Source**	**Identifier**
Flag	1:2000	Genscript	Cat#A00170-40; RRID:AB_914510
HA (clone 3F10)	1:1000 (WB)<br>1:100 (IF)	Roche	Cat#11867423001; RRID:AB_390918
HSP90 (clone C45G5)	1:1000	Cell Signaling	Cat#4877T; RRID:AB_2233307
GAPDH	1:5000	Proteintech	Cat#60004-1-Ig; RRID:AB_2107436
FADS2	1:5000	Invitrogen	Cat#PA587765; RRID:AB_2804393
pIRF3 (clone 4D4G)	1:500	Cell Signaling	Cat#4947S; RRID:AB_823547

IRF3 (clone D83B9)	1:1000	Cell Signaling	Cat#4302S; RRID:AB_1904036
pTBK1 (clone D52C2)	1:1000	Cell Signaling	Cat#5483S; RRID:AB_10693472
TBK1 (clone D1B4)	1:1000	Cell Signaling	Cat#3504S; RRID:AB_2255663
STING (clone D2P2F)	1:1000	Cell Signaling	Cat#13647S; RRID:AB_2732796
pNFKB (clone 93H1)	1:500	Cell Signaling	Cat#3033P; RRID:AB_331284
NFKB (clone D14E12)	1:1000	Cell Signaling	Cat#8242S; RRID:AB_10859369
LC3B	1:1000	Invitrogen	Cat#PA1-46286; RRID:AB_2234770
pIRF3 (clone EPR2346)	1:1000	Abcam	Cat#ab76493; RRID:AB_1523836
Goat anti-rat IgG HRP-conjugated	1:2000	Cell Signaling	Cat#7077S; RRID:AB_10694715
Goat anti-rabbit IgG HRP-conjugated	1:2000	Cell Signaling	Cat#7074S; RRID:AB_2099233
Horse anti-mouse IgG HRP-conjugated	1:2000	Cell Signaling	Cat#7076S; RRID:AB_330924
FADS2 (clone EPR27357-82)	1:50	Abcam	Cat#ab314317; RRID:AB_3665948
Calnexin	1:250	Millipore	Cat#AB2301; RRID:AB_10948000
GM130 (clone 35/GM130)	1:250	BD Biosciences	Cat#610823; RRID:AB_398142
dsRNA (J2)	1:300	Jena Bioscience	Cat#RNT-SCI-10010500
Goat anti-Mouse IgG (H+L) Alexa Fluor 594	NA	Invitrogen	Cat#R37121; RRID:AB_2556549
Goat anti-Rabbit IgG (H+L), Alexa Fluor 488	NA	Invitrogen	Cat#R37116; RRID:AB_2556544
Chicken anti-Rat IgG (H+L) Alexa Fluor 647	1:1000	Life Technologies	Cat#A-21472; RRID:AB_2535875
Goat anti-Mouse IgG (H+L), Alexa Fluor 488	1:1000	Invitrogen	Cat#A11001; RRID:AB_2534069

Supplementary Table 5. Table summarizing the retention times (RT) and mass/charge (m/z) ratios of each PFB-FA analyzed.

**Compound**	**RT (min)**	**m/z**	**Compound**	**RT (min)**	**m/z**
C18:4 n-3	22,266	275,2	C18:4 n-3 $^{13}C_{18}$	22,259	293,2
C18:3 n-6	22,195	277,2	C18:3 n-6 $^{13}C_{18}$	22,185	295,3
C18:3 n-3	22,647	277,2	C18:3 n-3 $^{13}C_{18}$	22,640	295,2
C18:2 n-6	22,559	279,2	C18:2n-6-d4 (IS)	22,532	283,2
			C18:2 n-6 $^{13}C_{18}$	22,554	297,2
C20:5 n-3	24,802	301,2	C20:5 n-3 $^{13}C_{18}$	24,795	319,2
C20:4 n-3	25,183	303,2	C20:4 n-3 $^{13}C_{18}$	25,176	321,2
C20:4 n-6	24,724	303,2	C20:4 n-6 $^{13}C_{18}$	24,714	321,2
C20:3 n-6	25,109	305,2	C20:3 n-6 $^{13}C_{18}$	25,098	323,2
C22:6 n-3	27,248	327,2	C22:6 n-3 $^{13}C_{18}$	27,241	345,2
C22:5 n-3	27,594	329,3	C22:5 n-3 $^{13}C_{18}$	27,587	347,3
C22:5 n-6	27,184	329,3	C22:5 n-6 $^{13}C_{18}$	27,173	347,3
C22:4 n-6	27,520	331,3	C22:4 n-6 $^{13}C_{18}$	27,504	349,3
C24:6 n-3	29,860	355,3	C24:6 n-3 $^{13}C_{18}$	29,846	373,3
C24:5 n-3	30,186	357,3	C24:5 n-3 $^{3}C_{18}$	30,179	375,3
C24:5 n-6	29,793	357 3	C24:5 n-6 $^{13}C_{18}$	27,776	375,3
C24:4 n-6	30,113	359,3	C24:4 n-6 $^{13}C_{18}$	30,103	377,3
