# Supplementary material for: Evolutionary conservation analysis reveals a primordial function of STING as a regulator of lipid metabolism via FADS2
Source: Nat Commun. 2026 Aug 14;17:9771. doi: 10.1038/s41467-026-76833-5 (PMC13572491; doi:10.1038/s41467-026-76833-5)
Supplement: Supplementary file 3 [file 41467_2026_76833_MOESM3_ESM.pdf]

nature portfolio

Corresponding author(s): Nadine Laguette

Last updated by author(s): 09/06/2026

Reporting Summary

Nature Portfolio wishes to improve the reproducibility of the work that we publish. This form provides structure for consistency and transparency in reporting. For further information on Nature Portfolio policies, see our Editorial Policies and the Editorial Policy Checklist.

Statistics

For all statistical analyses, confirm that the following items are present in the figure legend, table legend, main text, or Methods section.

n/a	Confirmed	
☐	☒	The exact sample size (*n*) for each experimental group/condition, given as a discrete number and unit of measurement
☐	☒	A statement on whether measurements were taken from distinct samples or whether the same sample was measured repeatedly
☐	☒	The statistical test(s) used AND whether they are one- or two-sided<br>*Only common tests should be described solely by name; describe more complex techniques in the Methods section.*
☒	☐	A description of all covariates tested
☒	☐	A description of any assumptions or corrections, such as tests of normality and adjustment for multiple comparisons
☐	☒	A full description of the statistical parameters including central tendency (e.g. means) or other basic estimates (e.g. regression coefficient) AND variation (e.g. standard deviation) or associated estimates of uncertainty (e.g. confidence intervals)
☐	☒	For null hypothesis testing, the test statistic (e.g. *F*, *t*, *r*) with confidence intervals, effect sizes, degrees of freedom and *P* value noted<br>*Give P values as exact values whenever suitable.*
☒	☐	For Bayesian analysis, information on the choice of priors and Markov chain Monte Carlo settings
☒	☐	For hierarchical and complex designs, identification of the appropriate level for tests and full reporting of outcomes
☒	☐	Estimates of effect sizes (e.g. Cohen's *d*, Pearson's *r*), indicating how they were calculated

Our web collection on statistics for biologists contains articles on many of the points above.

Software and code

Policy information about availability of computer code

Data collection	No software was used for data collection.
Data analysis	AlphaFold v2.3.0 was used for protein structure preduction. PyMol v3.1.0 was used to render protein structures. GraphPad Prism v10.6.1 was used for data analyses, statistical tests, and plotting. Image Lab 6 software was used for western blot data acquisition and analysis. FIJI v2.9.0 was used for immunofluorescence and western blot image visualization and analysis. ZEISS ZEN software was used for immunofluorescence data acquisition. CellProfiler v4.2.8 was used for analysis and quantification of immunofluorescence images. Chromeleon 7 software used for analysis in GC. MSD ChemStation software (version E.02.02.1431) and MassHunter GC/MS Translator (version B.06.00.1116) was used for data acquisition and conversion in GC-MS. Data processing in GC-MS was done using MassHunter Quantitative Analysis software (version B.09.00). NovoExpress (version 2.2.0) was used to visualize and analyze flow cytometry data. Rstudio v4.5.2 and R packages KEGGREST, clusterProfiler, and pheatmap were used for RNA-seq analysis and plotting. TBLASTN or BLASTP web-tools were used to search NCBI or Ensembl. Microsoft Office 2019 package was used for miscellaneous purposes. SMART (EMBL) and InterProScan (EBI) were used for sequence and protein structure analysis. Protein sequence alignments and similarity computing were conducted using Clustal Omega (EBI). Synteny analysis was performed using Genomicus. BioRender was used for generating certain figures. CLUSTAL O(v1.2.4) was used for sequence alignment.

For manuscripts utilizing custom algorithms or software that are central to the research but not yet described in published literature, software must be made available to editors and reviewers. We strongly encourage code deposition in a community repository (e.g. GitHub). See the Nature Portfolio guidelines for submitting code & software for further information.

Data

Policy information about availability of data

All manuscripts must include a data availability statement. This statement should provide the following information, where applicable:

- Accession codes, unique identifiers, or web links for publicly available datasets
- A description of any restrictions on data availability
- For clinical datasets or third party data, please ensure that the statement adheres to our policy

All data are included in the Supplementary Information or available from the authors, as are unique reagents used in this Article. The raw numbers for charts and graphs are available in the Source Data file whenever possible. The GC lipidomics data generated in this study have been deposited in the Zenodo database under the accession code 21418191 (https://doi.org/10.5281/zenodo.21418190). The GC-MS lipidomics data generated in this study have been deposited in the Zenodo database under the accession code 21417924 (https://doi.org/10.5281/zenodo.21400879). The RNA-seq data used in this study are available in the Gene Expression Omnibus (GEO) database under accession code GSE174340 [https://www.ncbi.nlm.nih.gov/geo/query/acc.cgi?acc=GSE174340]. Requests for further information and resources should be directed to and will be fulfilled by the lead contact, Nadine Laguette (nadine.laguette@cnrs.fr).

Research involving human participants, their data, or biological material

Policy information about studies with human participants or human data. See also policy information about sex, gender (identity/presentation), and sexual orientation and race, ethnicity and racism.

Reporting on sex and gender	No human participant was enrolled for the present study.
Reporting on race, ethnicity, or other socially relevant groupings	No human participant was enrolled for the present study.
Population characteristics	No human participant was enrolled for the present study.
Recruitment	No human participant was enrolled for the present study.
Ethics oversight	No human participant was enrolled for the present study.

Note that full information on the approval of the study protocol must also be provided in the manuscript.

Field-specific reporting

Please select the one below that is the best fit for your research. If you are not sure, read the appropriate sections before making your selection.

[x] Life sciences [ ] Behavioural & social sciences [ ] Ecological, evolutionary & environmental sciences

For a reference copy of the document with all sections, see nature.com/documents/nr-reporting-summary-flat.pdf

Life sciences study design

All studies must disclose on these points even when the disclosure is negative.

Sample size	Experiments were repeated a minimum of 3 times (biological replicates).
Data exclusions	No data was excluded.
Replication	Experiments were repreated independently by different lab members, ensuring their reproducibility.
Randomization	Experiments reported herein mostly applied in vitro biochemistry, molecular and cell biology approaches where no sequencing were performed. Randomization and covariates were thus not taken into account.
Blinding	Experiments reported herein were mostly in vitro biochemistry, molecular and cell biology approaches. No blinding was performed.

Reporting for specific materials, systems and methods

We require information from authors about some types of materials, experimental systems and methods used in many studies. Here, indicate whether each material, system or method listed is relevant to your study. If you are not sure if a list item applies to your research, read the appropriate section before selecting a response.

Materials & experimental systems

n/a	Involved in the study	
	x	Antibodies
	x	Eukaryotic cell lines
x		Palaeontology and archaeology
	x	Animals and other organisms
x		Clinical data
x		Dual use research of concern
x		Plants

Methods

n/a	Involved in the study	
x		ChIP-seq
	x	Flow cytometry
x		MRI-based neuroimaging

Antibodies

Antibodies used

The primary antibodies used for western blot are : anti-Flag (1:2000, Genscript, A00170-40); anti-HA (1:1000, Roche, 11867423001); anti-HSP90 (1:1000, Cell Signaling, 4877T), anti-GAPDH (1:5000, Proteintech, 60004-1-Ig), anti-FADS2 (1:5000, Invitrogen, PA587765), anti-pIRF3 (1:500, Cell Signaling, 4947S) , anti-IRF3 (1:1000, Cell Signaling, 4302S), anti-pTBK1 (1:1000, Cell Signaling, 5483S), anti-TBK1 (1:1000, Cell Signaling, 3504S), anti-STING (1:1000, Cell Signaling, 13647S), anti-pNFKB (1:500, Cell Signaling, 3033P), anti-NFKB (1:1000, Cell Signaling, 8242S), anti-LC3B (1:1000, Invitrogen, PA1-46286), Total OXPHOS Antibody Cocktail (1:250, Abcam, ab110413), pIRF3 (1:1000, Abcam, ab76493).
Horseradish peroxidase (HRP)-conjugated secondary antibodies (Cell Signaling Technology) were used at 1:2000 dilution. Immunoreactive proteins were visualized by chemiluminescent detection using SuperSignal™ West Pico Plus or Femto reagents (Thermo Scientific). Secondary antibodies used in the study are: Goat anti-rat IgG (1:2000, Cell Signaling, 7077S), Goat anti-rabbit IgG (1:2000, Cell Signaling, 7074S), Horse anti-mouse IgG (1:2000, Cell Signaling, 7076S).
Primary antibodies used for immunofluorescence are: anti-FADS2 (1:50, Abcam, ab314317), anti-HA (1:100, Roche, 11867423001), anti-Calnexin (1:250, Millipore, AB2301), dsRNA (1:300; Jena Bioscience, Anti-dsRNA J2 RNT-SCI-10010500), and anti-GM130 (1:250, BD Biosciences, 610823). Secondary antibodies used are: Alexa Fluor 488-coupled goat anti-Rabbit IgG (Invitrogen, R37116), Alexa Fluor 594-coupled goat anti-Mouse IgG (Invitrogen, R37121), Alexa Fluor 488-coupled goat anti-mouse IgG (1:1000; Invitrogen, A11001), and Alexa Fluor 647-coupled chicken anti-Rat IgG (Invitrogen, A-21472), diluted in the blocking solution.

Validation

Commercial antibodies were used. Details on validation methods can be found on the manufacturer's website.

Eukaryotic cell lines

Policy information about cell lines and Sex and Gender in Research

Cell line source(s)

HEK 293T (ATCC, CRL-3216), MEFSting-/- (gift from Soren R. Paludan, Aarhus University), and THP1-Dual™ KO-STING (Invivogen, thpd-kostg).

Authentication

None of the cell lines used was authenticated. Gene invalidation was verified by WB.

Mycoplasma contamination

All cell lines tested negative for mycoplasma contamination.

Commonly misidentified lines
(See ICLAC register)

No commonly misindentified lines was used in the study.

Animals and other research organisms

Policy information about studies involving animals; ARRIVE guidelines recommended for reporting animal research, and Sex and Gender in Research

Laboratory animals

Drosophila melanogaster used were dSTINGL76GsTer11 isogenized to DrosDel w1118 background (obtained from Prof. Jean-Luc Imler, IBMC). Monosiga brevicollis WT was from ATCC (PRA-258).

Wild animals

The study did not involve wild animals.

Reporting on sex

Experiments were performed on equal numbers (1:1 ratio) of male and female drosophila (n=6 pools of 30 control or KO drosophila heads) and sex is not relevant to the study of the unicellular eukaryote Monosiga brevicollis (n=5 WT and n=7 KO, pools of 12 million cells). For this reason, the data collected here does not provide infomation so as to sex bias and can be assumed to be applicable to both sexes.

Field-collected samples

No sample was collected from the field.

Ethics oversight

The use of Drosophila melanogaster and Monosiga brevicollis did not require any ethical approval.

Note that full information on the approval of the study protocol must also be provided in the manuscript.

Plants

Seed stocks	No plant was used in the study.
Novel plant genotypes	No plant was used in the study.
Authentication	No plant was used in the study.

Flow Cytometry

Plots

Confirm that:

- [x] The axis labels state the marker and fluorochrome used (e.g. CD4-FITC).
- [x] The axis scales are clearly visible. Include numbers along axes only for bottom left plot of group (a 'group' is an analysis of identical markers).
- [x] All plots are contour plots with outliers or pseudocolor plots.
- [x] A numerical value for number of cells or percentage (with statistics) is provided.

Methodology

Sample preparation	The Image-iT Lipid Peroxidation Kit (Invitrogen) was used for ratiometric assessment of lipid peroxidation by flow cytometry. MEF or ThP1 cells were stained with 10 µM Image-iT® Lipid Peroxidation Sensor (BODIPY® 581/591 C11) for 30 mins at 37°C. Cells were washed and resuspended in ice cold FACS buffer (PBS, 10% FBS, 0.5 mM EDTA).
Instrument	NOVOCYTE ACEA
Software	NovoExpress v2.2.0
Cell population abundance	N/A
Gating strategy	Cell debris and doublets/aggregates were removed by plotting FSC-H vs FSC-A and subsequently SSC-H vs SSC-A.

- [x] Tick this box to confirm that a figure exemplifying the gating strategy is provided in the Supplementary Information.
